# Identifying and preventing degradation in flavin mononucleotide-based redox flow batteries via NMR and EPR spectroscopy

**DOI:** 10.1038/s41467-023-40649-4

**Published:** 2023-08-25

**Authors:** Dominic Hey, Rajesh B. Jethwa, Nadia L. Farag, Bernardine L. D. Rinkel, Evan Wenbo Zhao, Clare P. Grey

**Affiliations:** 1 Yusuf Hamied Department of Chemistry, University of Cambridge, Cambridge, UK; 2 Present Address: Magnetic Resonance Research Centre, Institute for Molecules and Materials, Radboud University, Nijmegen, The Netherlands

**Keywords:** Batteries, Solution-state NMR

## Abstract

While aqueous organic redox flow batteries (RFBs) represent potential solutions to large-scale grid storage, their electrolytes suffer from short lifetimes due to rapid degradation. We show how an understanding of these degradation processes can be used to dramatically improve performance, as illustrated here via a detailed study of the redox-active biomolecule, flavin mononucleotide (FMN), a molecule readily derived from vitamin B2. Via in-situ nuclear magnetic resonance (NMR) and electron paramagnetic resonance (EPR) we identify FMN hydrolysis products and show that these give rise to the additional plateau seen during charging of an FMN-cyanoferrate battery. The redox reactions of the hydrolysis product are not reversible, but we demonstrate that capacity is still retained even after substantial hydrolysis, albeit with reduced voltaic efficiency, FMN acting as a redox mediator. Critically, we demonstrate that degradation is mitigated and battery efficiency is substantially improved by lowering the pH to 11. Furthermore, the addition of cheap electrolyte salts to tune the pH results in a dramatic increase in solubility (above 1 M), this systematic improvement of the flavin-based system bringing RFBs one step closer to commercial viability.

## Abstract (web summary)

Aqueous organic redox-flow batteries are known to suffer capacity loss via degradation of the redox-active species. Here, the authors use in situ methods to study the electrolyte flavin mononucleotide, identifying a redox mediator mechanism that mitigates capacity loss and a route to prevent its degradation.

## Introduction

Redox flow batteries (RFBs) are a promising energy storage system for grid-level storage, where low-cost and scalability are essential^1^. To date, many different organic molecules including quinones^2–4^, viologens^5,6^, phenazines^7,8^, and alloxazines^7,9^ have been investigated as potentially cheap RFB active molecules. Although a few molecules have shown a good performance in alkaline solution (pH 14)^7,8^, most organic molecules considered for RFBs generally experience degradation, reducing cell lifetime^1^. In 2016, Orita et al. ^10^ reported an RFB comprising flavin mononucleotide (FMN^3−^) at pH 14 as the anolyte against a potassium hexacyanoferrate K_4_[Fe^II^(CN)_6_] catholyte. The cell showed a capacity retention of 99% over the course of 100 cycles. Despite the encouraging capacity retention, an additional FMN reduction plateau appeared during charge, which was assigned to a dimerization process^10^. The process was not seen on discharge, leading to considerable cell hysteresis. Consequently, the resulting capacity retention and energy efficiency were not good enough for grid-scale storage systems, where even longer long-life times with minimal degradation and high coulombic efficiency are required. More recently, Nambafu et al. attached a 2,2,6,6- tetramethylpiperidinyl-N-oxyl (TEMPO) radical to FMN to form a bifunctional redox active material, which showed improved stability at neutral pH^11^. However, significant capacity loss was seen within 100 cycles, which was largely ascribed to degradation of the TEMPO functionality.

FMN is a commercially available, non-toxic biomolecule, readily derived from vitamin B2, motivating its further study in an RFB^10^. This molecule is also used in the food industry as an orange-red food color additive, utilized in Europe as E101a^12^; the sodium salt is commonly known as E106 and is found in foods for babies and young children as well as jelly, milk products, and sweet products^12^.

Here, we demonstrate a powerful strategy to study the degradation of FMN^3−^ by coupling in situ NMR and EPR techniques^4^. We explain how degradation, which we show involves the hydrolysis of FMN^3−^ rather than a dimerization process, leads to the additional charging plateau. We investigate the electrochemical behavior of hydrolyzed FMN^3−^ with in situ NMR and demonstrate that FMN^3−^ acts as a redox mediator, chemically reducing the hydrolyzed product, explaining the lack of an additional plateau on discharge yet good cycling behavior, despite degradation and poor reversibility of hydrolyzed FMN’s redox reactions. Lastly, we provide a strategy to avoid hydrolysis by lowering the pH. The battery performance is improved significantly and the FMN solubility is increased dramatically, by addition of a simple salt.

## Results and discussion

### Galvanostatic cycling of an FMN-based RFB

**Fig. 1:**
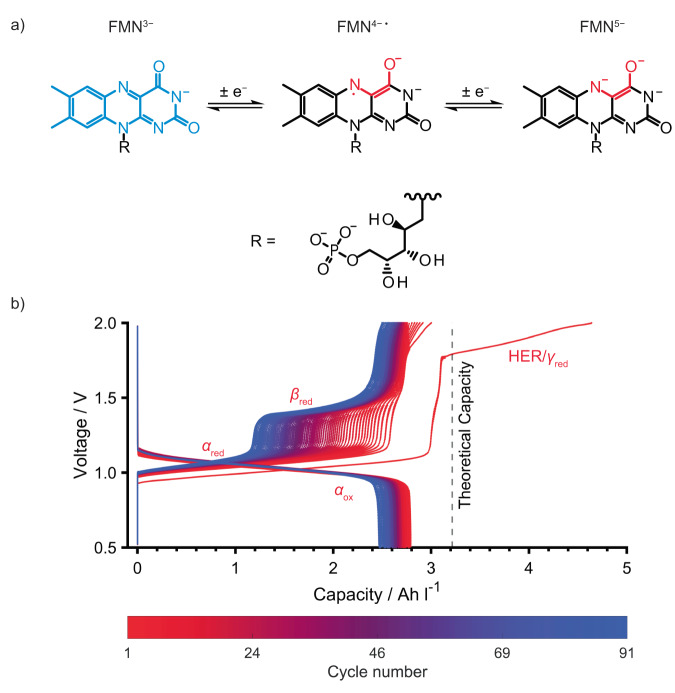
Galvanostatic cycling data of FMN^3−^ in an RFB at pH 14. **a** Two one-electron redox reaction mechanism of FMN^3−^ at pH 14. The ion on the left shows the skeletal structure of FMN^3−^ (with the alloxazine structural unit in blue). The fully deprotonated state that dominates at pH 14 is shown. The negative charge on the nitrogen in FMN^3−^ is in resonance with the two carbonyl groups. **b** Galvanostatic cycling of an RFB between 0.5 and 2.0 V_cell_ for the full cell, for 91 cycles with 60 mM FMN^3−^ in 1 M KOH/D_2_O as the anolyte (15 mL) and 0.2 M K_4_[Fe(CN)_6_] and 0.05 M K_3_[Fe(CN)_6_] in 1 M KOH/D_2_O as the catholyte (15 mL). A current density of ±10 mA cm^−2^ was used. The active area of the electrode was 5 cm^2^, giving a current of ±50 mA. The efficiencies are presented in Supplementary Note 2 and Fig. S12. The catholyte K_4_[Fe(CN)_6_]/ K_3_[Fe(CN)_6_], which operates with a single process at 0.5 V versus the standard hydrogen electrode (SHE)^10^, is in excess, and thus the different processes seen on charging of the full cell are assigned to those of the anolyte and are thus labeled, α_red_, β_red_ and γ_red_. Those on discharging are similarly labeled α_ox_.

To assess the cycling performance of FMN, an RFB was assembled comprising of 60 mM FMN^3−^ and 1 M KOH in D_2_O as the anolyte and 0.2 M K_4_[Fe(CN)_6_] with 0.05 M K_3_[Fe(CN)_6_] in 1 M KOH in D_2_O as the catholyte. The cycling data (Fig. 1b) shows a plateau at 1.02 V_cell_ (*α*_red_) and a second, sloping plateau or process starting at 1.71 V_cell_ (*γ*_red_) during the first charge (reduction of FMN^3−^); the theoretical capacity of 3.22 Ah L^−1^ was achieved (here: 3.18 Ah L^−1^) before the second plateau, *γ*_red,_ was completed (4.62 Ah L^−1^). On discharge (oxidation of FMN^5−^) only a single sloping plateau at 1.09 V_cell_ (*α*_ox_) was seen, and 2.79 Ah L^−1^ was recovered. On subsequent cycles, an additional charge plateau centered at 1.41 V_cell_ (*β*_red_) grew in prominence over 90 cycles, resulting in a steady drop in the voltaic efficiency (Fig. S9). The plateau *γ*_red_ decreased rapidly in capacity until the 15th cycle where it was no longer observed, after which the charge capacity stabilized at 2.80 Ah L^−1^. No new plateaus were observed during discharge.

Plateaus *α*_red_ and *α*_ox_ can be attributed to the reduction and oxidation of FMN^3−/5−^, and oxidation and reduction of Fe(CN)_6_^4−/3−^, respectively (Fig. 1a), no distinct plateaus for the separate one-electron reactions of FMN^3−/5−^ being seen. Plateau *γ*_red_ is tentatively assigned to water reduction (HER), which is corroborated by the formation of gas bubbles (observed in the tubing, Fig. S15). Deuterium evolution has been shown to commence at 1.20 V_cell_ vs. [Fe(CN)_6_]^3−/4−^ in a 2,6−dihydroxyanthraquinone/K_4_[Fe(CN)_6_] study^3^ and the literature shows that the thermodynamic potential for hydrogen evolution from water at pH 14 is around −1.2 V vs. SHE^3,4^. In this case, the water splitting commenced at higher voltages (1.71 V_cell_ vs. [Fe(CN)_6_]^3−/4−^) and we hypothesize that the hydrogen evolution is kinetically inhibited due to the carbon electrodes used in this work. The rapid decrease in this reaction with cycling is tentatively ascribed to a passivating film forming on the carbon paper used as the electrode. The reaction can be completely avoided by using a lower upper cut-off voltage of 1.7 V (see later).

The gradual appearance of plateau *β*_red_ suggests that a new reduction process is occurring, that is distinct from the reduction of FMN^3−^, or the semiquinone FMN^4−•^, to FMN^5−^. This process was seen in the earlier work of Orita et al.^10^ where they hypothesized that the two charging plateaus result from reduction of the monomer and dimer of FMN^3−^. They argued that due to the slower kinetics of the dimer oxidation, compared to that of the monomer, the discharge curve does not mirror the charge curve. We, however, propose that this process is associated with a degradation product of FMN^3−^. To identify the species giving rise to the plateaus observed in the cycling data, a combination of in situ NMR and EPR measurements were performed.

### In situ NMR/EPR studies of FMN as an anolyte

The redox reactions and degradation of FMN^3−^ during battery operation were followed by NMR/EPR spectroscopy (Supplementary Note 3, Fig. S13). Two different samples were studied: a freshly prepared (“fresh sample”) and a four-day-old (“aged sample”) FMN^3−^ solution.

**Fig. 2:**
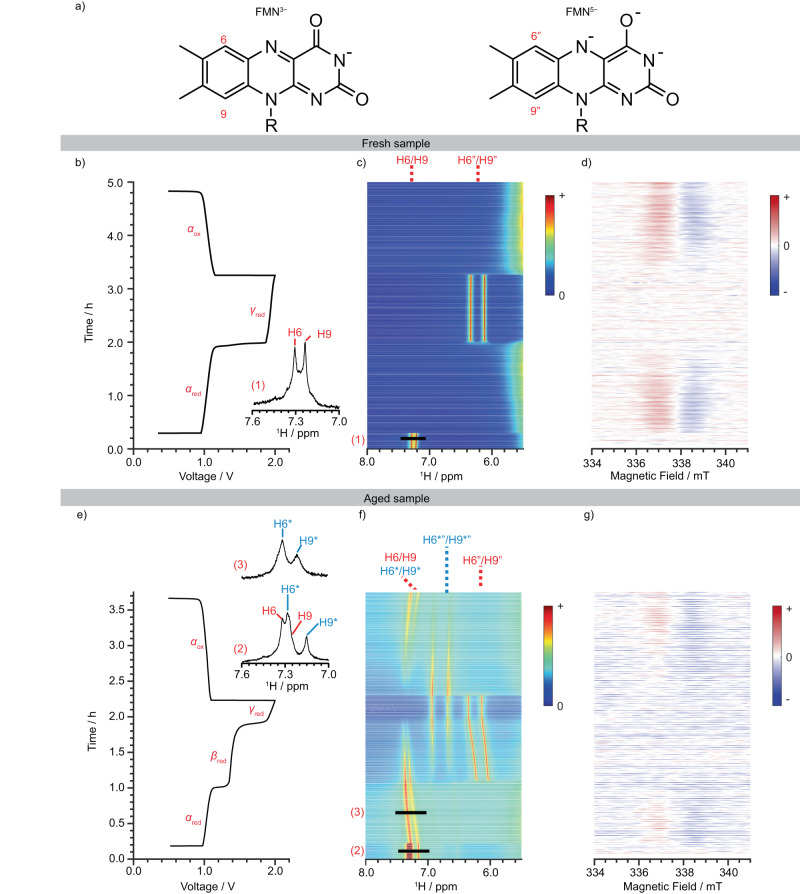
In situ EPR/NMR analysis of fresh and aged FMN^3^− in an RFB. **a** Labeling of aromatic proton positions of FMN^3−^ and FMN^5−^. **b** Voltage profile of a cell comprising freshly prepared 60 mM FMN^3−^ versus 0.2 M K_4_[Fe(CN)_6_] and 0.05 M K_3_[Fe(CN)_6_] in 1 M KOH/D_2_O. A constant current density of ±10 mA cm^−2^ (±50 mA) was applied during cycling between 0.5 and 2.0 V_cell,_ with the ﻿^1^H NMR (aromatic region) spectrum of the fresh 60 mM FMN^3−^ solution before cycling (1). **c** ^1^H NMR and **d** EPR spectra of the same cell (full NMR spectra in Fig. S14). **e** Voltage profile of a cell that used an aged (4 days) 60 mM FMN^3−^ electrolyte with the ^1^H NMR spectra of the aged solution before cycling (2) and during α_red_ (3). **f** ^1^H NMR and **g** EPR spectra (position of the slices (2) and (3) are marked on **f**; full NMR spectra in Fig. S17, 18).

The voltage profile (Fig. 2c) of a full cell containing freshly prepared FMN^3−^ (60 mM in 1 M KOH/D_2_O) anolyte and corresponding in situ ^1^H NMR and EPR spectra are shown in Fig. 2d and e, respectively. At OCV (0.51 V_cell_), the FMN^3−^ ring protons, H9 and H6, were clearly seen in the in situ ^1^H NMR spectra at 7.25 ppm and 7.33 ppm, respectively, the shifts correlating well to those obtained via ex situ NMR 1- and 2-dimensional NMR measurements (Supplementary Note 1.2 and 1.3, Fig. S7-9). No evidence for any radicals was seen by EPR spectroscopy. Upon charge (at start of the plateau *α*_red_), the alloxazine ring proton signals (H6, H9) in the ^1^H NMR spectra disappeared immediately (Fig. 2d), while a resonance (at 337.8 mT) grew in the EPR spectra (Fig. 2e), indicating the formation of a radical. The formation of radical species correlated with the disappearance of the ^1^H NMR signals at the beginning of the *α*_red_ plateau, the radical being assigned to FMN^4−•^, as shown with ex situ EPR measurements and DFT calculations (Supplementary Note 1.1, Figs. S1–6, 16). A reliable estimation of the radical concentration could not be obtained from this data, due to the low signal-to-noise of the EPR signal of FMN^4−•^, indicating that it was present in very low concentrations throughout cycling ([FMN^4−•^] < 5%; Fig. S3).

At the start of plateau *γ*_red_, two new ^1^H NMR signals at 6.12 ppm and 6.34 ppm appeared, which are assigned to the aromatic protons on FMN^5−^ (H6”, H9”). The EPR signal disappeared, indicating that all of the radicals have been reduced. No signals for FMN^3−^ were seen, confirming the complete reduction of FMN^3−^ to FMN^5−^ during plateau *α*_red_. The reverse of the trends during the *α*_red_ plateau was observed in both the NMR and EPR spectra during plateau *α*_ox_, the ^1^H NMR signals (H6”, H9”) disappearing due to the formation of FMN^4−•^, as confirmed by the appearance of a signal (at 337.8 mT) in the EPR spectra. Note that the signals H6 and H9 did not appear again at the end of *α*_ox_ since the cell was not held long enough at low voltages to remove all the radicals, the data shown here being part of a longer cycling study (with full cycling shown in Fig. S14). The in situ ^31^P NMR spectra show a similar trend of disappearing and reappearing signals (Fig. S19).

The voltage profile (Fig. 2g) of a full cell containing the aged sample surprisingly contained the higher voltage *β*_red_ plateau that was seen only after extended cycling of a fresh sample. Furthermore, the ^1^H NMR spectrum of the aged sample showed signs of FMN^3−^ degradation containing a new pair of ^1^H NMR signals at 7.15 ppm and 7.28 ppm (H6*, H9*; Fig. 2f-II), shifted by ~0.07 ppm from the FMN^3−^ resonances, in addition to those for FMN^3−^ (Fig. 2b-I). On charging the full cell, and similar to the fresh sample, the ^1^H NMR signals (Fig. 2h) for FMN^3−^ disappeared immediately and the EPR signal at 337.8 mT (Fig. 2i) due to FMN^4−•^ was observed (at start of the plateau *α*_red_). The H6* and H9* ^1^H NMR signals, by contrast, remained (Fig. 2f-III), but gradually shifted towards higher frequencies in part due to the change in the bulk magnetic susceptibility caused by the FMN^4−•^ radicals generated during plateau *α*_red_.

At the end of the *α*_red_ plateau, the H6” and H9” ^1^H NMR signals for FMN^5−^ appeared immediately, as was seen for the fresh sample. Not until plateau *β*_red_, however, did the signals H6* and H9* slowly disappear, their loss correlating with the gradual appearance of a new set of signals at 6.47 ppm and 6.83 ppm (H6*”, H9*”). That no EPR signals were seen during plateau *β*_red_, indicates that the reduction of the species giving rise to H6* and H9* does not generate any (EPR-observable) radicals. As before, no EPR signal was observed during *γ*_red_ and the intensity of the ^1^H NMR signals (H6”, H9”; H6*”, H9*”) remained constant.

On discharging and during plateau α_ox_, the ^1^H NMR signals for FMN^5−^ disappeared immediately. The signals for H6*” and H9*” disappeared more slowly, their intensity loss again correlating inversely with the appearance of the signals H6* and H9*. The EPR signal for FMN^4−•^ (337.8 mT) was observed again. The in situ ^31^P NMR spectra also show a similar trend of disappearing signals (Fig. S20). Thus, based on the in situ NMR and EPR data, a second species was formed during aging (or by extended cycling) that is closely related to, but exhibits different electrochemical behavior from, FMN^3−^.

### Hydrolysis of FMN

To determine the composition of the second species, we considered various degradation mechanisms of FMN during aging. First, we explored the photo-reduction of FMN^3−^ to FMN^4−•^^13^ as a possible degradation mechanism. Different concentrations of FMN^4−•^ were observed based on different degrees of illumination of the sample: FMN^4−•^ was generated within the first 30 min and was present in a higher concentration than found in a sample prepared in the dark, which exhibited, on the basis of its ex situ ^1^H NMR spectrum, little to no evidence of FMN^4−•^ (Fig. S1). However, no evidence for FMN^4−•^ was observed after 4 days, with the ^1^H NMR spectrum showing sharpened signals (Fig. S9). This indicates that the FMN^4−•^ can been re-oxidized (Supplementary Note 1.1), presumably by dissolved oxygen, and hence was no longer present in the aged sample.

**Fig. 3:**
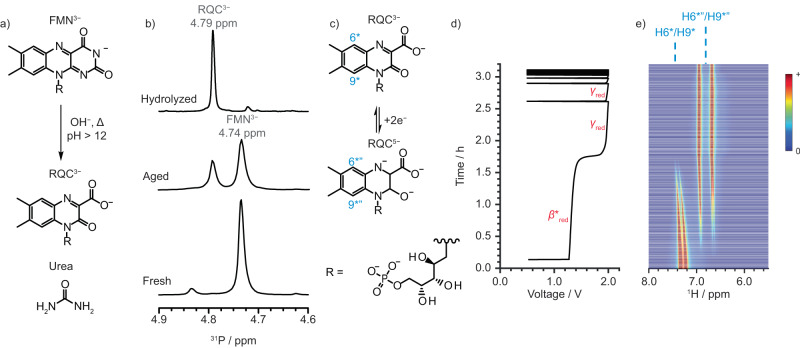
NMR analysis of hydrolyzed FMN^3−^. **a** Alkaline hydrolysis of FMN^3−^ to form RQC^3−^ as the product. **b** ^31^P NMR spectra of (bottom-to-top) fresh, aged, and hydrolyzed 60 mM of FMN^3−^ in 1 M KOH/D_2_O. **c** Suggested two-electron redox reaction of RQC^3−^ at pH 14. **d** Voltage profile of a 60 mM RQC^3−^ solution versus 0.2 M K_4_[Fe(CN)_6_] and 0.05 M K_3_[Fe(CN)_6_] in 1 M KOH/D_2_O full cell as a function of time; a constant current density of ±10 mA cm^−2^ (±50 mA) was applied during cycling between 0.5 and 2.0 V_cell_. **e** ^1^H NMR spectra of the anolyte (RQC^3−^/RQC^5−^). The EPR and the full ^1^H NMR spectra can be found in the supporting information (Figs. S31 and S32).

Secondly, FMN^3−^ has been reported to undergo hydrolysis under basic conditions (pH > 12), a process that can be accelerated at high temperatures^14^ (Supplementary Note 4.1, Fig. S21, 22, 29). To verify whether hydrolysis was also the source of the species formed after aging, a fresh solution of FMN^3−^ was heated to 90 °C for 2 h to ensure complete hydrolysis, after which it was characterized by NMR spectroscopy. The one- and two-dimensional NMR spectra (Fig. S21–27) and Infrared spectroscopy (Fig. S28) were consistent with the formation of 4-(D-ribo-2,3,4-trihydroxypentyl-5’-phosphate)−3-oxo-3,4-dihydroquinoxaline-2-carboxylate, RQC^3-^ (Fig. 3a), one of a number of hydrolysis products that have been proposed to form on hydrolysis of FMN^3−^ under different conditions (Supplementary Note 4.2, Fig. S22)^11,14,15^.

The ex situ ^31^P NMR spectrum (Fig. 3b) of the fresh sample was dominated by a signal at 4.74 ppm from to the phosphate group of FMN^3−^, with a minor signal being seen at 4.84 ppm. After hydrolysis, the FMN^3−^ signal almost completely disappeared and a new signal at 4.79 ppm was observed, which we ascribe to RQC^3−^. The aged sample showed two, almost equally intense signals for FMN^3−^ and RQC^3−^, confirming that the same hydrolysis product is indeed formed on aging. The minor signal seen at 4.84 ppm in the fresh sample suggests that small traces of RQC^3−^ were formed shortly after dissolution of FMN^3−^, the slight change in shift of the NMR signal being ascribed to pH changes.

To explore the electrochemical performance of RQC^3−^, the hydrolyzed sample was cycled and characterized via ^1^H NMR (Fig. 3c–e). At OCV, the ^1^H NMR spectra (Fig. 3e) showed two signals for RQC^3−^ at 7.20 ppm and 7.31 ppm, which have identical chemical shifts to the H6* and H9* signals observed in the aged sample. During charge, no plateau α_red_ (1.04 V_cell_) was observed, confirming that no FMN^3−^ is present in the solution and instead a plateau, solely from the reduction of RQC^3−^ (β*_red_) at 1.32 V_cell_ is now seen. In the corresponding ^1^H NMR spectra, the signals assigned to RQC^3−^ (H6*, H9*) gradually decreased, eventually disappearing at the end of the β*_red_ process, with the concomitant appearance of two peaks at 6.70 ppm and 6.95 ppm. The shifts of the new peaks matched those of H6*” and H9*” seen in the reduced aged sample. The process at 1.71 V_cell_ (*γ*_red_) due to water reduction was again observed, and no change in the ^1^H NMR signals were observed during this plateau, consistent with this assignment. Of note, no EPR signals were observed during either of the two plateaus β*_red_ and *γ*_red_ (Fig. S31, 32), nor did H6* and H9* disappear immediately on charging—an indication that no rapid electron transfer between diamagnetic and paramagnetic species occurs in this electrolyte mixture^3^. This further indicates that no radicals were formed during β*_red_. We, therefore, propose that RQC^3−^ undergoes a direct two-electron reduction reaction to form RQC^5−^ (Fig. 3c), resulting in the β*_red_ plateau and new ^1^H NMR signals.

On applying a negative current, the voltage dropped down to 0.5 V_cell_ without any evidence of electrochemical processes occurring. This suggested that RQC^3−^ cannot be reversibly cycled in the same manner as FMN^3−^. The cyclic voltammetry (CV) shown in the supporting information is consistent with this proposal (Fig. S30).

Further experiments revealed that RQC^5−^ could be re-oxidized by either lowering the cell voltage to below 0.5 V_cell_ (i.e., raising the potential at the anolyte) or increasing the time spent at the end of discharge at 0.5 V_cell_ (i.e., a voltage hold; Figs. S33–36; Supplementary Note 4.6), indicating that the kinetics of oxidation are much slower than those for reduction. However, both experiments showed that the completely hydrolyzed FMN can be reversibly cycled if held long enough at lower voltages, as a small fraction of the capacity was regained. We tentatively hypothesize that the difficulty of the reverse reaction is due to the structural rearrangement required to oxidize RQC^5−^ (Figs. S33–35; Supplementary Note 4.5).

### Redox mediator properties of FMN^3−^

**Fig. 4:**
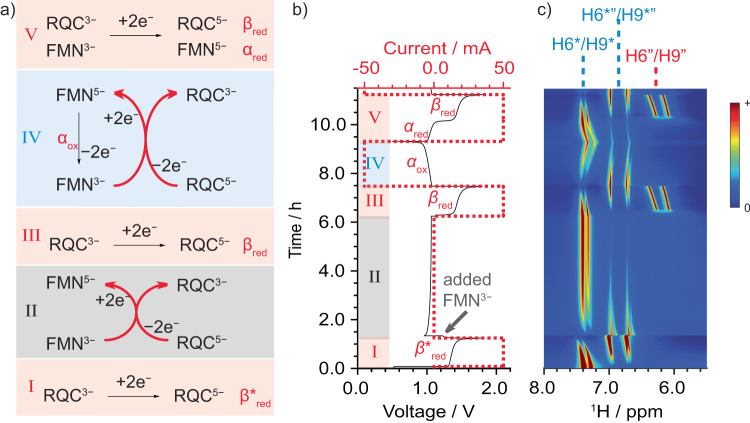
In situ NMR analysis of a mixture of fresh and hydrolyzed FMN^3−^. **a** Electrochemical and chemical processes that occur during the reaction steps, I–V: (I) Electrochemical reduction of RQC^3−^ to RQC^5−^, β*red (II) addition of FMN^3−^ and the chemical redox reaction involving FMN^3−^ as a redox mediator to form RQC^3−^ and FMN^5−^ during rest, (III) electrochemical reduction of RQC^3−^ (with FMN^5−^ remaining in solution), (IV) electrochemical oxidation of FMN^5−^, and the subsequent chemical reaction involving the oxidized product FMN^3-^ with RQC^5-^ to form RQC^3−^ and FMN^5−^, (V) electrochemical reduction of RQC^3−^ and FMN^3−^. Black arrows indicate electrochemical processes and red ones, coupled chemical reactions. **b** Voltage (black) and current (red) profiles for hydrolyzed FMN^3−^ versus 0.2 M K_4_[Fe(CN)_6_] and 0.05 M K_3_[Fe(CN)_6_] in 1 M KOH/D_2_O full cell as a function of time, while undergoing the V reaction steps shown in (I). Pink and blue shading in (**a**) and (**b**) representing charging and discharging respectively, while gray represents reactions that occur during the hold at OCV, FMN^3−^ being introduced at the beginning of this step. **c** In-situ ^1^H NMR spectra of the anolyte (RQC^3−^/RQC^5−^ and FMN^3−^/FMN^5−^) during steps I-V.

We propose that an alternative process occurs during discharge of the full cell that facilitates the re-oxidation of RQC^5−^. To determine whether the presence of FMN^3−^ plays a key role in this, a solution of RQC^3−^ (15 mL, 60 mM) was fully reduced to RQC^5−^ (Fig. 4a, b, Step I), while acquiring in situ ^1^H NMR spectra. Note that an upper cut-off voltage of 1.7 V_cell_ was used to avoid water reduction. An equal amount of fresh FMN^3−^ (15 mL, 60 mM) was then added (in Step II) and the solution was allowed to equilibrate for 5 h, the signals for RQC^5−^ slowly disappearing concomitant with an increase in the signals for RQC^3−^ in the ^1^H-NMR spectra. No signals for either FMN^3−^ or FMN^5−^ were seen throughout Step II, which is ascribed to the oxidation of RQC^5−^ by FMN^3−^, forming FMN^4−•^ and eventually FMN^5−^, and the consequent rapid electron transfer processes between the diamagnetic and paramagnetic FMN^n−^ ions. The battery was then charged (Step III), and even though FMN^3−^ had been added to the solution, plateau *α* (associated with the reduction of FMN^3−^) was not observed, and instead plateau *β*_red_ (associated with the reduction of RQC^3−^) was seen. This is supported by the in situ ^1^H NMR spectra: the appearance of the signals for RQC^5−^ was inversely correlated with the disappearance of the signals for RQC^3−^. Signals for FMN^5−^ were observed at the start of plateau *β*_red_ and their intensity remained unchanged (no signals for FMN^3−^ could be detected), confirming that an almost complete reduction of FMN^3−^ had already occurred before the start of plateau *β*_red_. This is consistent with the reduction of FMN^3−^to FMN^5−^ during the 5-hour equilibration period (step II), and the oxidation of RQC^5−^ to RQC^3−^; we propose that this occurs via an intermolecular process, in which FMN^3−^ acts as the redox mediator serving to oxidize RQC^5−^ chemically.

During discharge (step IV), the *α*_ox_ plateau was observed, but with a higher capacity (2.36 Ah L^−1^) than expected if only FMN^5−^ was oxidized to FMN^3−^ (1.61 Ah L^−1^; capacity calculated based on 30 mM FMN^3−^ as a result of dilution with 15 mL RQC^3−^ and the assumption that RQC^3−^ does not contribute towards the discharge capacity). This indicates that another species must also be oxidized during plateau *α*_ox_, again consistent with redox mediation mechanism: RQC^5−^ is oxidized by FMN^3−^ forming RQC^3−^ and FMN^5−^, which can then be electrochemically re-oxidized to FMN^3−^. However, the expected capacity of FMN^3−^ and RQC^3−^ combined (3.22 Ah L^−1^) was not observed suggesting that the redox mediator reactions are slower than the electrochemical oxidation of FMN^5−^. This is corroborated by the in situ ^1^H NMR spectra during step IV, as residual signals for RQC^5−^, in addition to those for FMN^3−^ and RQC^3−^, could be observed at the end of plateau *α*_ox_. During the following charge (step V), plateaus *α*_red_ and *β*_red_ were now observed. The same capacity was obtained as during the previous discharge cycle (step IV).

Additional experiments (Supplementary Note 5.1) probing the redox mediating effect of FMN^3−^, i.e., adding a shorter rest period (Fig. S40), an extra voltage holds (Fig S41), or less FMN^3—^containing electrolyte solution (Figs. S42, 43), confirm the mediating effect.

### Avoiding hydrolysis of FMN^3−^

**Fig. 5:**
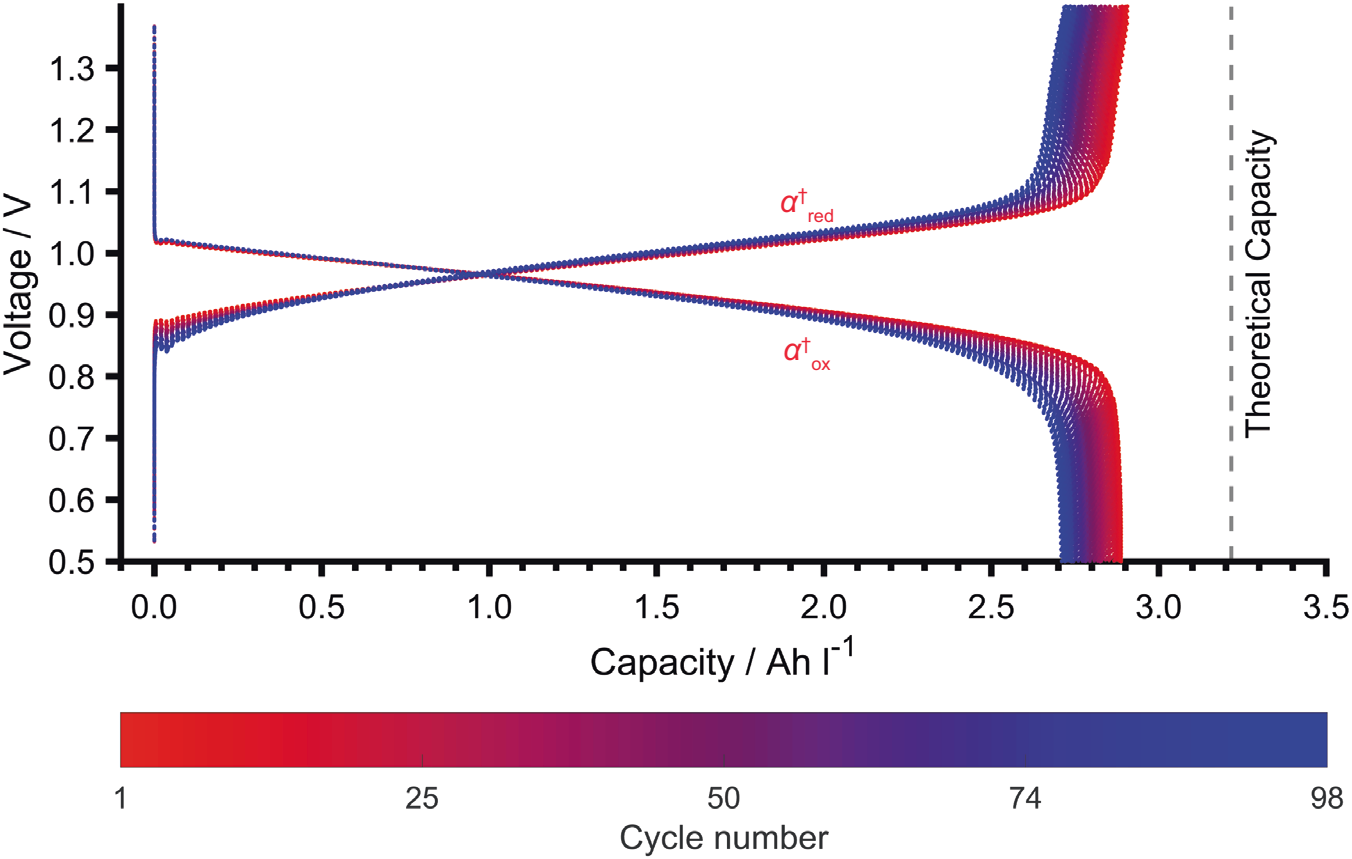
Electrochemistry of FMN^3−^ in an RFB at pH 11.4. Galvanostatic cycling of an RFB at pH 11.4 between 0.5 and 1.4 V_cell_ for 98 cycles (from cycle 2) with 0.2 M K_4_[Fe(CN)_6_] and 0.05 M K_3_[Fe(CN)_6_] in 1 M KCl/D_2_O as the catholyte (10 mL) and 60 mM FMN^3−^ in 1 M KCl/D_2_O as the anolyte (10 mL). A current density of ±10 mA cm^−2^ (±50 mA) was used over 98 cycles (full cycling performance and efficiencies in Figs. S60, 61). Two stacked membranes were used to minimize cross-over. Cycling is shown from the second cycle, the 1^st^ cycle showing additional capacity that arises from the trace amounts of oxygen present in the cell (Supplementary note 7.2, Fig. S62).

Since the hydrolysis of FMN^3−^ occurs at a high OH^−^ concentration (pH > 12) and is accelerated by higher temperatures, the pH of the electrolyte solutions was lowered to minimize hydrolysis and increase capacity retention. Supplementary note 6 provides a detailed discussion about the pH dependence and solubility of FMN (Fig. S44) and its electrochemistry at different pHs (galvanostatic cycling and CV) with and without buffers (Figs. S10, 11, 44–59). These experiments revealed that a pH of close to 11 represents the best compromise between degradation at high pH and solubility at low pH. Therefore, an anolyte solution was prepared from 60 mM FMN^3−^ and 62 mM KOH in D_2_O to obtain a pH of 11.4. 1 M potassium chloride (KCl) was added to compensate for the reduced salt concentration (previously 1 M KOH was used). 62 mM KOH was added to the catholyte (K_4_[Fe(CN)_6_]/K_3_[Fe(CN)_6_] in 1 M KCl in D_2_O) to match the salt concentration to that in the anolyte. The RFB was cycled over a narrower voltage range (0.5–1.4 V_cell_) to avoid the water splitting reaction. Over the course of 98 cycles, the electrochemical data (Fig. 5) only showed plateaus *α*^†^_red_ and *α*^†^_ox_, which are associated with the redox reaction of FMN^3−^ (Fig. 1a). No additional plateaus were observed during the initial charge cycles (unlike for the pH 14 sample, Fig. 1b). This confirms that lowering the pH below 12 prevents significant hydrolysis of FMN^3−^, leading to a noticeable improvement in capacity retention: the lifetime of this battery has been improved and the hydrolysis of FMN^3−^ was successfully avoided over the course of battery cycling along with better efficiencies (Supplementary Note 7.3). We did not observe the increase in pH seen by Nambafu et al. during cycling of their bifunctional FMN derivative, which in their case led to degradation over the course of 100 cycles^11^. Furthermore, no evidence of alloxazine hydrolysis was observed in our studies. The small loss of capacity is ascribed to crossover of the active species (Supplementary Note 7.4, Figs. S63, 64).

This work shows that the observed asymmetry in the galvanostatic profile of this battery system (and the resulting voltaic efficiency) can be explained by the presence of hydrolyzed FMN^3−^. No evidence, i.e., a second charging plateau, was seen for significant dimerization of FMN^3−^, which was previously ascribed to be the source of the second charge plateau^10^. We note that while dimerization has been seen in oxidized flavins^16^, dimerization is much less likely at higher pH due to the increased charge of the flavins and increased coulomb repulsion between these ions, which increases further on reduction. For example, since the fully reduced ion has five negative charges (FMN^5−^), dimerization would involve a species with ten negative charges.

### Improving the solubility of FMN^3−^

FMN has multiple protonation states, and its solubility is likely to be extremely pH dependent. Thus, the solubility of FMN and the effect of salt concentration was then re-evaluated at a lower pH (Supplementary Note 5, Fig S44). The solubility limit of FMN^3−^ at pH 14 (1 M KOH) was only 0.1 M^10^, but at pH 11.4, the addition of 1 M KCl to a solution of 62 mM KOH led to a much-improved FMN^3−^ solubility of 1.8 M (Fig. S65). This solubility is even higher than achieved at pH 14 with the addition of the solubilizing agent nicotinamide, where it was possible to increase the solubility of FMN^3−^ to 1.5 M. This brings the anolyte into the solubility requirements (1.0–2.0 M) required for a commercial aqueous organic RFB^17^. Furthermore, these results suggest that other simple salts may similarly help achieve higher solubility in a wider range of electrolytes. Excellent cycling performance was maintained at the lower pH of pH 11 with a higher concentration of 240 mM FMN^3−^ and a higher current density of ±50 mA cm^−2^ as shown in Fig. S66.

### Summary

**Fig. 6:**
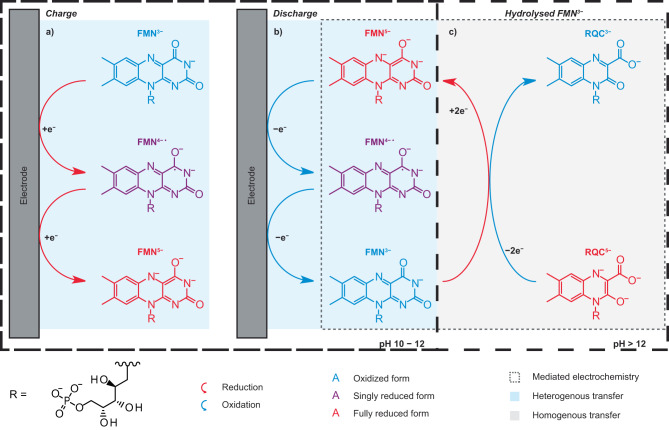
Reaction scheme of FMN^3−^ and RQC^3−^ in an RFB at pH 10–12 and pH > 12. **a** Reduction of FMN^3−^. **b** Oxidation of FMN^5−^. **c** Mediated electrochemistry of RQC^5−^ and FMN^3−^.

The use of online EPR/NMR metrologies to study degradation in an FMN (flavin mononucleotide)-based RFB is demonstrated in this work. Critically, this insight is then used to propose solutions to mitigate it. Via in- and ex-situ NMR spectroscopy, FMN^3−^ in strongly alkaline solution is shown to be chemically unstable, forming the hydrolysis product RQC^3−^, either on aging the alkaline solution over the course of four days, or on electrochemical cycling. RQC^3−^ is itself electrochemically active as shown by a new plateau at 1.42 V_cell_ (vs. Fe(CN)_6_^3−^/ Fe(CN)_6_^4−^) observed on charging solutions containing the hydrolysis products, in addition to the expected FMN reduction process at 1.02 V_cell_. The new plateau, assigned to the two-electron reduction of RQC^3−^ to form RQC^5−^, was not observed on discharge— unless deep discharging was used (i.e., a large overpotential was applied), indicating poor reversibility of the redox reaction. Despite this poor reversibility, solutions containing the hydrolysis products showed very little decrease in reversible capacity on galvanostatic cycling. Via a thorough assessment of the intermolecular processes using both NMR and EPR spectroscopy, we showed that the ability of FMN^3−^ to act as a redox mediator was responsible for this phenomenon, and in solutions containing both FMN^3−^ and its hydrolysis products, RQC^5−^ is chemically re-oxidized to reform RQC^3−^ (Fig. 6). While redox mediator processes are well established in for example lithium-air batteries^18^, their use in RFBs has been little discussed and may potentially offer new strategies for increase rates and reversibility.

In order to reduce the hysteresis in the electrochemical reaction, which reduces the overall voltaic efficiency of the reaction, we then explored approaches to reduce hydrolysis. To this end, lowering the pH to below pH 12 was found to prevent significant hydrolysis of FMN^3−^, leading to a noticeable improvement in capacity retention (Fig. 5) and both coulombic and voltaic efficiency. While, solubility is generally thought to lower the solubility of flavins, surprisingly, we found that the addition of KCl, required to maintain the salt concentration, was found to significantly improve the solubility, increasing from 0.1 M to 1.8 M on addition of KCl, leading to a higher solubility than observed in previous studies using the solubilizing agent nicotinamide^10^.

While any new chemistry requires detailed safety testing, this anolyte represents, one of the safest electrolytes/redox-active molecule currently available for flow batteries: FMN is a commercially available, non-toxic biomolecule widely used in the food industry. Moreover, the system works best at a lower pH, which also reduces the potential for corrosion, further enhancing the safety and easing the engineering requirements of the technology.

## Methods

### Materials

Flavin mononucleotide (FMN, 79% purity), potassium hexacyanoferrate(II) trihydrate (P3289, ≥98.5% purity), potassium hexacyanoferrate(III) (1049730100, ≥99.0%), ≥99.0%), and D_2_O (151882, 99.9 atom %) were purchased from Sigma Aldrich Chemicals. Potassium hydroxide (1917586, 86.4%) and potassium chloride (1858937, ≥99%) were purchased from Fisher Chemical. The aging process was conducted under air. The hydrolyzed FMN/RQC was synthesized by dissolving FMN in 1 M KOH/D_2_O and heating for 2 h at 90 °C. The solutions with a specific pH were made by dissolving a specific amount of FMN in 1 M KCl/D_2_O; the pH was then adjusted by the addition of KOH.

### Flow battery assembly

The hardware of the flow battery was purchased from Scribner Associates. Graphite flow plates with serpentine flow patterns were used for both electrodes. Each electrode comprised of three pieces of Sigracet 39 AA carbon paper (FuelCellStore) with 5 cm^2^ active area, which were used without further treatment. Nafion 212 was used as the ion transport membranes. Pre-treatment of the Nafion 212 membranes was performed by first heating the membrane in 80 °C deionized water for 20 min and then soaking it in 5% hydrogen peroxide solution for 35 min. All battery performance experiments were carried out using a potentiostat (SP-150, BioLogic SAS)^3,4^.

### Online EPR and NMR setup

The setup consists of a flow battery (Scribner), two peristaltic pumps (MasterFlex L/S 07751-20, ColeParmer), an electrochemical cycler (SP-150, BioLogic SAS), a benchtop EPR (MS5000, Magnettech), and an NMR (300 MHz, Bruker) spectrometer. The battery and the EPR spectrometer were positioned outside the 5 G line of the NMR magnet. The electrolyte was pumped through the flow battery, then flowed through the EPR and NMR magnets, and finally back to the electrolyte reservoir. The direction of flow was from the bottom to the top of both magnets. PFA tubes (1/16 in.) were used to connect the electrolyte reservoir, the battery, and the EPR and NMR sampling tubes. It took 64 s, at a flow rate of 13.6 mL min^−1^, for the electrolyte to move through the system. From the electrolyte reservoir to the battery takes 3 s, from the battery to the EPR detection region 3 s, from the EPR to the NMR detection 29 s and from the NMR back to the electrolyte reservoir is 29 s. To minimize heating of the aqueous solution by microwave irradiation, a flat EPR cell (E4503, Magnettech, *g* = 2 at 336 Gauss) was used. A customized adaptor made of polyether ether ketone (PEEK) connected the flat EPR cell to the 1/16 in. tube. The cell was orientated in the resonator such that the strength of the magnetic field was maximized, and the strength of the electric field was minimized across the sample. The volume of the cell in the excitation region of the microwave was 0.03 mL (2.00 cm × 0.50 cm × 0.03 cm), giving a residence time of 0.13 s for the electrolyte solution at a flow rate of 13.6 mL min^−1^. Details of the NMR sampling tube were provided in our previous publication^3,4^.

For the coupled in situ EPR and NMR experiment, 30 mL of 60 mM FMN was used as the anolyte, 30 mL of 150 mM K_4_[Fe(CN)_6_] and 37.5 mM K_3_[Fe(CN)_6_] was used as the catholyte. The solvent was D_2_O, with 1 M KOH as the supporting electrolyte. The flow rate was 13.6 mL min^−1^.

Pseudo-2D NMR experiments were performed by direct excitation with a 90° radio-frequency pulse. Each NMR spectrum was acquired by collecting 16 free induction decays (FIDs) with a recycle delay of 5 s. The pulse length for a 90° pulse was 27 μs. All spectra were referenced to the chemical shift of water at 4.79 ppm before battery cycling began.

For the in situ EPR measurement of FMN^4−•^ radical anions, the magnetic field was swept from 336.5 to 339 mT. The sweep time was 60 s per single scan. B_m_ was 0.001 mT, and Q was recorded for each spectrum. Q was 1306 ± 5. The temperature of the resonator was kept at 29 °C, and a time delay of 35 s was added between each scan.

### Cyclic voltammetry

CV experiments were carried out using a three-electrode cell. A Thermo Haake DC50-K75 circulator was used to ensure that the voltametric cell was at 25 °C and GL14 screw threads and caps, in combination with rubber O-rings, were used to ensure a tight seal around the electrodes. A rubber septum was used to close the remaining opening, and for addition and removal of samples under inert atmosphere. The cell was flushed with nitrogen prior to measurement and kept under an overpressure of the inert gas during measurement using the ground-glass opening at the top of the cell. The nitrogen was dried over a desiccant column before use. All voltametric experiments used a glassy carbon working electrode embedded in a PEEK housing (Biologic A-012744, 3 mm) and a coiled platinum counter electrode (1.00 mm diameter). The glassy carbon was polished using 0.05 µm polishing alumina paste (Biologic A-001050) on alumina polishing pads (Biologic A-001040), and where necessary using 1 µm polishing diamond paste (Biologic A-002054) on diamond polishing pads (A-001041) with Millipore deionized water. 3 × 100 cycles of figure-eight were used to polish the working electrode, with intermediate sonication steps of one second duration followed by drying under nitrogen gas. The platinum was purchased from Sigma-Aldrich and cleaned using nitric acid and deionized water before use. A biologic VSP 200 potentiate was used, with the potentiostat software, for the experiments described here.

Electrolyte and supporting electrolyte solutions were prepared under inert atmosphere, avoiding the use of vacuum grease where possible, prior to measurement. A background CV was run on the supporting electrolyte solution to ensure that it was sufficiently free from contamination prior to use. 20 mVs^−1^ was chosen as the comparison scan-rate, providing a compromise between duration of the experiment and sufficient time to allow relaxation and separation of the different redox processes. Cyclic voltammetry was run over long time periods where possible to investigate the stability of the analyte against the supporting electrolyte fully, and to observe if alternative detrimental processes, such as electrode deposition, occurred.

### Density functional theory calculations

The software package Gaussian 16^19^ was used for all calculations. The molecules were first optimized at the PBE^20,21^/TZVP^22,23^ level of theory without any solvation model applied. The optimized coordinates were then used as the initial structure for optimization at the PBE/TZVP level of theory with an applied polarized continuum model (PCM)^24^ of solvation using the dielectric constant of water. These optimized structures were then further optimized at the B3LYP^25–28^/TZVP level of theory, again using the PCM model for water. The energetics and vibrational frequencies were calculated for all species of interest. The absence of imaginary frequencies confirms that the obtained geometry corresponds to a ground state structure.

### Supplementary information

Supplementary Information

## Data Availability

Data underlying this paper is freely available at 10.17863/CAM.99832.

## Code availability

All code used in this work is available from the corresponding author upon reasonable request.
