# Supplementary material for: Identifying and preventing degradation in flavin mononucleotide-based redox flow batteries via NMR and EPR spectroscopy
Source: Nat Commun. 2023 Aug 25;14:5207. doi: 10.1038/s41467-023-40649-4 (PMC10457286; doi:10.1038/s41467-023-40649-4)
Supplement: Supplementary file 1 — Supplementary Information [file 41467_2023_40649_MOESM1_ESM.pdf]

Supporting Information for

Identifying and preventing degradation in flavin mononucleotide-based redox flow batteries via NMR and EPR spectroscopy

Authors: Dominic Hey[1], Rajesh B. Jethwa[1], Nadia L. Farag[1], Bernardine L. D. Rinkel[1], Evan Wenbo Zhao[1,2], Clare P. Grey[1*]

Affiliations:

[1] Yusuf Hamied Department of Chemistry, University of Cambridge, Cambridge, UK.

[2] Present address: Magnetic Resonance Research Centre, Institute for Molecules and Materials, Radboud University, Nijmegen, NL.

*Corresponding author. Email: cpg27@cam.ac.uk

Table of Contents

Supplementary Note 1: Ex-situ studies on flavin mononucleotide

**1.1**	Ex-situ one-dimensional (1D) ^{1}H NMR and EPR analysis of fresh solutions of flavin mononucleotide in strongly alkaline media (pH 14)	**4**
**1.2**	Assignments of the ^{1}H NMR spectra via two-dimensional (2D) NMR spectroscopy	**8**
**1.3**	Analysis of the ^{1}H NMR spectra of the aged solutions	**11**

Supplementary Note 2: Electrochemistry of flavin mononucleotide

**2.1**	Electrochemical cycling of flavin mononucleotide: cyclic voltammetry	**12**
**2.2**	Galvanostatic Cycling	**14**

Supplementary Note 3: In-situ studies with NMR and EPR

**3.1**	In-situ studies of flavin mononucleotide as an anolyte	**16**
**3.2**	^{1}H NMR – Fresh sample	**16**
**3.3**	^{1}H NMR – Aged sample	**19**
**3.4**	^{31}P NMR – Fresh Sample	**20**
**3.5**	^{31}P NMR – Aged Sample	**21**

Supplementary Note 4: Further in-situ studies of the hydrolyzed flavin mononucleotide as an anolyte

**4.1**	Degradation products/hydrolysis mechanisms	**23**
**4.2**	^{13}C NMR and Infrared analysis	**25**
**4.3**	Electrochemistry of hydrolyzed FMN^{3-}: CV	**31**
**4.4**	Electrochemical Cycling of RQC^{3-}	**32**
**4.5**	DFT Calculations of RQC	**33**
**4.6**	Voltage Holds	**34**

Supplementary Note 5: Redox mediating effect of FMN^{3-}

**5.1**	Redox mediating effect of FMN^{3-}	**39**

Supplementary Note 6: pH studies of flavin mononucleotide (pH 10 and 12)

6.1 Protonation states and solubility of flavin mononucleotide **43**

6.2 Lowering the pH to pH 10 **43**

6.3 Cycling a RFB at pH 10.3 **46**

6.4 In-situ pH analysis of a RFB at pH 14 and pH 10.10 **47**

6.5 In-situ ^{1}H and ^{31}P analysis of a RFB at pH 10 **53**

6.6 Running an RFB with a buffered solution **54**

Supplementary Note 7: Flavin mononucleotide RFB at pH 11 and study of oxygen side reactions

7.1 Cycling performance at pH 11.4 unbuffered **58**

7.2 Study of oxygen side reactions (pH 14) **59**

7.3 Efficiencies at pH 11.4 unbuffered **60**

7.4 Source of the minor capacity loss at pH 11.4 unbuffered **60**

7.5 Maximum solubility of flavin mononucleotide **62**

Supplementary Note 1: Ex-situ studies on flavin mononucleotide

1.1 Ex-situ one-dimensional (1D) ^{1}H NMR and EPR analysis of fresh solutions of flavin mononucleotide in strongly alkaline media (pH 14)

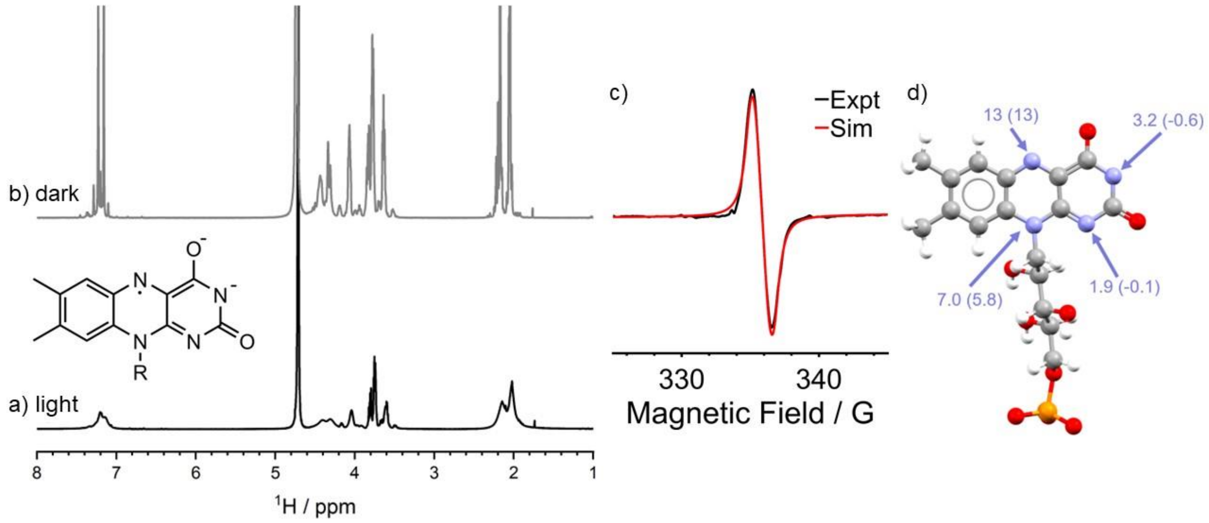

Fig. S 1: Ex-situ NMR and EPR of FMN. (a) ^{1}H NMR spectra of 60 mM FMN^{3-} in 1 M KOH/D_2O 10 minutes after preparation with light exposure. The insert shows the skeletal diagram of $FMN^{4-\bullet}$. (b) ^{1}H NMR spectra of 60 mM FMN^{3-} in 1 M KOH/D_2O 10 minutes after preparation without light exposure. (c) EPR data from a 60 mM FMN^{3-} sample in 1 M KOH/D_2O, 30 minutes after dissolution (with light exposure), plotted against the fitted model derived assuming all nitrogen atoms in the ring system are coupling to the unpaired electron. Different fits with different nitrogen-hyperfine coupling constants, performed to explore the errors, are shown in Fig. S 4. (d) DFT-derived model of $FMN^{4-\bullet}$ labelled with the experimentally obtained ^{14}N hyperfine coupling constants (in MHz). The values in brackets are the DFT derived coupling constants of the nitrogens indicated by arrows. DFT calculations were performed at the B3LYP/TZVP level of theory.

The ^{1}H NMR spectrum of FMN^{3-} on dissolution in 1 M KOH solution was first examined (Fig. S 1) and the resonances were surprisingly broad, with the spectrum appearing to contain more than one species. The broadening was attributed to the presence of paramagnetic species, which we hypothesized were generated via a photo-reduction process.

To confirm this, an EPR spectrum was acquired of the freshly dissolved 60 mM FMN^{3-} immediately after dissolution (Fig. S 2a), and after 30 minutes with light exposure (Fig. S 1c and Fig. S 2b). Comparison of the two samples indicates that the radical only forms upon exposure to light, with the exposed sample resulting in a much more intense EPR signal. The presence of a weak EPR signal in the light-excluded sample may be due to incomplete exclusion of light during sample measurement e.g., light can enter through the sample holder.

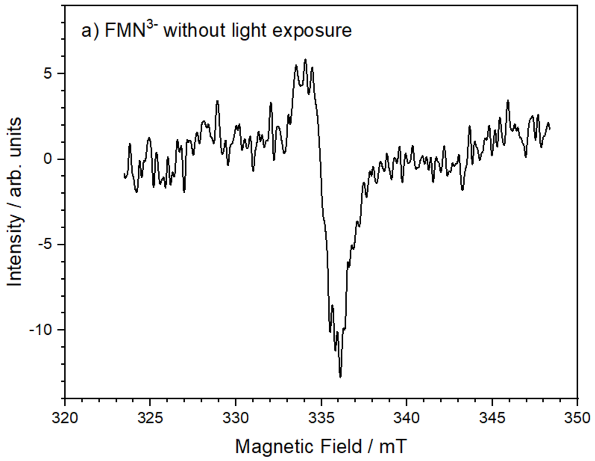

Fig. S 2: EPR signal of FMN. EPR signals of 60 mM FMN^{3-} in 1 M KOH/H2O. (a) shows the EPR spectrum of the solution measured immediately after dissolution and without light exposure and (b) shows the spectrum with light exposure 30 minutes after dissolution.

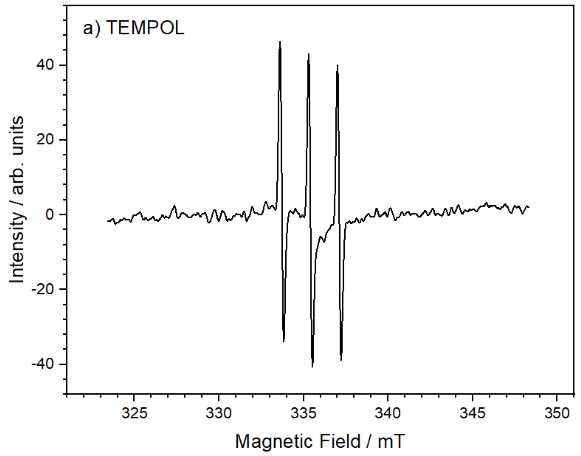

Fig. S 3: Radical concentration. EPR signals of a (a) 0.1 mM TEMPOL in 1 M KOH/H_2O and a (b) 60 mM FMN^{3-} in 1 M KOH/H_2O.

As discussed above, an EPR signal is seen when the solution is exposed to light, which is assigned to $FMN^{4-\bullet}$. The signal was observed to increase by an order of magnitude over 30 minutes of continuous light exposure (Fig. S 3). To obtain an approximation of the radical concentration of $FMN^{4-\bullet}$, a 100 mM solution of 4-hydroxy-2,2,6,6-tetramethypiperidin-1-oxyl (TEMPOL) in 1 M KOH/H_2O was diluted with supporting electrolyte to a concentration of 0.1 mM, where the intensity of the TEMPOL signal approximately matched the intensity of the $FMN^{4-\bullet}$ signal (Fig. S 3a). By comparing the areas of the double integrals of the $FMN^{4-\bullet}$ and TEMPOL signals, and using the TEMPOL concentration, the concentration of $FMN^{4-\bullet}$ was estimated to be approximately 3 mM (5% of the total amount of FMN^{3-} in the solution).

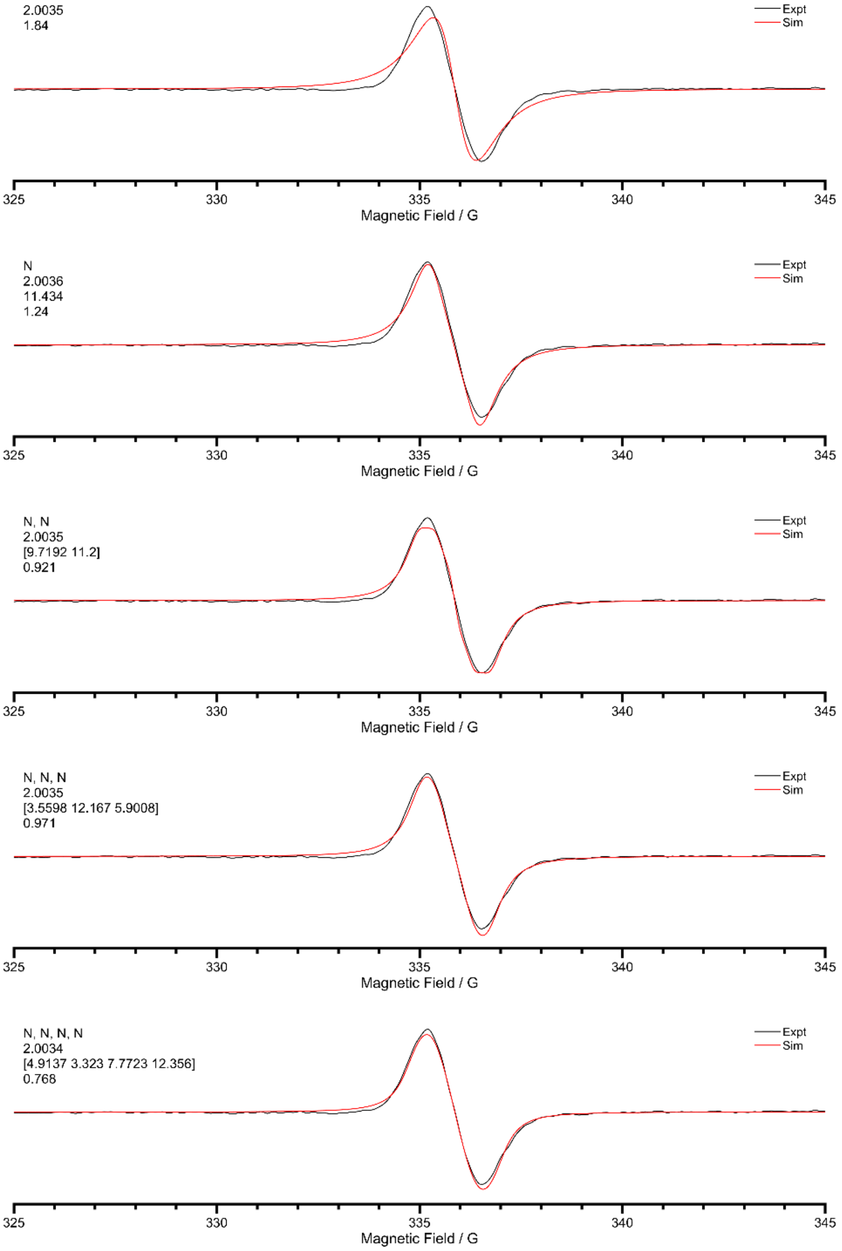

Fig. S 4. Fitting of EPR signal. Fitting of the EPR signal using different numbers of nitrogen atoms and hyperfine coupling constants. The EPR signal was from a 60 mM solution of FMN^{3-} in 1 M KOH/H_2O. The first graph shows the fit assuming no hyperfine coupling, the second with the radical coupling to one nitrogen atom, the third coupling to two nitrogen atoms, the fourth with three nitrogen atoms and the fifth with four nitrogen atoms. The black line shows the experimental data and the red line shows the simulated spectrum. The legend on the left side of each figure shows (from top to bottom): how many nitrogen atoms are used to fit the experimental data, the g factor of the signal, the hyperfine coupling constants of each nitrogen (in square brackets) and linewidths.

Through simulation of the EPR spectra of FMN$^{4-•}$ with EasySpin 5.2.35 (Open-source MATLAB toolbox) (Fig. S 1c, Fig. S 4) a g-factor of 2.0034 was calculated. Interactions with multiple nitrogen atoms were

considered, with variation from one to four atoms. While the fit was reasonable with a single nitrogen, a good fitting was achieved when all four N were included.

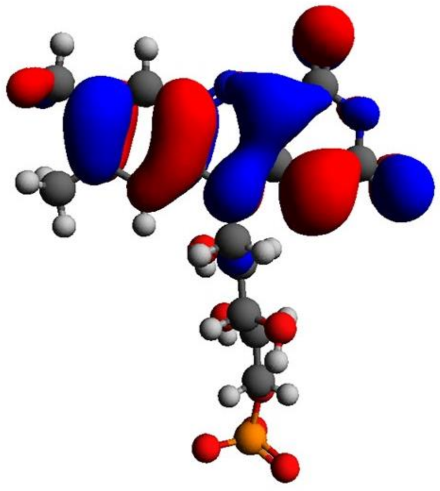

Fig. S 5: DFT calculations of FMN. Singly occupied molecular orbital of $FMN^{4-\bullet}$ as calculated by DFT.

Density functional theory (DFT) calculations were used to support the EPR analysis. Hyperfine coupling constants of 13 MHz, 7 MHz, 3 MHz, and 2 MHz for all four nitrogen atoms were estimated (Fig. S 1d, Fig. S 5) and aided in the assignment of hyperfine coupling constants to specific N atoms. Calculations of the singly occupied molecular orbitals (SOMO) were performed using the Gaussian 16 code. The spin polarized B3LYP/TVZP functional was used in combination with a polarized continuum model for implicit aqueous solvation for structural relaxation. These calculations agree with previous determinations of the isotropic coupling constants of nitrogen aromatic radicals, i.e., 2,2-diphenyl-1-picrylhydrazyl, a stable free radical molecule, which has a calculated coupling constant of 10.5 MHz with B3LYP/TZVP vs. an experimental coupling constant of 9.74 MHz [29].

The broadening seen in the NMR spectrum (Fig. S 1) of the freshly dissolved FMN^{3-} anion, given the relatively low radical concentration, is attributed to electron exchange between the diamagnetic and paramagnetic flavin anions with a correlation time that is on the same order of timescale as the proton hyperfine interactions. A similar phenomenon was previously seen in partially reduced samples of quinone anions and results in broadened NMR spectra and the disappearance of resonances at high radical concentrations, particularly the proton signals associated with large hyperfine interactions [2,3]. To indirectly confirm the presence of $FMN^{4-\bullet}$ in the solution, either oxygen or nitrogen were separately bubbled through the solution for five minutes. After bubbling oxygen (Fig. S 6a), considerable sharpening of the resonances was observed, the experiment proving that in the presence of oxygen, the semiquinone $FMN^{4-\bullet}$ can be re-oxidized back to FMN^{3-}. Nitrogen was used as a control to account for any other effects that may affect the NMR signals, (Fig. S 6b), the resonances remaining broad after flushing with nitrogen Thus, the effects of the photo-reduction of FMN can be mitigated by either flushing with oxygen gas (Fig. S 6) or by preparing a sample in the dark (Fig. S 1b); these results also indicate that small traces of oxygen will reduce any $FMN^{4-\bullet}$ formed electrochemically.

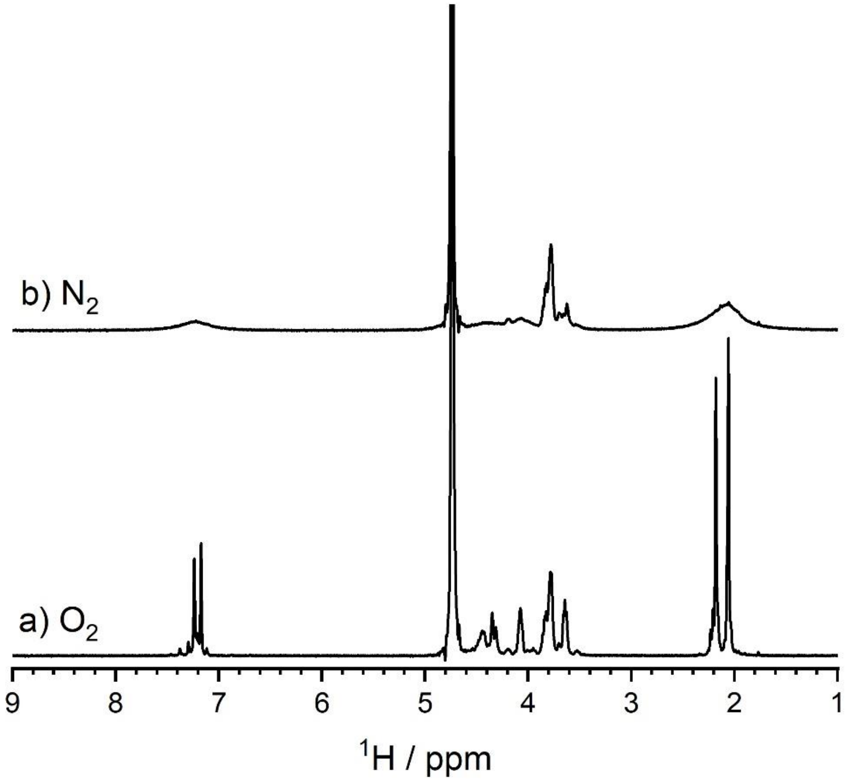

Fig. S 6: Avoiding broadening of 1H-NMR resonances. ^{1}H-NMR spectra of 60 mM FMN^{3-} in 1 M KOH/D_2O on the day of preparation. a) Oxygen O_2 was bubbled through the solution for five minutes. b) Nitrogen N_2 was bubbled through the solution for five minutes.

1.2 Assignments of the ^{1}H NMR spectra via two-dimensional (2D) NMR spectroscopy

The FMN sample prepared in the dark (Fig. S 2a) was also analyzed by 2D ^{1}H-^{1}H correlation spectroscopy (COSY) spectroscopy (Fig. S 7, Fig. S 8). Off-diagonal peaks (or cross peaks) in a ^{1}H-^{1}H NMR COSY spectrum indicate coupling between protons that are usually three, or occasionally four bonds away. The ^{1}H-^{1}H COSY NMR spectrum (Fig. S 7) showed correlations between the ^{1}H NMR signals at 2.03 ppm (H7α) and 2.16 ppm (H8α), and at 7.15 ppm (H6) and 7.21 ppm (H9), indicating that the ^{1}H environments giving rise to these signals are connected through chemical bonds. Additionally, the ^{1}H NMR signals at 3.61 ppm (H2') and 3.75 ppm (H1'), 3.61 ppm (H2') and 4.04 ppm (H3'), 4.04 ppm (H3') and 4.41 ppm (H4'), and 4.31 ppm (H5') and 4.41 ppm (H5') show correlations to each other, confirming the assignments of the ^{1}H resonances to the specific locations shown in the figures.

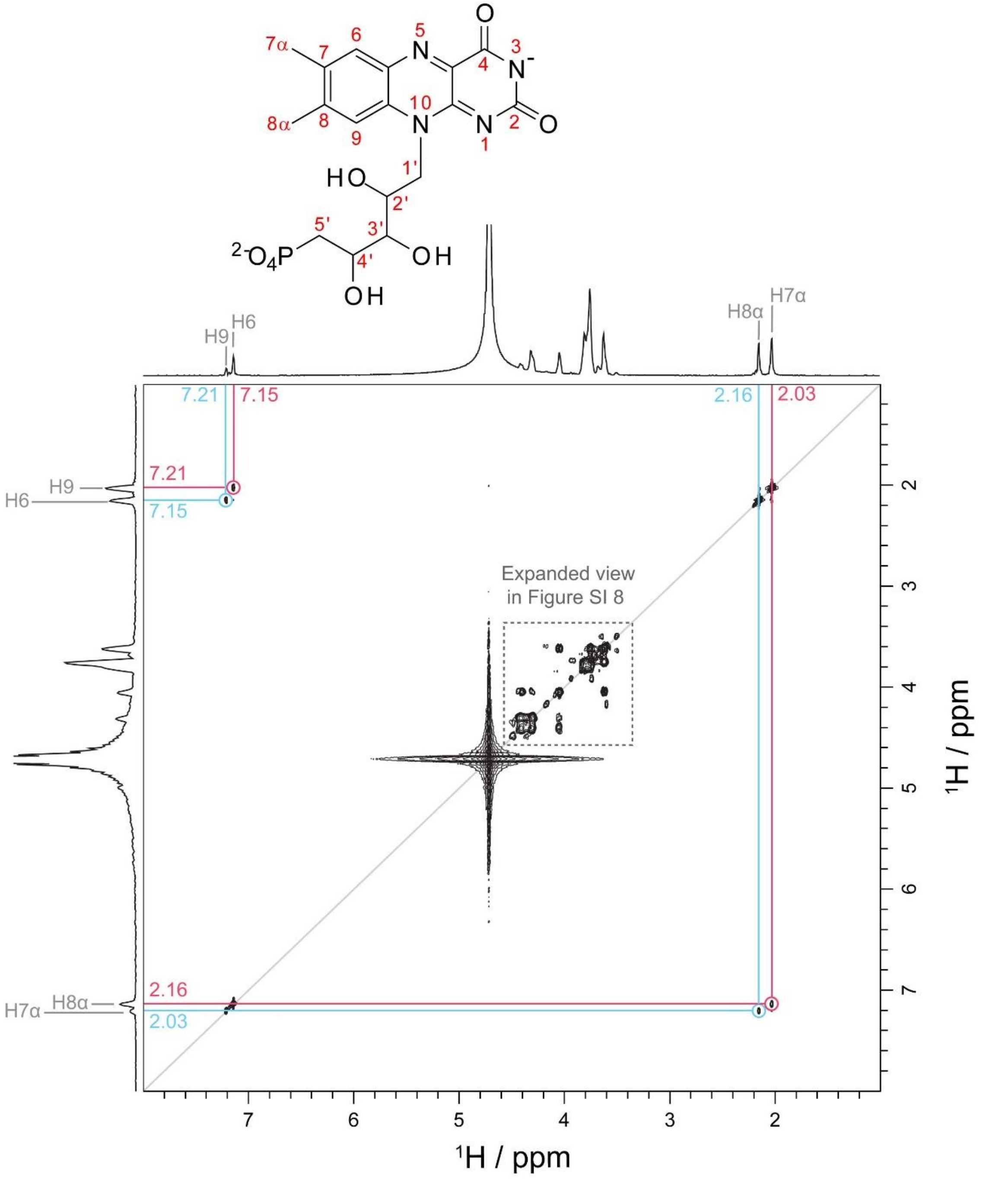

Fig. S 7: COSY of FMN. ^{1}H-^{1}H COSY NMR spectrum (9 to 1 ppm range) of 60 mM FMN^{3-} in 1 M KOH/D_2O. The sample was prepared in the dark. Labeling of the proton positions is shown above the figure.

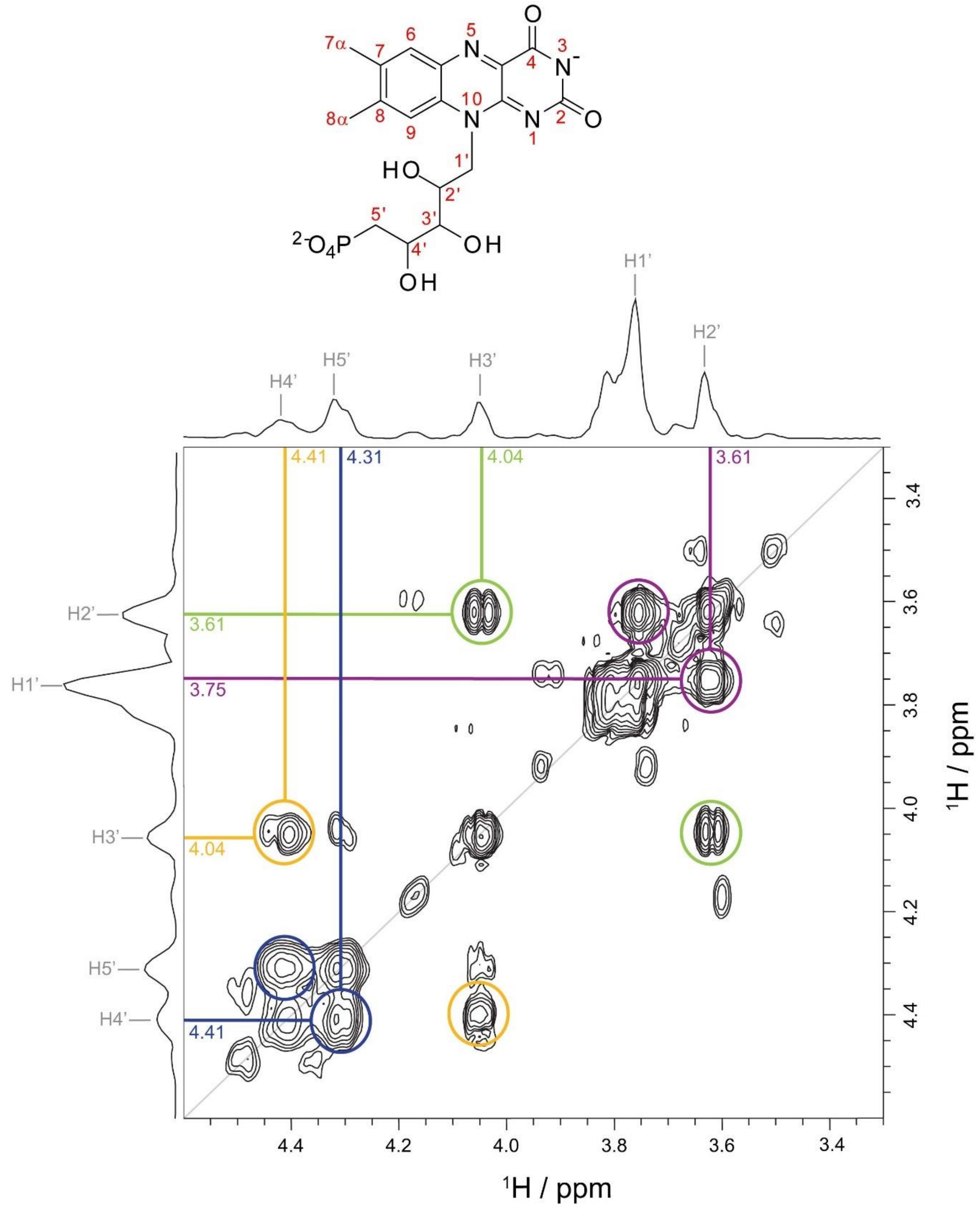

Fig. S 8: Enlarged COSY of FMN. Enlargement (showing 4.6 ppm to 3.3 ppm range) of the ^1H-^1H COSY NMR spectrum shown in Figure S7 of 60 mM FMN^{3-} in 1 M KOH/D_2O. Labeling of the proton positions is shown above the figure.

1.3 Analysis of the ^{1}H NMR spectra of the aged solutions

When considering the spectra of the light-exposed sample (Fig. S 1, Fig. S 9) with respect to the spectral assignments above, it is clear that it is the resonances from the isoalloxazine ring protons (H6: 7.54 ppm; H9: 7.71 ppm) and the nearby methyl carbons (H7α: 2.31 ppm; H8α: 2.45 ppm) broaden. These effects are consistent with electron-transfer between $FMN^{4-\bullet}$ and FMN^{3-}. The protons near the phosphate group are also broadened. This is tentatively explained by changes in the pKa and thus degree of proton exchange of these environments as a result of this exchange process.

For the light-exposed sample, the NMR signals were observed to sharpen over time (Fig. S 9). This is attributed, in part, to gradual oxygen contamination resulting in oxidation of any $FMN^{4\bullet-}$ radicals present in the solution. New peaks were observed to develop over the course of four days, which could not be assigned to the FMN^{3-} anion (most notably doublets centered at 6.47 ppm and 6.83 ppm). This suggests that FMN^{3-} undergoes chemical degradation over time. Based on the chemical shifts, however, the degradation product is likely to be structurally related to FMN^{3-}. Therefore, from this preliminary analysis it is clear that multiple potential side reactions must be considered when analyzing the performance of this system – a photo-redox process, redox reactions with trace oxygen, and degradation of FMN^{3-} over time at high pH.

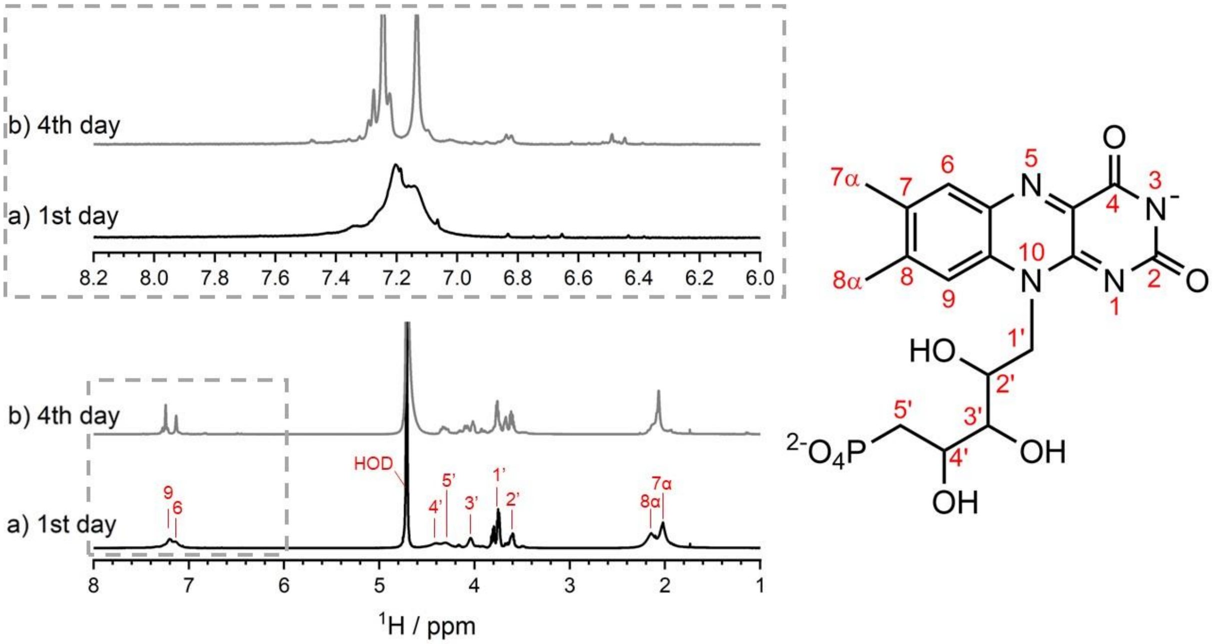

Fig. S 9: ^{1}H NMR spectra of aged FMN. (left) ^{1}H NMR spectra of 60 mM FMN^{3-} in 1 M KOH/D_2O 10 minutes after preparation (a) and four days after preparation (b). The full spectra (1-8 ppm) can be observed at the bottom, whereas the top spectra provide an expanded view (6 to 8.2 ppm, represented by the grey dashed box in the bottom two spectra). (right) Skeletal structure of FMN^{3-}, i.e., the protonated state expected when in strongly alkaline solution (pD > 12).

Supplementary Note 2: Electrochemistry of flavin mononucleotide

2.1 Electrochemical cycling of flavin mononucleotide: cyclic voltammetry

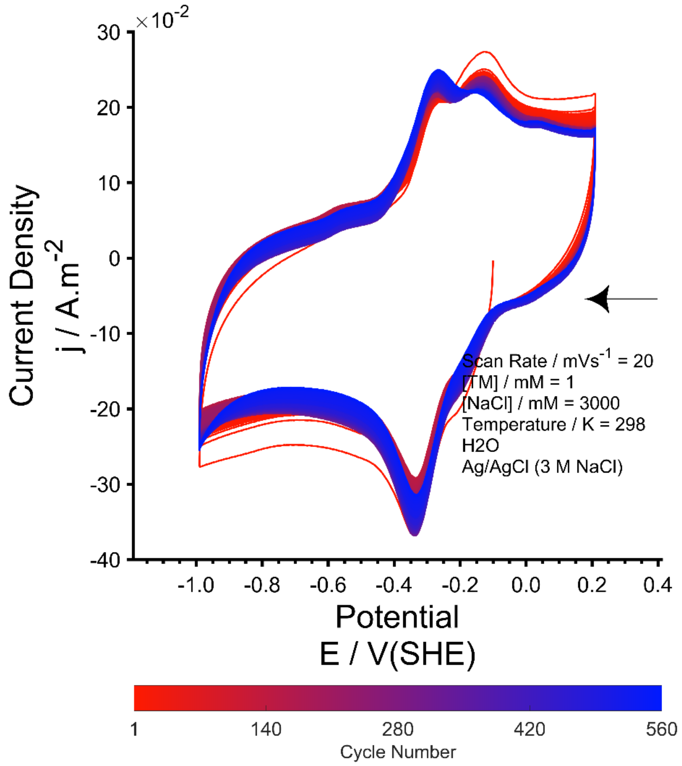

Fig. S 10: CV of FMN at neutral pH. The evolution of the CV of 1 mM FMN in 3 M NaCl in degassed D_2O, vs. the Ag/AgCl couple over the course of 12 hours (560 cycles).

Fig. S 10 displays the long-term cyclic voltammetry (CV) of a 1 mM solution of FMN with 3 M sodium chloride (NaCl) in D_2O. At near neutral pH, two peaks (−0.26 V and −0.11 V versus SHE) were observed during oxidation, and one intense (−0.34 V versus SHE) and one weak peak (−0.17 V versus SHE) during reduction. The two major peaks observed in the reductive and oxidative waves are indicative of a two-step redox mechanism, with one process being more reversible than the other. The two-step electrochemical process suggests that an intermediate radical is generated as FMN is reduced (Figure 1a). CVs were collected repeatedly over a period of 12 h during which no evolution in the electrochemical processes was observed.

Long-term (48 h) CV on a 1 mM solution of FMN dissolved with 1 M KOH in degassed D_2O gave the results presented in Fig. S 11. The first cycle of the solution shows one peak during both the reductive and oxidative waves at −0.50 V and −0.70 V versus SHE, respectively. The shift at high pH of the redox potential is shifted towards negative potentials compared to the potential observed in neutral solution is typically ascribed to an increase in electron-donation to the redox active site, making the system harder to reduce. In this case, this increased electron density may be due to the larger negative charge of the anion in alkaline solution (FMN^{3-}).

The electrochemistry (Figure 1a) and NMR/EPR shown in the main text is consistent with a two-electron redox reaction mechanism of FMN in strongly alkaline solution, involving a radical intermediate. Although only one process is seen, true multi-electron transfers are rare[30], and, hence, the observed

voltammogram is likely to be the superposition of two electrochemical processes with very similar potentials.

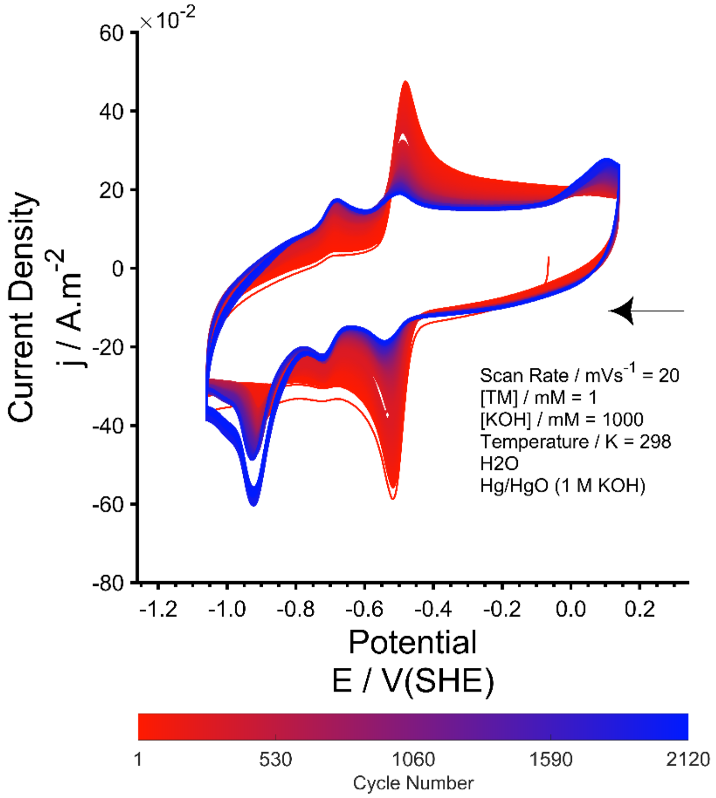

Fig. S 11: CV of FMN at pH 14. Long-time (48 h) CV of 1 mM FMN in 1 M KOH in degassed D_2O.

In contrast to the CV performed in neutral solution, a significant change over 48 h is seen in the strongly alkaline solution. The initial redox couple centered at −0.50 V versus SHE decreased in intensity while another redox couple at more negative potentials (-0.70 V versus SHE) increased in intensity. Additionally, another reduction peak was observed to form over time at −0.91 V. These signals are likely to arise from degradation products of FMN.

2.2 Galvanostatic Cycling

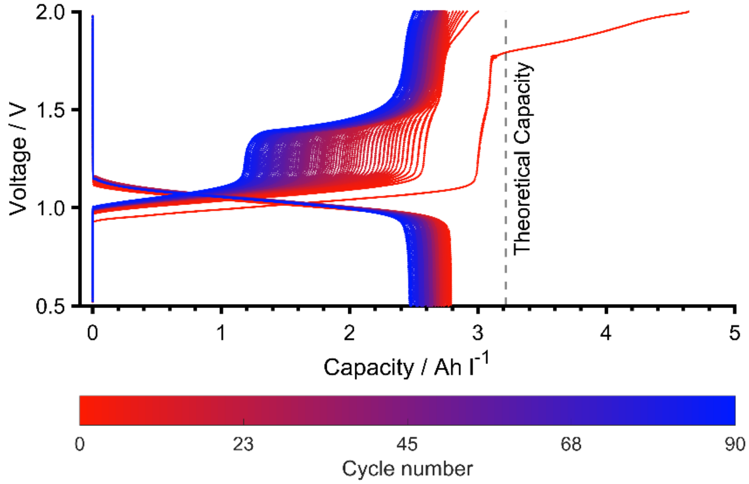

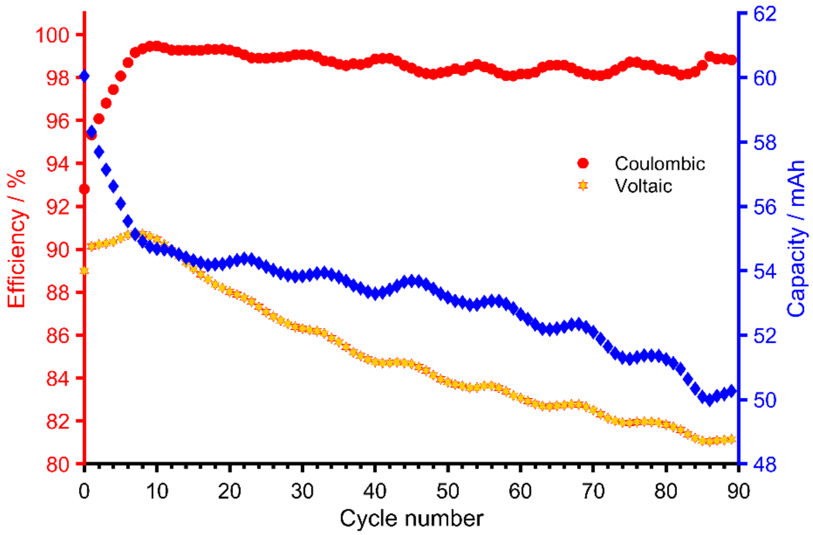

Fig. S 12: Galvanostatic cycling of FMN. Cycling a RFB between 0.5 V and 2.0 V for 90 cycles with 0.2 M $K_4[Fe(CN)_6]$ and 0.05 M $K_3[Fe(CN)_6]$ in 1 M KOH in D_2O as a positive electrolyte and 60 mM FMN 1 M KOH in D_2O as a negative electrolyte. The top figure shows the charge-discharge profiles at a current density of 10 mA cm^{-2} (corresponding to a current of ±50 mA for the cell) over 90 cycles. The bottom figure shows the cycling discharge capacity and efficiencies of the RFB system.

The electrochemistry of flavin mononucleotide in 1 M KOH/D_2O has been described in the main text (Fig. 1c) and is reproduced here in Fig. S 12 with the Coulombic and Voltaic efficiencies. The chemical used in this work has a purity level of around 79% (as determined by the vendor), the main impurity being riboflavin. Riboflavin is very closely related to FMN having a hydroxy group in place of the phosphate group, however, it has significantly lower solubility compared to FMN. The electrochemistry of riboflavin is likely to be very similar to that of FMN. The theoretical capacity marked in Figure S12 was calculated assuming 100% purity of the FMN. If the capacity was calculated based on 79% purity, then it is possible

that experimental capacity may be higher than the theoretical capacity, additional capacity coming from the Riboflavin. Thus, this assumption provides another reason why the measured capacity is lower than that predicted theoretically. Other impurities of this chemical are not well determined and may not be electrochemically active. Note we do not see evidence of any other major electrochemical processes in the voltage window investigated, other than those noted.

The coulombic and voltaic efficiencies increase until the 10 th cycle (Fig. S 12), as the extent of the process at > 1.71V, assigned to a hydrogen evolution reaction, decreases; the loss of this process is largely responsible for the noticeable drop in capacity in the 1st 10 cycles. After the 10th cycle, the capacity drops more steadily from 54.3 mAh to 50.2 mAh after 90 cycles (Fig S 12). A noticeable drop in the voltaic efficiency from 90.4 to almost 81.0 % is also seen, largely caused by the large voltage hysteresis between charge and discharge. This is ascribed to the different reactions seen on oxidation vs. reduction. After the 10th cycle, the charge reaction increasingly involves the direct reduction of RQC^{3-}, while the reverse reaction involves the oxidation of RQC^{5-} *via* FMN as a redox-mediator (see main text).

We note that the efficiencies and the discharge capacities were observed to undergo a periodic fluctuation over a 24 h interval. Flavin molecules are photo-sensitive (as confirmed by the EPR/NMR experiments described above), so it is likely that illumination of the solution also have a significant influence on the cycling performance of this battery set-up. Diurnal temperature fluctuations cannot be entirely excluded as temperature was not recorded during this experiment.

An excess of $K_4[Fe(CN)_6]$ was used as the catholyte to ensure that FMN^{3-} was the capacity limiting species and to account for potential side reactions during charging. Additional $K_3[Fe(CN)_6]$ was also added to the catholyte tank to account for potential side reactions during discharge.

Supplementary Note 3: In-situ studies with NMR and EPR

3.1 In-situ studies of flavin mononucleotide as an anolyte

A lab-scale flow battery was positioned outside the EPR and NMR magnet (Fig. S 13). The electrolyte solution was pumped consecutively from the reservoir to the electrodes, then sequentially through a flow EPR tube and an NMR tube, and back to the electrolyte reservoir with a round-trip time of 64 s. The residence time in the EPR detection region was 0.13 s, which is much longer than the electron spin-lattice relaxation time that is typically on the timescale of microseconds for semiquinones. The residence time inside the NMR magnet was approximately 20 s.

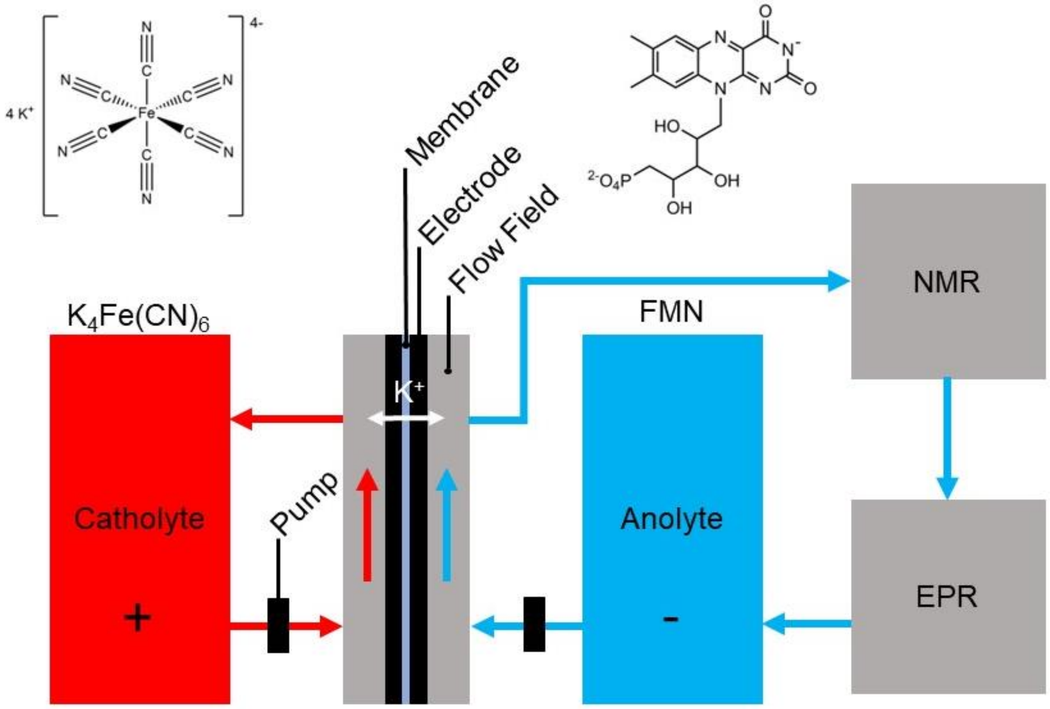

Fig. S 13: ***in-situ*** **set up.** The flow battery comprising two peristaltic pumps, a benchtop EPR and a 300 MHz NMR spectrometer. The direction of flow is indicated by arrows. The battery and the EPR spectrometer are positioned outside the 5 Gauss line of the NMR magnet.

Note that the resolution of the spectra collected with this set-up is different and typically worse than ex-situ analysis due to the convective motion of the liquid; this causes the spectra of the samples to look different from the previous ex-situ measurements (Fig. S 1, Fig. S 9).

3.2 ^{1}H NMR – Fresh sample

Fig. S 14 shows the first charge-discharge cycle of a full cell containing freshly prepared FMN^{3-} (60 mM in 1 M KOH/D_2O) electrolyte correlated with the in-situ ^{1}H NMR and EPR data. At the start of the experiment, while no current is applied to the system, the ring protons H6 and H9 as well as the methyl protons H7α and H8α are clearly seen in the ^{1}H NMR spectrum. Additionally, the EPR spectra does not show any evidence of $FMN^{4-\bullet}$ as it is a freshly prepared sample that has not been exposed to light long enough for photo-reduction to occur.

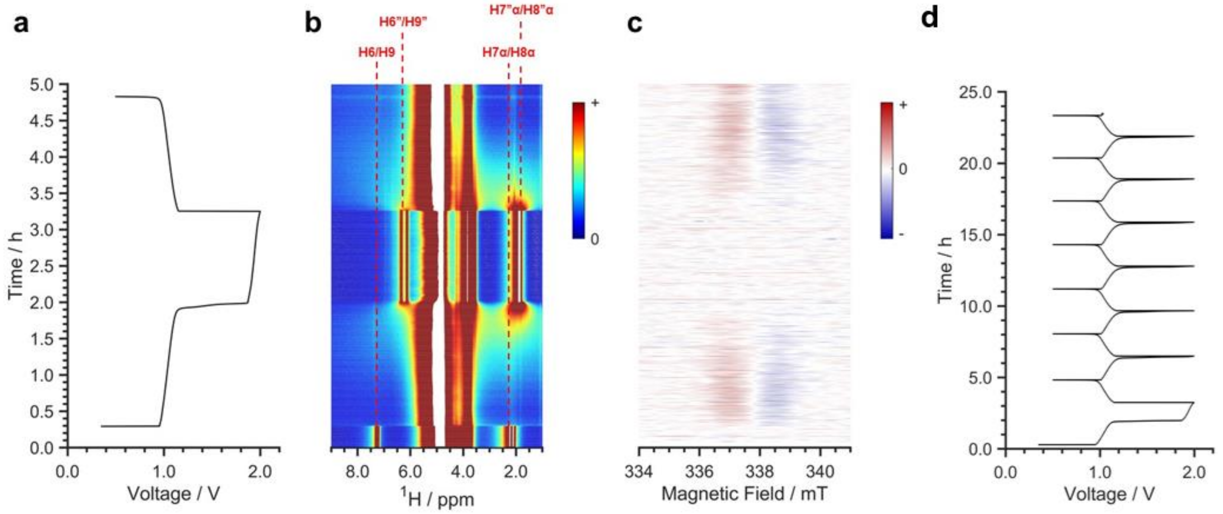

Fig. S 14: Analysis of fresh FMN. a) Voltage of a freshly prepared 60 mM solution of FMN^{3-} versus 0.2 M $K_4[Fe(CN)_6]$ and 0.05 M $K_3[Fe(CN)_6]$ in 1 M KOH/D_2O full cell as a function of time. During charge, a constant current density of 10 mA cm^{-2} (50 mA) was applied until 2.0 V was reached. During discharge, a constant current density of −10 mA cm^{-2} (−50 mA) was applied until 0.5 V was reached. (b) ^{1}H NMR spectra of the anolyte. The color bar indicates the intensity of resonance in positive arbitrary units. The acquisition time per NMR spectrum was 40 s. (c) EPR spectra of the anolyte. The acquisition time per EPR spectrum was 95 s, with a scanning time of 60 s, a coupling time of 30 s and a delay time of 5 s. The color bar indicates the intensity of the resonance in arbitrary units. Note that a different color scale was applied to the EPR spectra because of the presence of negative peak intensities. d) Full electrochemistry from this study.

Under the application of a current, the voltage increased to 910 mV from the OCV and the isoalloxazine ring protons (H6 and H9) disappeared almost immediately. The H7α and H8α methyl protons similarly suffered a reduction in their ^{1}H NMR signal intensity (Fig. S 14b). The ^{1}H signals furthest from the conjugated ring system broadened less, i.e., H4' and H5', the degree of broadening being related to the proximity of the protons to the unpaired spin density delocalized across the isoalloxazine ring. As the NMR signals disappeared a resonance was observed in the EPR spectra (Fig. S 14c) at 337.8 mT. The change in signal intensity in the EPR spectra correlates with the change in line broadening of the H7α and H8α ^{1}H signals throughout the first charging plateau, where increased intensity is associated with increased line broadening. However, as the EPR signal of $FMN^{4-\bullet}$ was weak throughout cycling, with a low signal-to-noise, a reliable estimation of the radical concentration could not be obtained.

During the plateau at 1.7 V, the aromatic (H6" and H9") and methyl (H7"α and H8"α) ^{1}H signals from the fully reduced form of FMN^{3-}, i.e., FMN^{5-}, appeared. No radical species were observed in the EPR spectra during this process, further suggesting that complete reduction of FMN^{3-} to FMN^{5-} had occurred. As the in-situ data confirmed the presence of FMN^{5-}, and gas bubbles were observed in the tubing (Fig. S 15), the high-voltage process was attributed to water splitting.

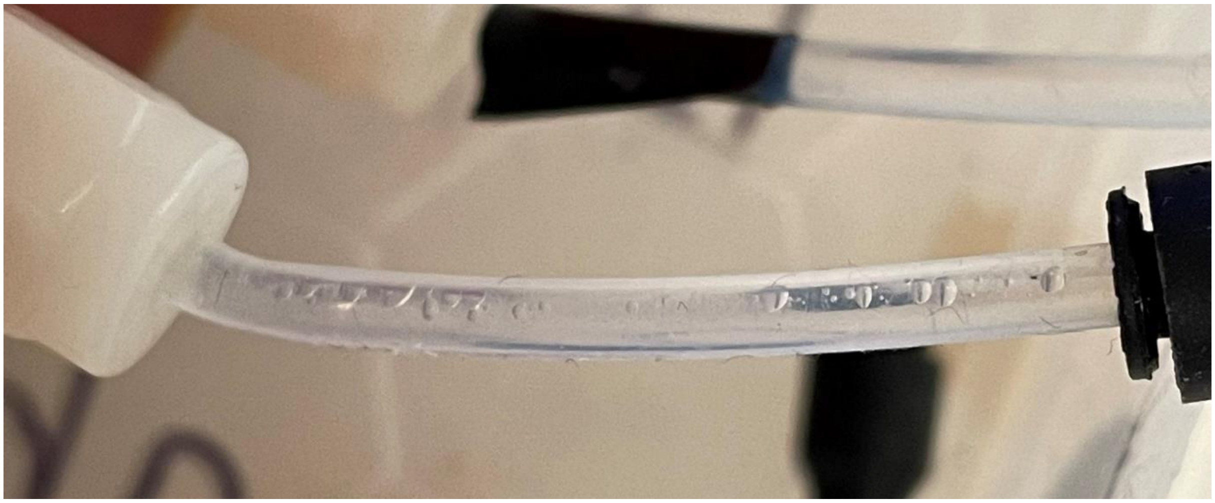

Fig. S 15: HER at 1.7 V. Tubing with gas bubbles during the charging plateau at 1.7 V.

During discharge, signals H6”, H9”, H7”α, and H8"α, were again broadened by the presence of $FMN^{4-\bullet}$ and the reverse process to that observed during charge was seen in both the NMR and EPR spectra during the 1.0 V plateau.

A g-factor of 2.0033 was obtained from fits of the EPR signals (Fig. S 16) in the middle of the charge and discharge plateaus. The g-factor did not change significantly from the ex-situ analysis, however, the fit to the in-situ data is less accurate overall due to the lower signal-to-noise ratio.

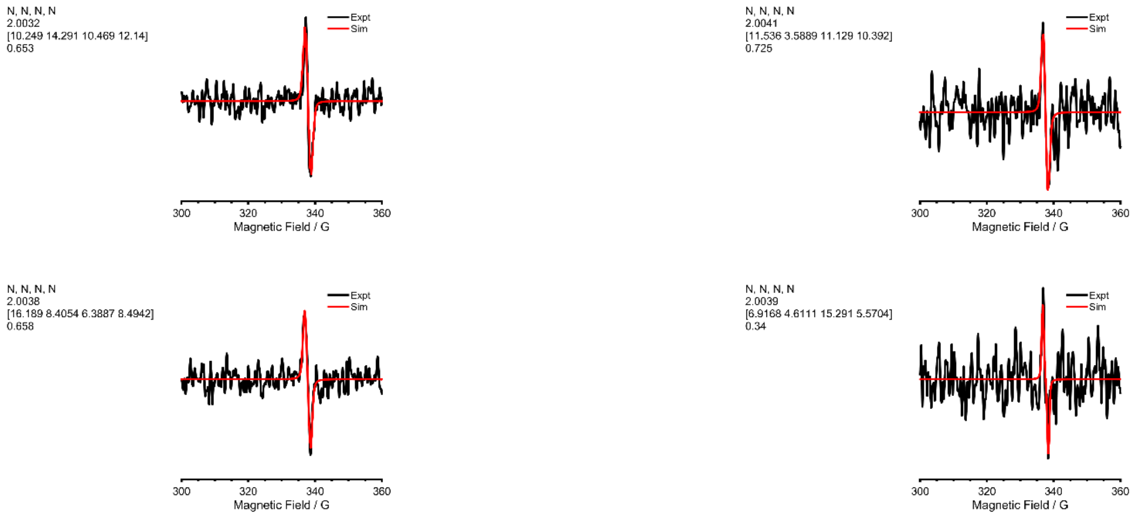

Fig. S 16: Fitting EPR singals during cycling. Fitted EPR signals. Top right: middle of charge cycle of fresh FMN^{3-} sample; top left: middle of discharge cycle of fresh FMN^{3-} sample; bottom right: middle of charge cycle of ages FMN^{3-} sample; bottom left: middle of discharge cycle of aged FMN^{3-} sample.

3.3 ^{1}H NMR – Aged sample

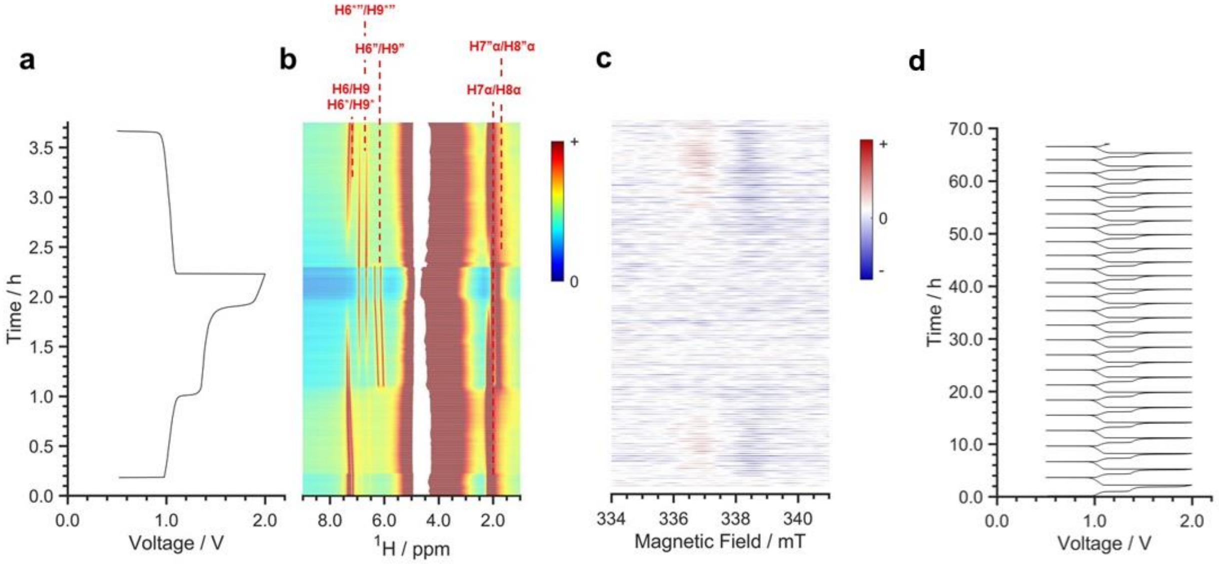

Fig. S 17: Analysis of aged FMN. (a) Voltage profile of an aged (4 days) 60 mM FMN^{3-} solution versus 0.2 M $K_4[Fe(CN)_6]$ and 0.05 M $K_3[Fe(CN)_6]$ in 1 M KOH/D_2O full cell as a function of time. During charge, a constant current density of 10 mA cm^{-2} (50 mA) was applied until 2.0 V was reached. During discharge, a constant current density of -10 mA cm^{-2} (−50 mA) was applied until 0.5 V was reached. (b) ^{1}H NMR spectra of the anolyte. the color bar indicates the relative intensity in positive arbitrary units. The acquisition time per NMR spectrum was 40 s. A plot of extracted ^{1}H NMR spectra at specific points during cycling is shown in Figure S 18. (c) EPR spectra of the anolyte. The acquisition time per EPR spectrum was 95 s, with a scanning time of 60 s, a coupling time of 30 s and a delay time of 5 s. The color bar indicates the intensity of the resonance in arbitrary units. Note that a different color scale was applied because of the presence of negative peak intensities. d) Full electrochemistry of this study.

Fig. S 17 and Fig. S 18 show the in-situ NMR and EPR spectra for the four-day aged FMN^{3-} (60 mM in 1 M KOH/D_2O) battery system. The ^{1}H signals assigned to H6 and H9 disappear almost immediately during the first charge plateau (as seen for the freshly prepared sample). The H7α and H8α methyl signals behave similarly to those from the aromatic protons (H6, H9, H6*, H9*), as described in the main text, but the broadening effect on these protons is less pronounced due to the greater distance of these nuclei from the unpaired electrons.

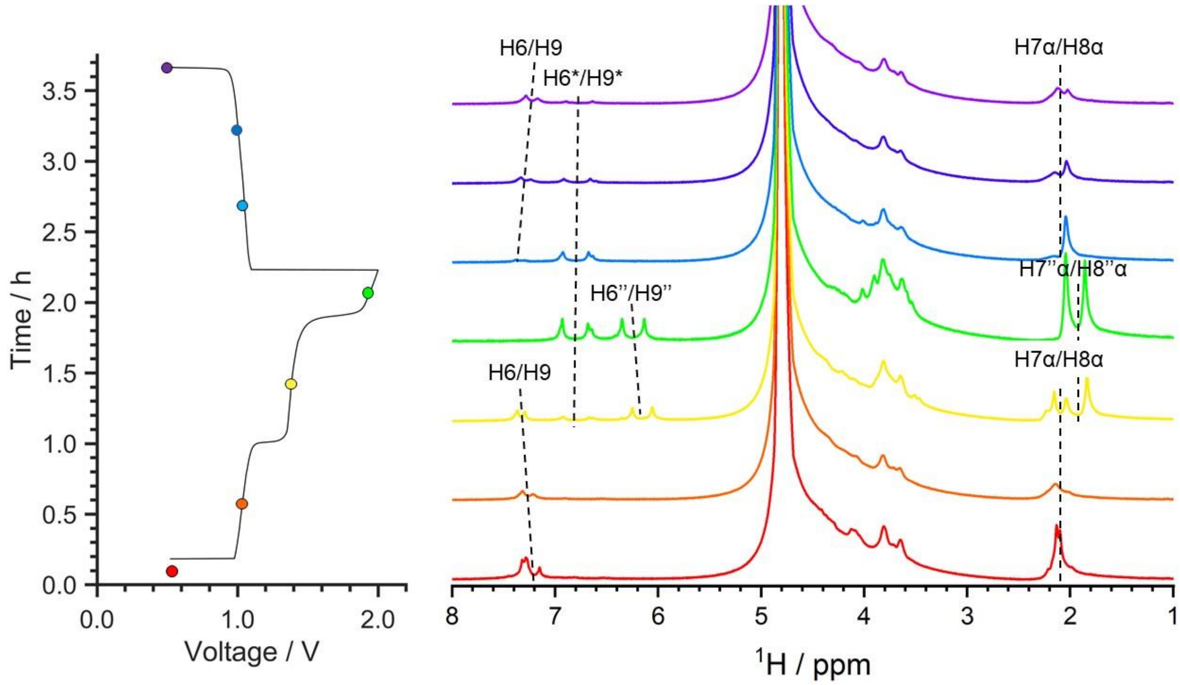

Fig. S 18: Extracted ^{1}H NMR spectra of Analysis of aged FMN. Extracted ^{1}H NMR spectra from the in-situ NMR experiment in Fig. S 17 correlated with the corresponding time during the cycling process.

<u>3.4 ^{31}P NMR – Fresh Sample</u>

In addition to the in-situ ^{1}H NMR, we also performed in-situ ^{31}P NMR experiments on both samples (Fig. S 19, Fig. S20) to identify how the signal pertaining to the phosphate group changes in chemical shift during battery cycling. Before the application of current on the fresh sample (Fig. S 19**Fig. S**), one major signal at 4.74 ppm was observed next to multiple smaller signals between 4.40 and 4.60 ppm, and one small signal at 5.30 ppm. We assign the smaller signals stem from impurities in the chemical, i.e., riboflavin diphosphate or free phosphate.

Under application of a charging current, the ^{31}P peak shifts to a higher chemical shift (4.83 ppm) and broadens significantly. This trend is consistently observed throughout the first charging plateau. The smaller signals between 4.40 and 4.60 ppm disappear during this charging plateau, whereas the signal at 5.30 ppm becomes more intense. During the electrochemical process at 1.7 V, the major signal once again sharpens, the smaller signals also reappear and the signal at 5.30 ppm becomes less intense. During discharge, the same behavior was observed. The signal at 5.30 ppm disappears during charging and discharging potentially due to radical formation as explained for Fig. S 1 and Fig. S 9.

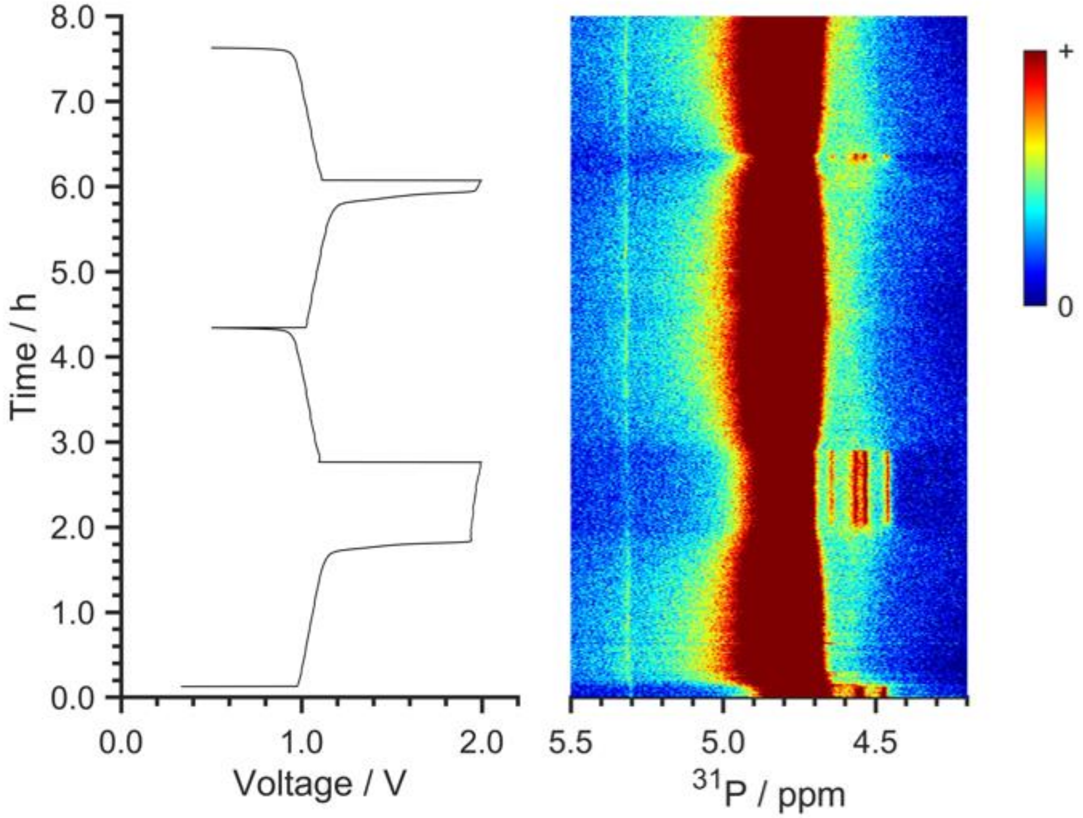

Fig. S 19: ^{31}P NMR spectra of fresh FMN. (left) Voltage of a freshly prepared 60 mM FMN^{3-} versus 0.2 M $K_4[Fe(CN)_6]$ and 0.05 M $K_3[Fe(CN)_6]$ in 1 M KOH/D_2O full cell as a function of time. During charge, a constant current density of 10 mA cm^{-2} (−50 mA) was applied until 2.0 V was reached. During discharge, a constant current density of −10 mA cm^{-2} (−50 mA) was applied until 0.5 V was reached. (right) ^{31}P NMR spectra of the anolyte. The color bar indicates the intensity of resonance in positive arbitrary units. The acquisition time per NMR spectrum was 40 s.

3.5 ^{31}P NMR – Aged Sample

The second cycle on the aged sample is shown in Fig. S 20. Before application of a current and during the first plateau (1.1 V), the same behavior as for the fresh sample was observed. During the second charging plateau, the same trend seen during the voltage plateau at 1.7 V in the fresh sample was observed, i.e., the major signal sharpens, the minor signals at lower chemical shift appear again and the signal at 5.30 ppm becomes less intense. During the discharge the same trends and behavior as that observed for the fresh sample were seen.

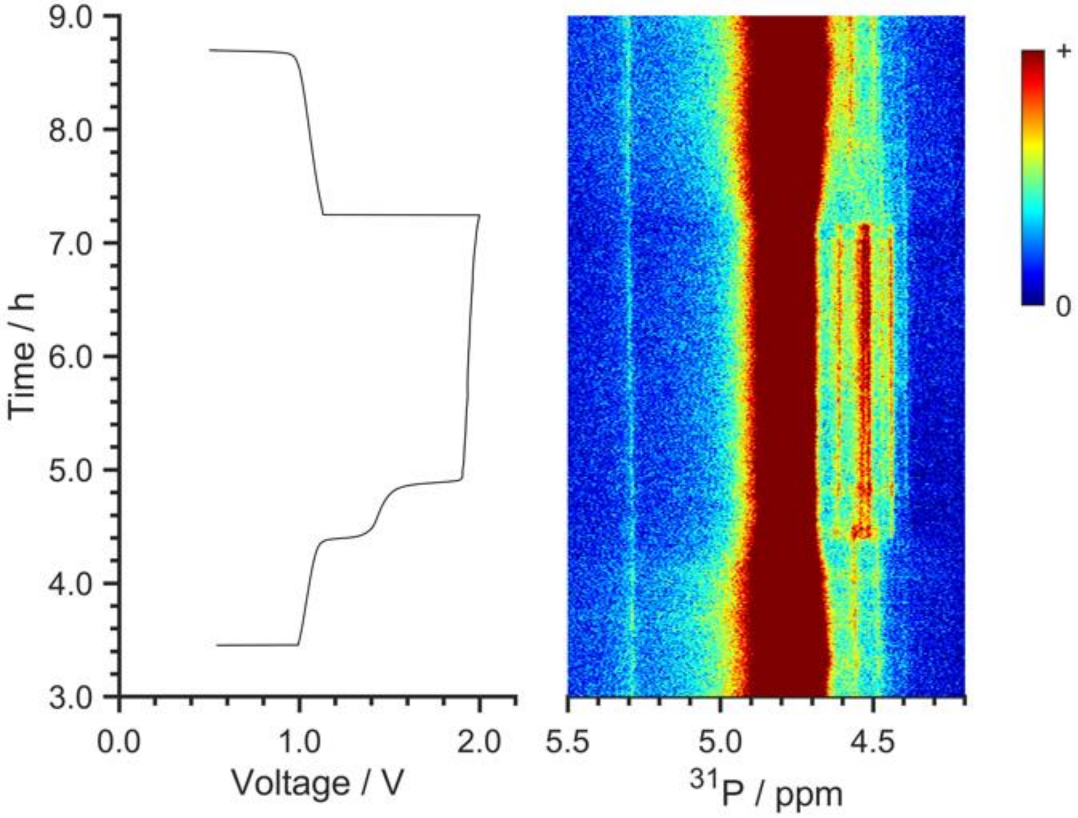

Fig. S 20: ^{31}P NMR spectra of aged FMN. (left) Voltage of a freshly prepared 60 mM FMN^{3-} versus 0.2 M $K_4[Fe(CN)_6]$ and 0.05 M $K_3[Fe(CN)_6]$ in 1 M KOH/D_2O full cell as a function of time. During charge, a constant current density of 10 mA cm^{-2} (50 mA) was applied until 2.0 V was reached. During discharge, a constant current density of –10 mA cm^{-2} (−50 mA) was applied until 0.5 V was reached. (right) ^{31}P NMR spectra of the anolyte. The color bar indicates the intensity of resonance in positive arbitrary units. The acquisition time per NMR spectrum is 40 s.

Supplementary Note 4: Further in-situ studies of the hydrolyzed flavin mononucleotide as an anolyte

4.1 Degradation products/Hydrolysis mechanisms

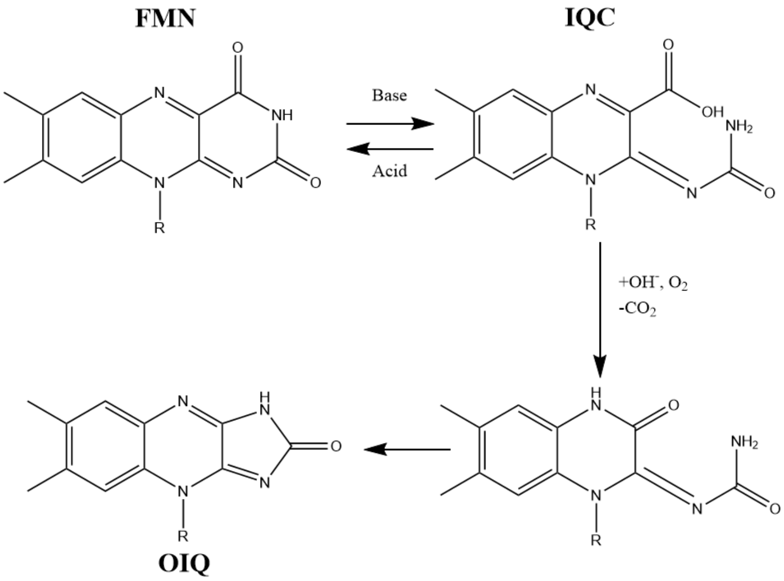

Fig. S 21: Potential hydrolysis mechanism of FMN. Potential hydrolysis mechanism of FMN and the formation of IQC, eventually leading to ring closure and the formation of OIQ.

There are multiple hydrolysis mechanisms reported in the literature [10,13,14] as seen in Fig. S 21 and Fig. S 22. Fig. S 21 shows the hydrolysis under aerobic conditions. These conditions lead to an attack in position 4 of FMN as position 1 (as in Fig. S 1) is sterically hindered by the side chain. This attack leads to a ring opening towards (E)-3-(carbamoylimino)-6,7-dimethyl-4-(2,3,4-trihydroxy-5-(phosphonooxy)pentyl)-3,4-dihydroquinoxaline-2-carboxylic acid (IQC). In aerobic conditions, IQC can undergo reduction leading to a ring closure with water loss to form 5-(6,7-dimethyl-2-oxo-1,2-dihydro-4H-imidazo[4,5-b]quinoxalin-4-yl)-2,3,4-trihydroxypentyl dihydrogen phosphate (OIQ). Conversely in Fig. S 22, hydrolysis takes place in anaerobic conditions. IQC is also formed here, but it can also undergo further hydrolysis when position 1 is not sterically hindered. The final molecule is an acidic molecule 4-(D-ribo-2,3,4-trihydroxypentyl-5'-phosphate)-3-oxo-3,4-dihydroquinoxaline-2-carboxylate (RQC) or anion in basic conditions, which involves splitting off of urea. We propose that RQC is the hydrolyzed product for FMN for reasons outlined in the following paragraphs.

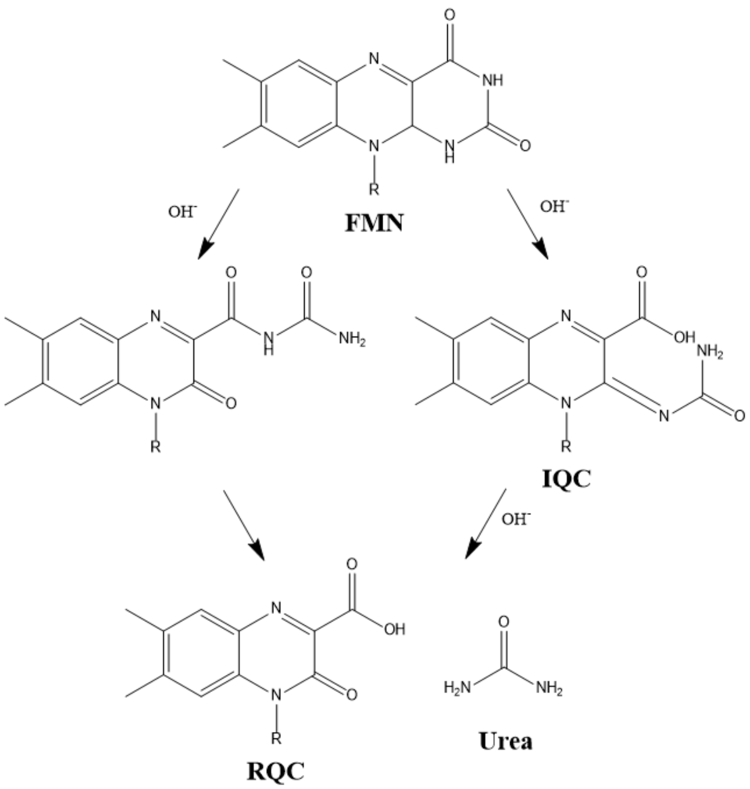

Fig. S 22: Hydrolysis mechanism with urea. Mechanism of the hydrolysis of flavin mononucleotide and release of urea on reaction with base.

4.2 ^{13}C NMR and Infrared analysis

A hydrolyzed sample prepared by heating FMN^{3-} at pH 14 for 2 h at 90°C was analyzed with ^{13}C NMR (Fig. S 23, Fig. S 24). The assignment of the resonances was made based on heteronuclear single quantum correlation (HSQC, Fig. S 25), heteronuclear multiple bond correlation (HMBC, Fig. S 26) and a separate spectrum of a sample of urea in 1 M KOH/D_2O (Fig. S 24 insert). All resonances could be assigned to either the degradation molecule RQC^{3-}or urea. The impurity seen at 168 ppm is tentatively ascribed to ICQ (the carbon of the moiety that is lost to form RQC^{3-} and urea).

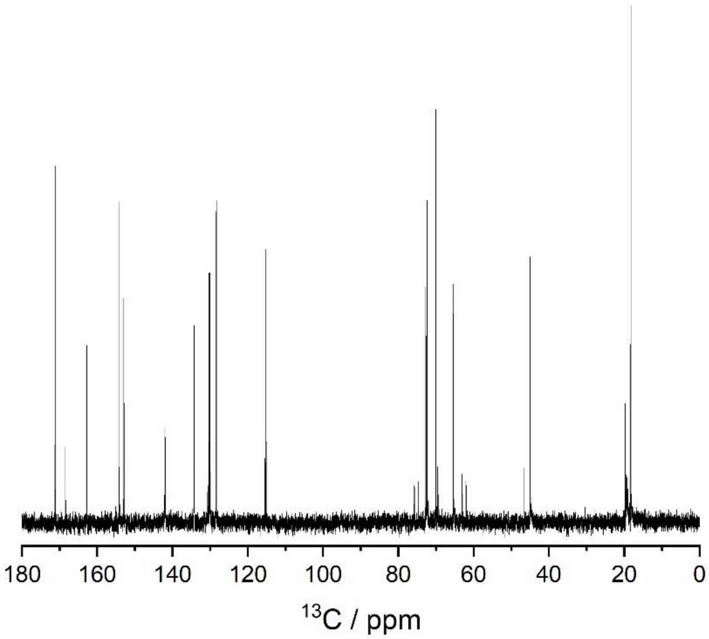

Fig. S 23: ^{13}C NMR spectrum of a hydrolyzed FMN^{3-}. ^{13}C NMR spectrum of a hydrolyzed FMN^{3-}/RQC^{3-} sample in 1 M KOH/D_2O.

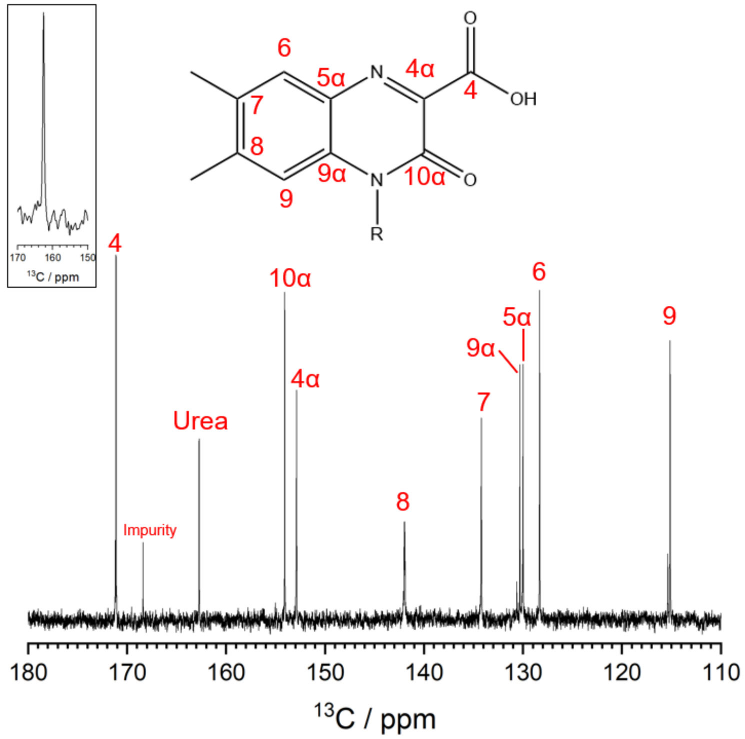

Fig. S 24: Enlarged ^{13}C NMR spectrum of a hydrolyzed FMN^{3-}. Enlarged ^{13}C NMR from Fig. S 23 with the assignment of the aromatic region plus an insert of a ^{13}C NMR of urea in 1 M KOH/D_2O.

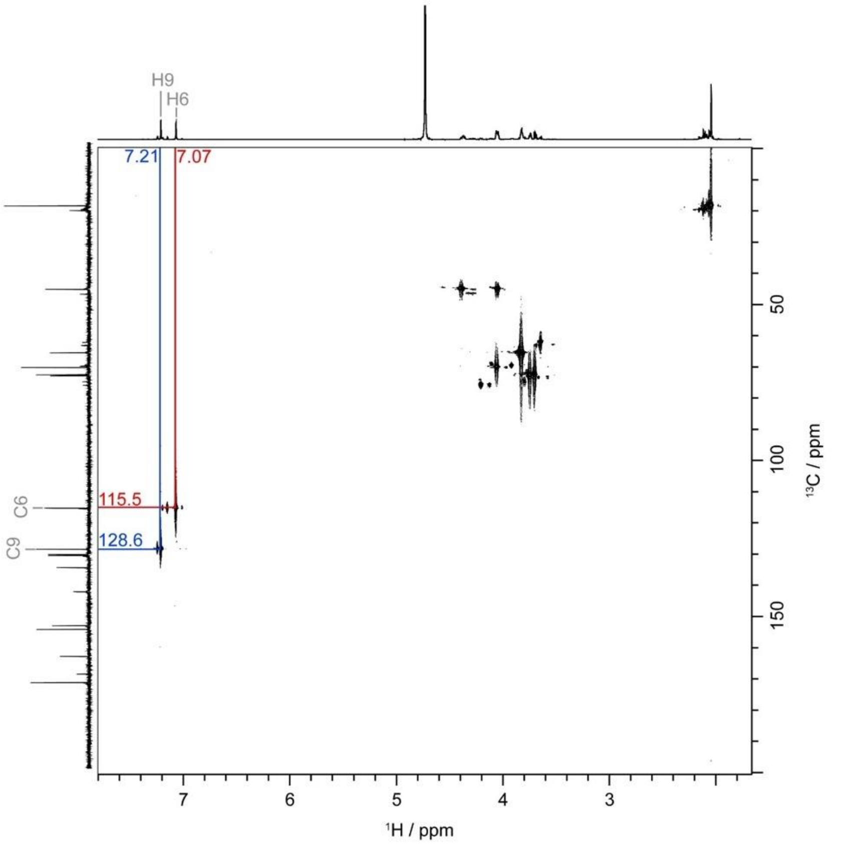

Fig. S 25: HSQC of RQC. Heteronuclear single quantum correlation (HSQC) spectrum of a RQC^{3-} sample in 1 M KOH/D_2O.

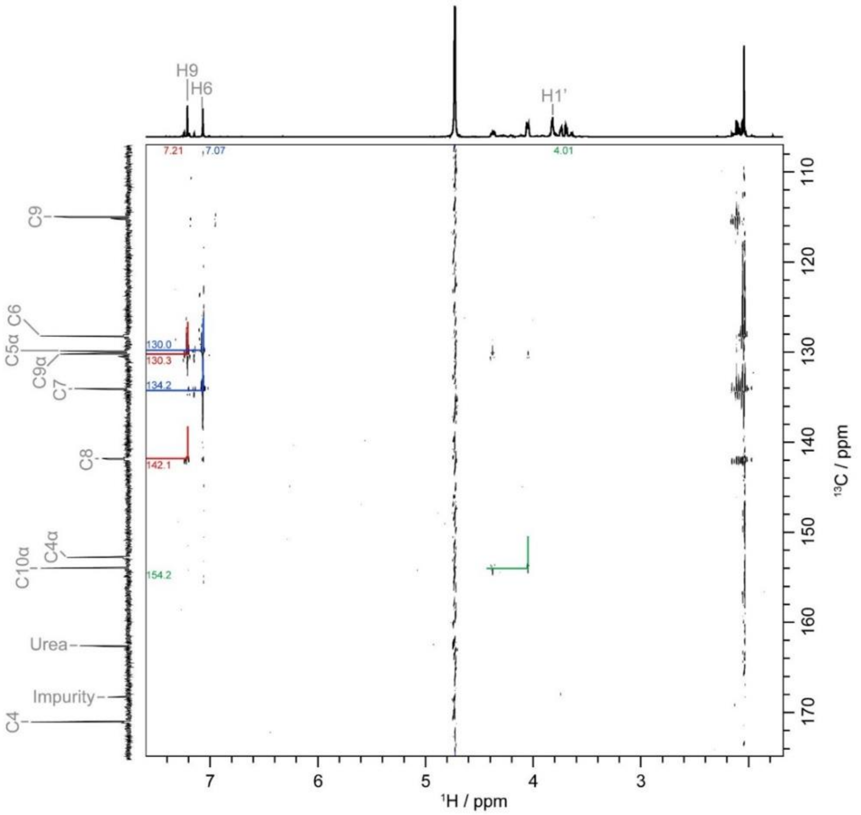

Fig. S 26: HMBC of RQC. Heteronuclear multiple bond correlation (HMBC) spectrum of a RQC^{3-} sample in 1 M KOH/D_2O.

In addition to the ^{13}C NMR, a ^{15}N NMR spectrum of the same sample was acquired (Fig. S 27). Even though the signal to noise is poor, two distinct ^{5}N resonances are seen: one consistent with a pyridine functionality (approx. 310 ppm, position 5) and a second for an amide (approx. 120 ppm, position 10). This is consistent with that expected for the degradation product RQC^{3-}. A weak signal at around 75 ppm is just visible close to the chemical shift position for urea. The other spikes appear to be artifacts as a blank sample show the same resonances, i.e., approximately 251 ppm, 352 ppm. Four distinct resonances are expected for IQC, which is not the case here.

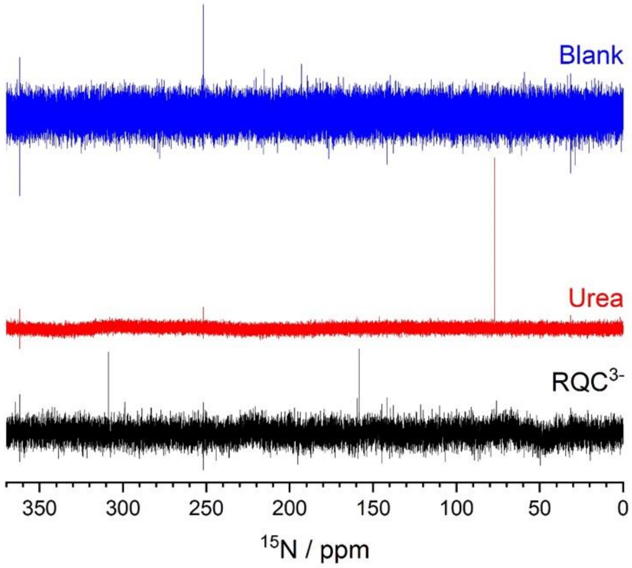

Fig. S 27: ^{15}N NMR of a RQC. ^{15}N NMR of a RQC^{3-} sample (black), urea (red) in 1 M KOH/D_2O and a blank (blue).

According to the literature[13] molecule OIQ has a very distinct IR band at about 1720 cm^{-1}, assigned to a carbonyl group within a 5-membered ring, which we do not observe (Fig. S 28). The IR spectrum instead contains bands around 1500 and 1600 cm^{-1} which are consistent with the presence of carbonyl groups in RQC^{3-}.

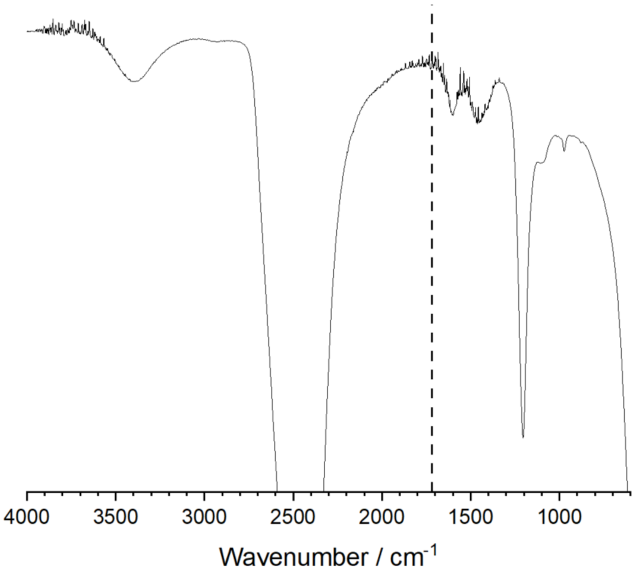

Fig. S 28: IR of RQC. Infrared spectrum of a RQC^{3-} sample in 1 M KOH/D_2O. The dashed line at 1720 cm^{-1} shows the expected absorption from OIQ.

Note that Nambufu et al. in their analysis of degradation of their bifunctional flavin derived molecule have proposed that degradation occurs via a molecule related to ICQ;[10] their proposed degradation product is likely favored in their system since they do not have an R (glycol) group attached to their flavin derivative, as shown below for their proposed hydrolysis product (Fig. S 29).

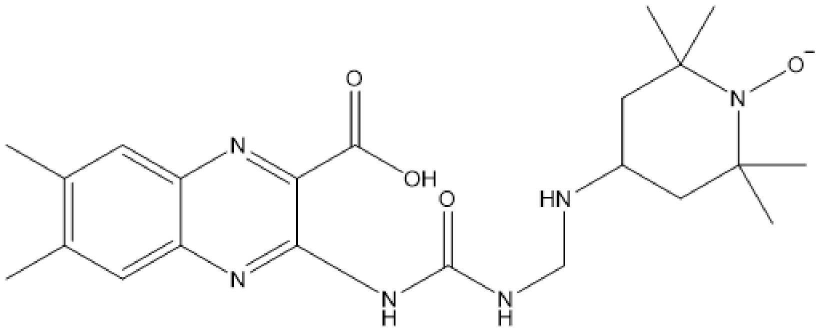

Fig. S 29: Literature degradation product. Degradation product of the bifunctional flavin derived molecule according to Nambufu et al. [10]

4.3 Electrochemistry of hydrolyzed FMN^{3-}: CV

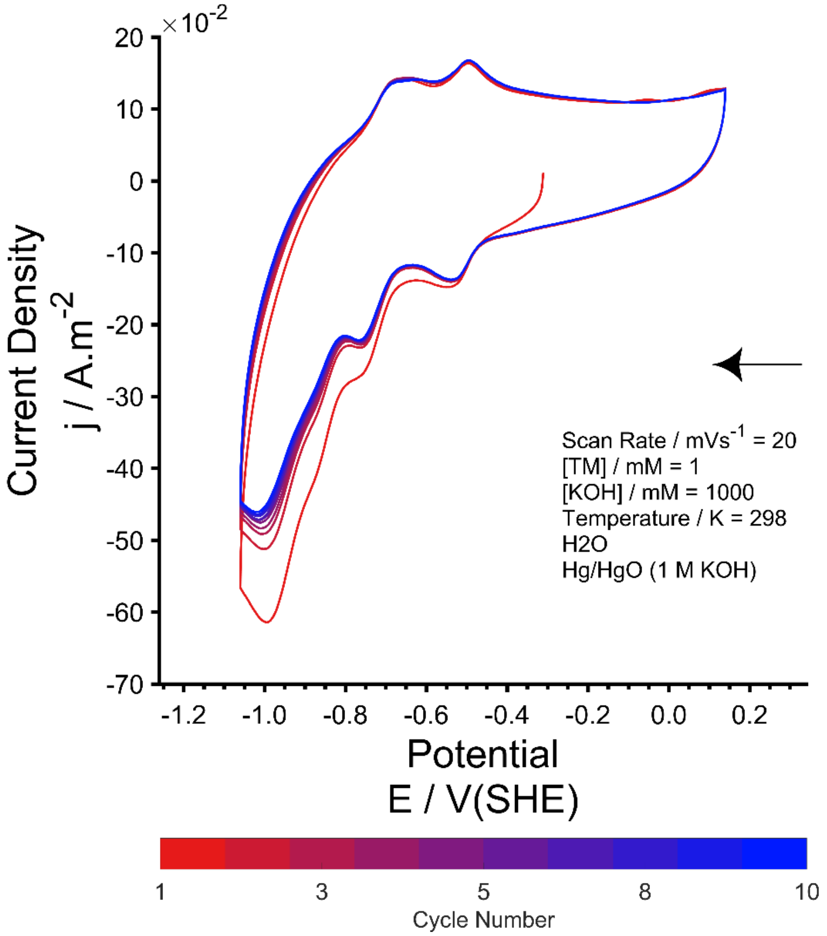

Fig. S 30: CV of RQC. CV of 1 mM hydrolyzed FMN^{3-} in 1 M KOH in degassed D_2O.

Besides cycling the hydrolyzed FMN^{3-} (RQC^{3-}) in a RFB (Fig. 3), we also analyzed RQC^{3-} using CV, measurements being performed on a freshly hydrolyzed sample (Fig. S 30). Two redox couples were observed: −0.53 V and −0.50 V versus SHE, and −0.76 V and −0.67 V versus SHE. Additionally, an irreversible reduction peak (−1.10 V versus SHE) was observed. The first redox couple is attributed to FMN^{3-}. The second redox couple is assigned to degradation products of FMN^{3-}.

The irreversible reduction peak (−1.10 V versus SHE) was previously observed during the long-term CV experiment on FMN^{3-} (Fig. S 11). Here (Fig. S 30), the reduction peak is the dominant electrochemical process. Since the sample is mainly RQC^{3-}, we assign this electrochemical process to the reduction of RQC^{3-}. This agrees with the work presented (Fig. 3) as the RFB performance of RQC^{3-} shows that it can only be reduced under standard operating conditions, and the CV lacks a defined corresponding oxidative peak for this process.

4.4 Electrochemical Cycling of RQC^{3-}

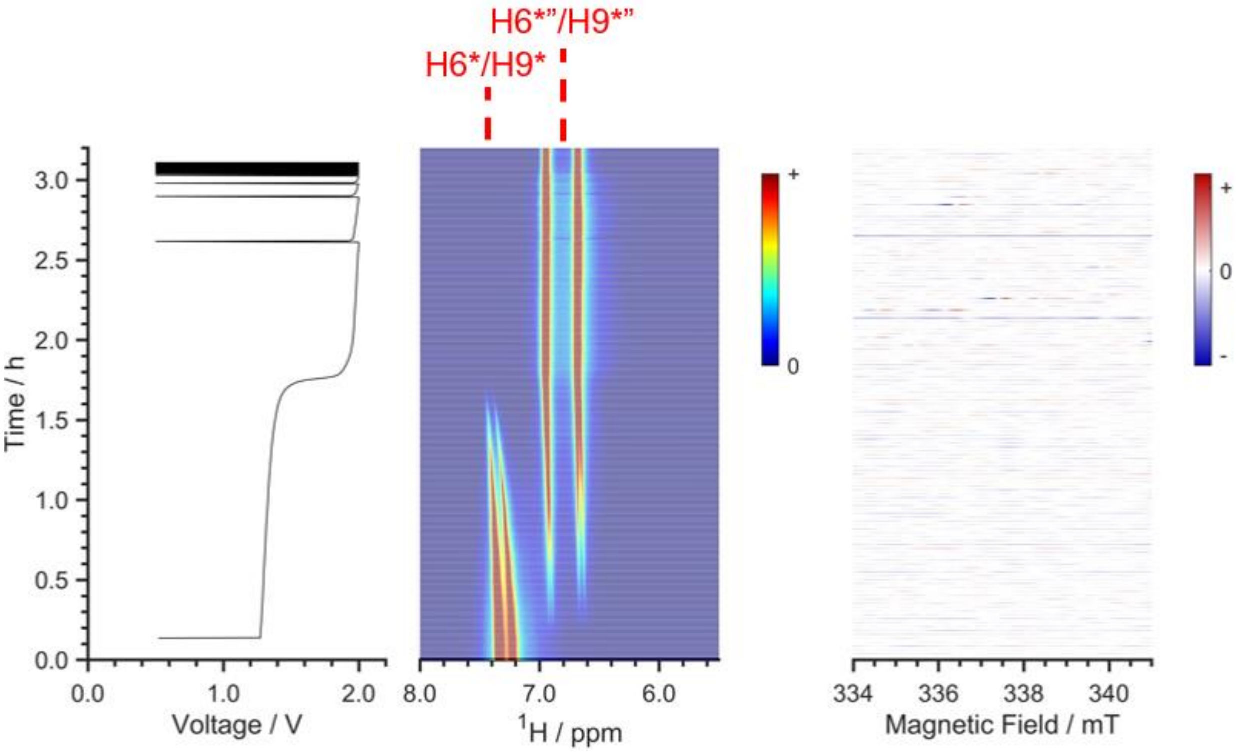

Fig. S 31: in-situ NMR analysis of RQC. (left) Voltage profile of a 60 mM RQC^{3-} solution versus 0.2 M $K_4[Fe(CN)_6]$ and 0.05 M $K_3[Fe(CN)_6]$ in 1 M KOH/D_2O full cell as a function of time. During charge, a constant current density of 10 mA cm^{-2} (50 mA) was applied until 2.0 V was reached. During discharge, a constant current density of –10 mA cm^{-2} (−50 mA) was applied until 0.5 V was reached. (middle) ^{1}H NMR (5.5 ppm to 8.0 ppm) spectra of the anolyte (RQC^{3-}/RQC^{5-}). The color bar indicates the intensity of resonance in positive arbitrary units. The acquisition time per NMR spectrum was 40 s. (Right) EPR spectra of the anolyte. The acquisition time per EPR spectrum was 95 s, with a scanning time of 60 s, a coupling time of 30 s and a delay time of 5 s. The color bar indicates the intensity of the resonance in arbitrary units. Note that a different colormap was applied to the EPR spectra because of the presence of negative peak intensities.

The EPR spectra corresponding to the data presented in Figures 3c, d, e is shown in (Fig. S 31). This shows that no paramagnetic species is formed during this experiment which further confirms that the second charge plateau being attributed to hydrolyzed FMN^{3-} (RQC^{3-}). The full ^{1}H NMR spectra can be seen in (Fig. S 32).

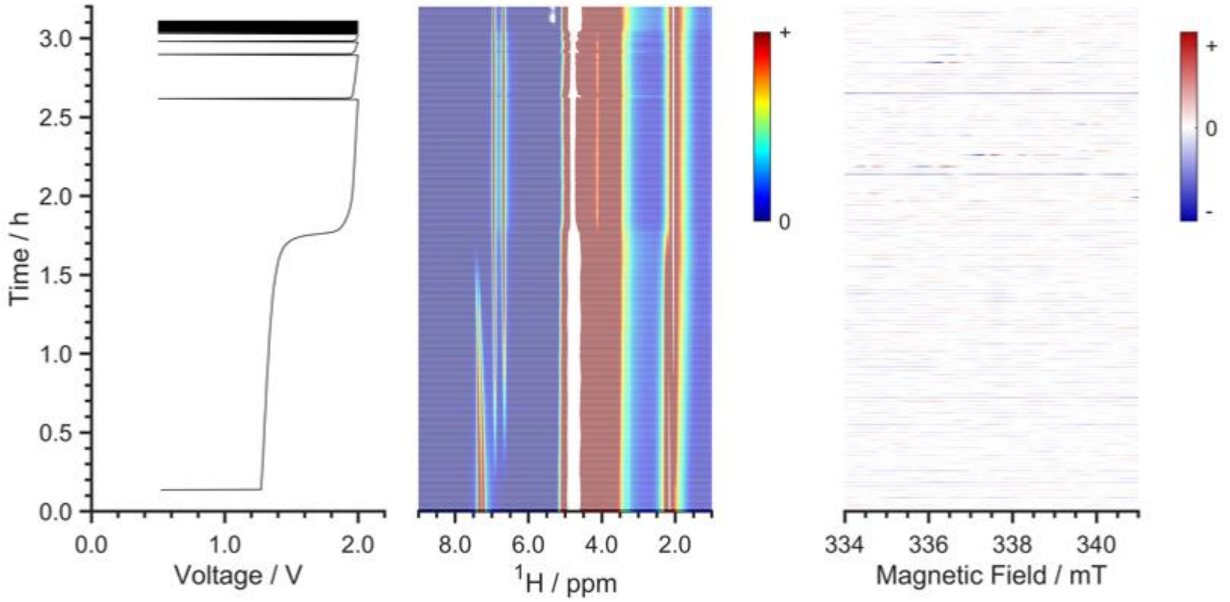

Fig. S 32: in-situ analysis of RQC. (left) Voltage profile of a 60 mM RQC^{3-} solution versus 0.2 M $K_4[Fe(CN)_6]$ and 0.05 M $K_3[Fe(CN)_6]$ in 1 M KOH/D_2O full cell as a function of time. During charge, a constant current density of 10 mA cm^{-2} (50 mA) was applied until 2.0 V was reached. During discharge, a constant current density of –10 mA cm^{-2} (−50 mA) was applied until 0.5 V was reached. (middle) Full ^1H NMR (1 ppm to 9 ppm) spectra of the anolyte (RQC^{3-}/RQC^{5-}). The color bar indicates the intensity of resonance in positive arbitrary units. The acquisition time per NMR spectrum was 40 s. (Right) EPR spectra of the anolyte. The acquisition time per EPR spectrum was 95 s, with a scanning time of 60 s, a coupling time of 30 s and a delay time of 5 s. The color bar indicates the intensity of the resonance in arbitrary units. Note that a different colormap was applied to the EPR spectra because of the presence of negative peak intensities.

4.5 DFT Calculations of RQC

The calculated structures of RQC^{3-}, $RQC^{4-\bullet}$, RQC^{5-} are shown in Fig. S 33, Fig. S 34, Fig. S 35.

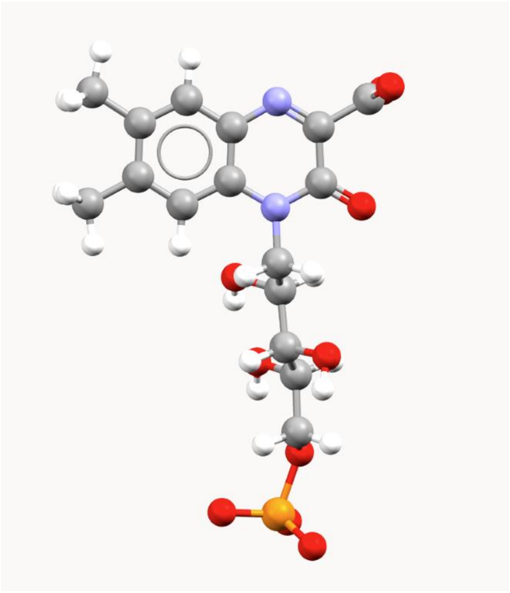

Fig. S 33: DFT calculated structure of RQC^{3-}.

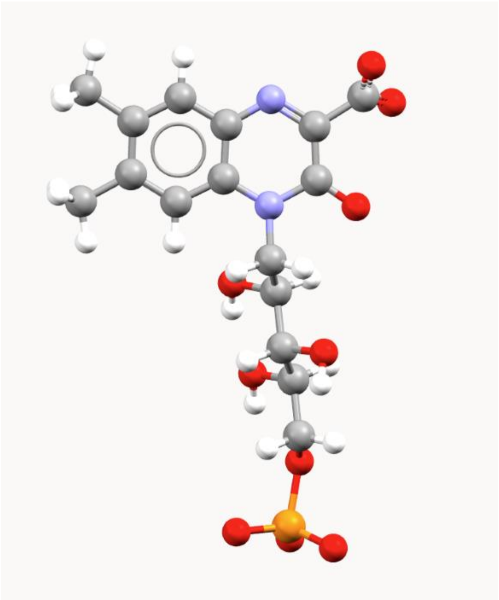

Fig. S 34: DFT calculated structure of RQC$^{4-•}$.

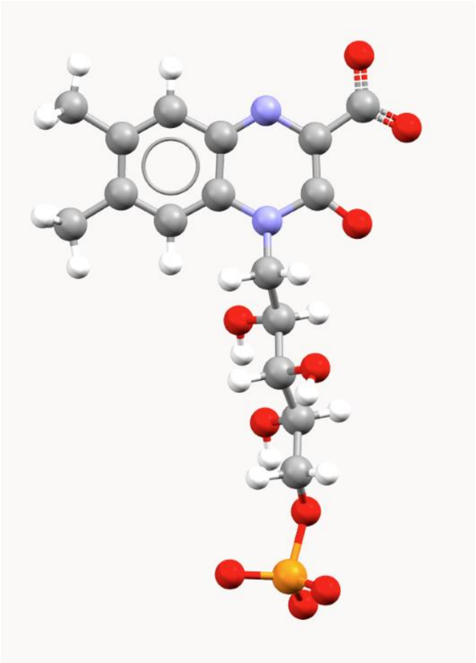

Fig. S 35: DFT calculated structure of RQC^{5-}.

4.6 Voltage Holds

As shown (Fig. 3c, d, e), RQC^{5-} cannot be oxidized under the standard battery cycling conditions used for the RFBs presented in this work. Therefore, voltage holds and deep discharges were explored in order to investigate whether electrochemical oxidation of RQC^{5-} could be carried out (Fig. S 36, Fig. S 37, Fig. S 38).

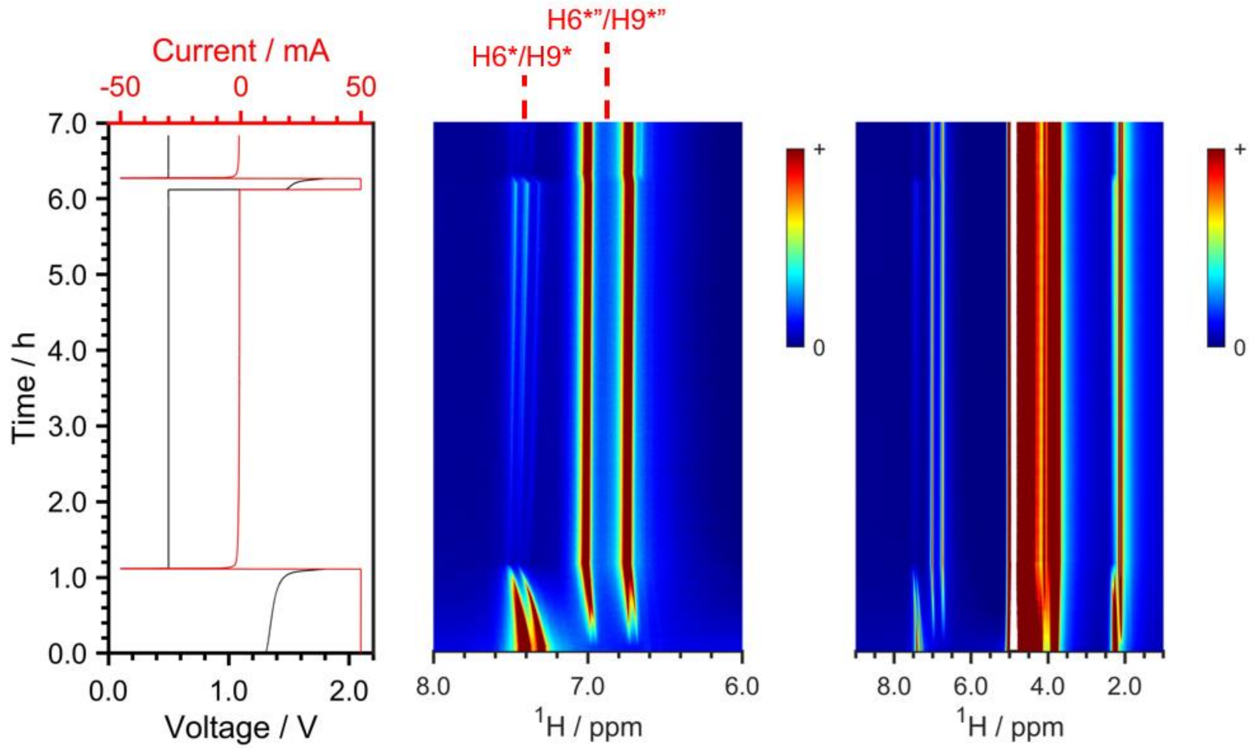

Fig. S 36: 0.5 V Voltage hold to oxidize RQC. (left) Voltage (black) and current (red) profile of a 60 mM RQC^{3-} (freshly hydrolyzed FMN^{3-}) solution versus 0.2 M $K_4[Fe(CN)_6]$ and 0.05 M $K_3[Fe(CN)_6]$ in 1 M KOH/D_2O full cell as a function of time. During charge, a constant current density of 10 mA cm^{-2} (50 mA) was applied until 1.8 V was reached. During discharge, a constant current density of –10 mA cm^{-2} (-50 mA) was applied until 0.5 V was reached. The voltage was then held at 0.5 V was held for 5 h. The battery was then charged and then discharged again with ±50 mA. (middle) ^1H NMR spectra (6 ppm to 8 ppm) of the anolyte. (right) ^1H NMR spectra (1 ppm to 9 ppm) of the anolyte. The color bar indicates the intensity of resonance in positive arbitrary units. The acquisition time per NMR spectrum is 40 s.

Firstly, a voltage hold was investigated (Fig. S 36) in combination with in-situ ^1H NMR to follow the reaction. 20 ml of freshly hydrolyzed solution was reduced to RQC^{5-} by charging the battery at a current density of 10 mA cm^{-2} (50 mA) until the cut-off voltage of 1.8 V. A negative current density of −10 mA cm^{-2} (−50 mA) was then applied to the system, resulting in an immediate decrease in cell voltage to 0.5 V. This voltage was held for 5 h before the system was charged again at 10 mA cm^{-2} to 1.8 V. The ^1H NMR spectra behaved as explained for Figure 3c, d, e during the charge plateau: signals H6* and H9* broadened and gradually disappeared, and signals H6*" and H9*" increased in intensity. However, during the 5 h voltage hold at 0.5 V, the signal intensity of H6* and H9* increased again slightly indicating that a small amount RQC^{5-} could be oxidized by holding the system at this low cell voltage. 13% of the capacity was recovered during the subsequent charge plateau in the second cycle.

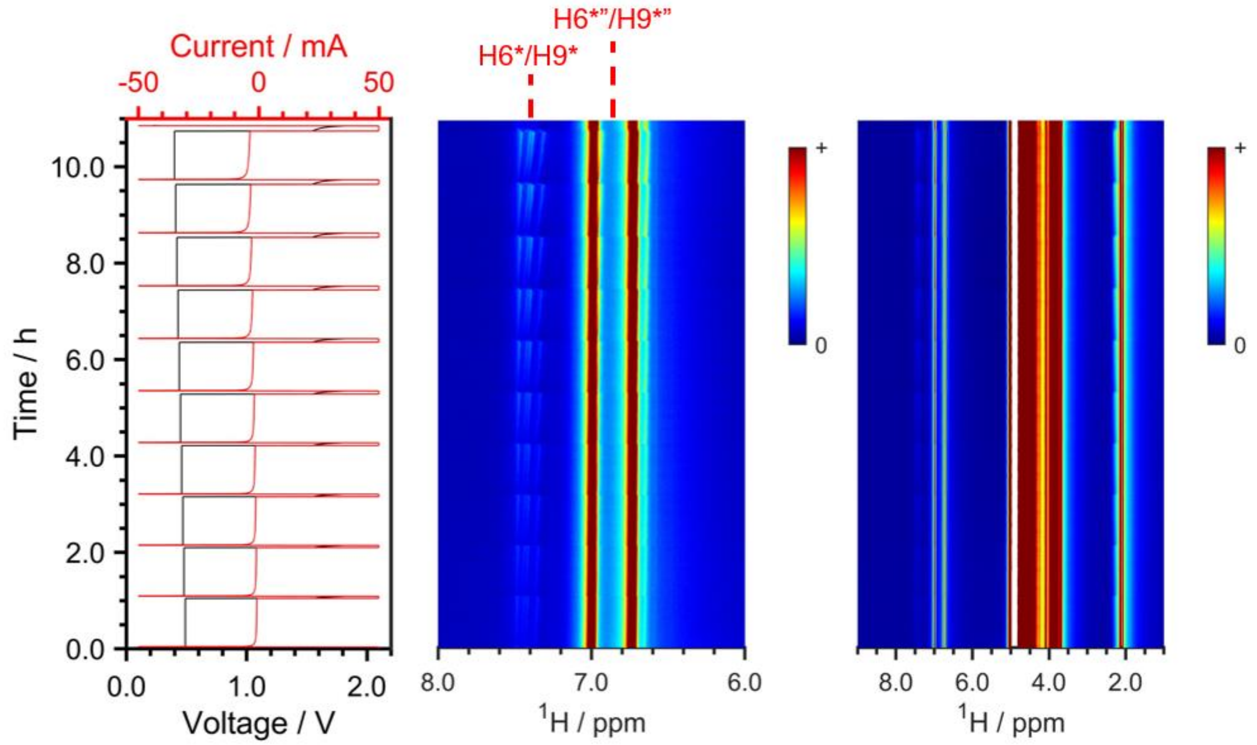

Fig. S 37: Voltage hold steps to oxidize RQC. (left) Voltage (black) and current (red) profile of a 60 mM RQC^{5-} solution (freshly hydrolyzed and reduced FMN^{3-}) versus 0.2 M $K_4[Fe(CN)_6]$ and 0.05 M $K_3[Fe(CN)_6]$ in 1 M KOH/D_2O full cell as a function of time. During charge, a constant current density of 10 mA cm^{-2} (50 mA) was applied until 1.8 V was reached. During discharge, a constant current density of –10 mA cm^{-2} (-50 mA) was applied, which was followed by 1 h voltage holds increasingly low potential (from 490 mV to 400 mV in 100 mV steps). (middle) ^{1}H NMR spectra (6 ppm to 8 ppm) of the anolyte. The color bar indicates the intensity of resonance in positive arbitrary units. The acquisition time per NMR spectrum is 40 s. (right) ^{1}H NMR spectra (1 ppm to 9 ppm) of the anolyte.

To further analyze the electrochemical oxidation of RQC^{5-}, a 20 mL solution of RQC^{5-} was held for 1h at progressively lower and lower voltages of between 490 mV and 400 mV (Fig. S 37). Upon application of −10 mA cm^{-2} to the charged solution, as before, the voltage immediately dropped to the cut-off voltage (490 mV). The battery was then held at 490 mV for 1h, after which charging of the battery was attempted at 10 mA cm^{-2}. Once 1.8 V was reached, the same negative current density (−10 mA cm^{-2}) was applied until a lower cut-off voltage of 480 mV was reached, where again the system was held for 1 h before being recharged. This cycling protocol was repeated, lowering the cut-off voltage with by 100 mV in each successive cycle with the deepest discharge being carried out at 400 mV. The experiment was followed via in-situ ^{1}H NMR. As the depth of discharge increased, more capacity was recovered. This is evidenced by the fact that after the first hold at 490 mV, 4% of capacity was recovered, but after the voltage was held at 400 mV, 9% of capacity was recovered. An increment of 0.5% additional capacity was recovered through the lowering of the voltage by 100 mV with a 1 h hold. This is supported by the ^{1}H NMR, as the signal intensity over the 1 h of H6* and H9* increases correspondingly across each hold. To further confirm this, the voltage was lowered to 350 mV for 30 minutes (Fig. S 38). After a voltage-hold of 30 minutes, 8% of the total capacity was recovered (cf. 9% at 400 mV with a hold of 1 h). These experiments indicate that deep-discharging, i.e., discharging the cell to even lower voltages, might enable a battery containing just RQC^{3-}/ RQC^{5-} to be cycled. However, lowering the voltage even further increases the risk of oxygen evolution on the anolyte side.

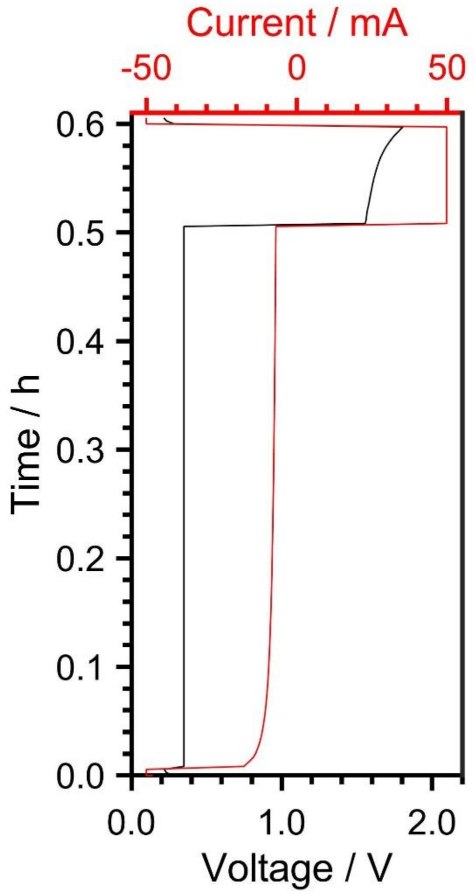

Fig. S 38: 350 mV voltage hold to oxidize RQC. Voltage (black) and current (red) profile of a 60 mM RQC^{5-} solution (freshly hydrolyzed and reduced FMN^{3-}) versus 0.2 M $K_4[Fe(CN)_6]$ and 0.05 M $K_3[Fe(CN)_6]$ in 1 M KOH/D_2O full cell as a function of time. During charge, a constant current density of 10 mA cm^{-2} (50 mA) was applied until 1.8 V was reached. During discharge, a constant current density of −10 mA cm^{-2} (−50 mA) was applied. The 30 min voltage hold was at 350 mV.

The possibility of self-discharge was also considered. To test whether this may have been responsible for the recovered capacity, a solution of RQC^{3-} was reduced to RQC^{5-}, and then rested at OCV for 1 h. Afterwards, charging of the solution was re-attempted (Fig. S 39). No capacity was recovered through this showing that little-to-no self-discharge of RQC^{5-} had occurred in this time.

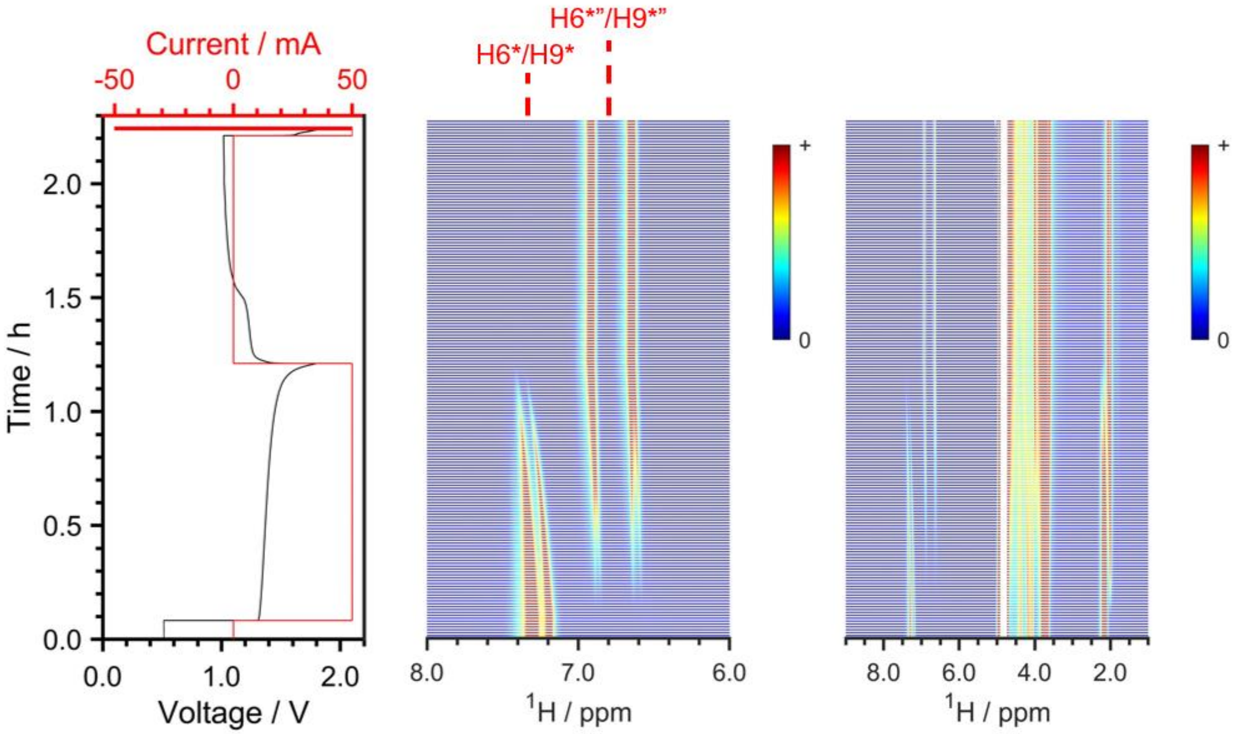

Fig. S 39: Self-discharge of RQC. (left) Voltage (black) and current (red) profile of a 60 mM RQC3− solution (freshly hydrolyzed FMN3−) versus 0.2 M $K_4[Fe(CN)_6]$ and 0.05 M $K_3[Fe(CN)_6]$ in 1 M KOH/D2O full cell as a function of time. During charge, a constant current density of 10 mA cm^{-2} (50 mA) was applied until 1.8 V was reached. During discharge, a constant current density of –10 mA cm^{-2} (−50 mA) was applied. The OCV was recorded for 1 h after the first charge cycle. (middle) ^{1}H NMR spectra (6 ppm to 8 ppm) of the anolyte. The color bar indicates the intensity of resonance in positive arbitrary units. The acquisition time per NMR spectrum is 40 s. (right) ^{1}H NMR spectra (1 ppm to 9 ppm) of the anolyte.

Supplementary Note 5: Redox mediating effect of FMN^{3-}

5.1 Redox mediating effect of FMN^{3-}

To investigate the rate that FMN^{3-} oxidizes RQC^{5-}, a similar experiment as described for Figure 4 was performed with an equilibration time of only 1 h after the addition of 20 mL of FMN^{3-} (Fig. S 40). After the 1 h of equilibration, only 50 % of the expected capacity was seen. However, during the subsequent charge, in addition to the charging plateau (1.41 V) attributed to RQC^{3-}, the charging plateau attributed to FMN^{3-} was also observed. This indicates that not all of the FMN^{3-} was reduced through oxidation of RQC^{5-} during the 1 h rest, meaning that there was FMN^{3-} available for reduction during the charge cycle. This is corroborated by the in-situ ^{1}H NMR as the signals for RQC^{5-} (H6*" and H9*") do not completely disappear, as seen in Figure 3h, indicating that a significant amount of RQC^{5-} is still present. The signals for RQC^{3-} (H6* and H9*), however, were observed to increase in intensity. During the consecutive cycles, the same behavior as explained for Figure 4 was observed. This experiment further supports the fact that the proposed chemical redox mediator reaction between FMN^{3-} and RQC^{5-} is slow in comparison to the heterogeneous electron transfer that occurs between the electrode and the redox active species during cycling.

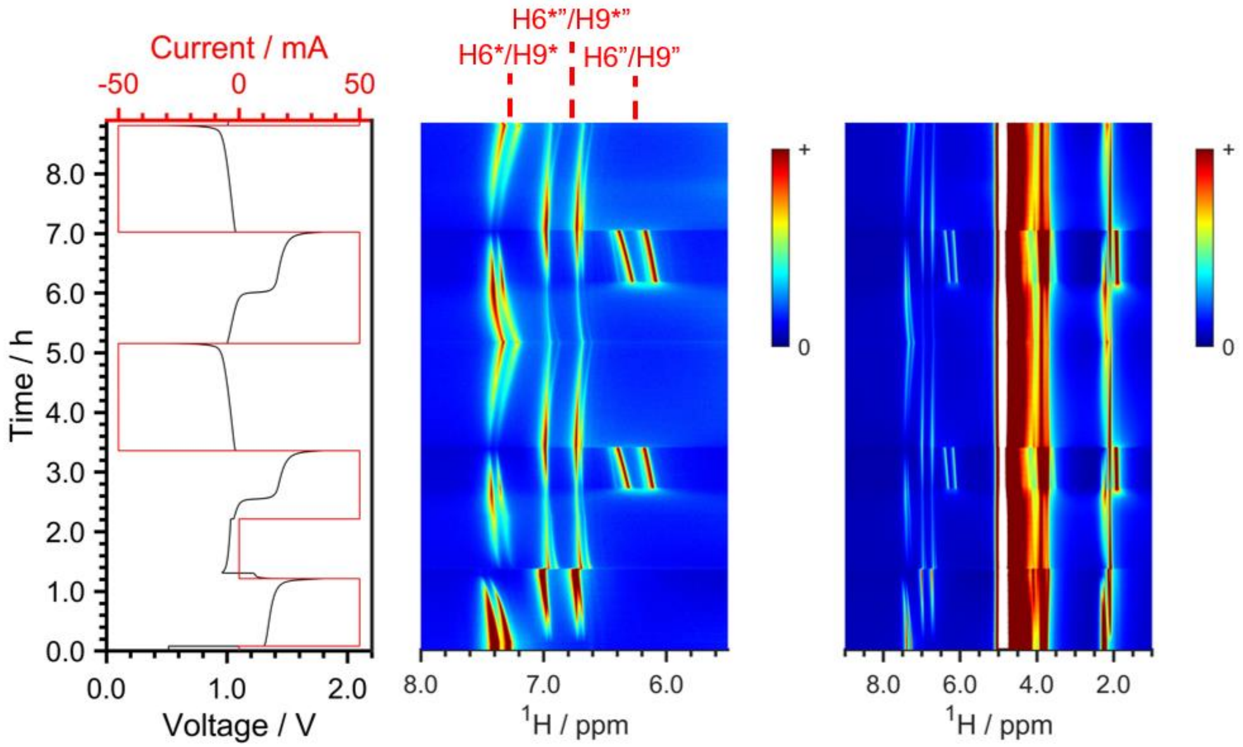

Fig. S 40: Redox mediating effect of FMN during 1hr rest. (left) Voltage (black) and current (red) profile of a mixed 60 mM RQC^{3-} and 60 mM FMN^{3-} solution versus 0.2 M $K_4[Fe(CN)_6]$ and 0.05 M $K_3[Fe(CN)_6]$ in 1 M KOH/D_2O full cell as a function of time. (middle) ^{1}H NMR spectra (6 ppm to 8 ppm) of the anolyte (RQC^{3-}/RQC^{5-} and FMN^{3-}/FMN^{5-}). The color bar indicates the intensity of resonance in positive arbitrary units. The acquisition time per NMR spectrum is 40 s. (right) ^{1}H NMR spectra (1 ppm to 9 ppm) of the anolyte.

In an attempt to recover the full capacity of the system during consecutive cycling, a 90-minute voltage hold at 0.5 V was incorporated into the experiment described above (Fig. S 40) and the results are shown in Fig. S 41**.** Previously, due to the slow redox reaction, only 80% of the overall capacity was recovered (Fig. S 40). However, addition of a 90-minute voltage hold at 0.5 V demonstrated that 100% of the expected

capacity could be recovered with 50% of the reduction of FMN^{3-} and 50% of the reduction of RQC^{3-}. During discharge, however, we still only recover 80% of the expected capacity. These results are further supported by the disappearance of the signals from RQC^{5-} (H6*'' and H9*'') in the ^{1}H NMR during the voltage hold. At 0.5 V, oxidation of FMN^{5-} to FMN^{3-} occurs. The freshly produced FMN^{3-} is then able to oxidize RQC^{5-} to RQC^{3-} via redox mediation. The reformed FMN^{5-} can then be oxidized again at the electrode.

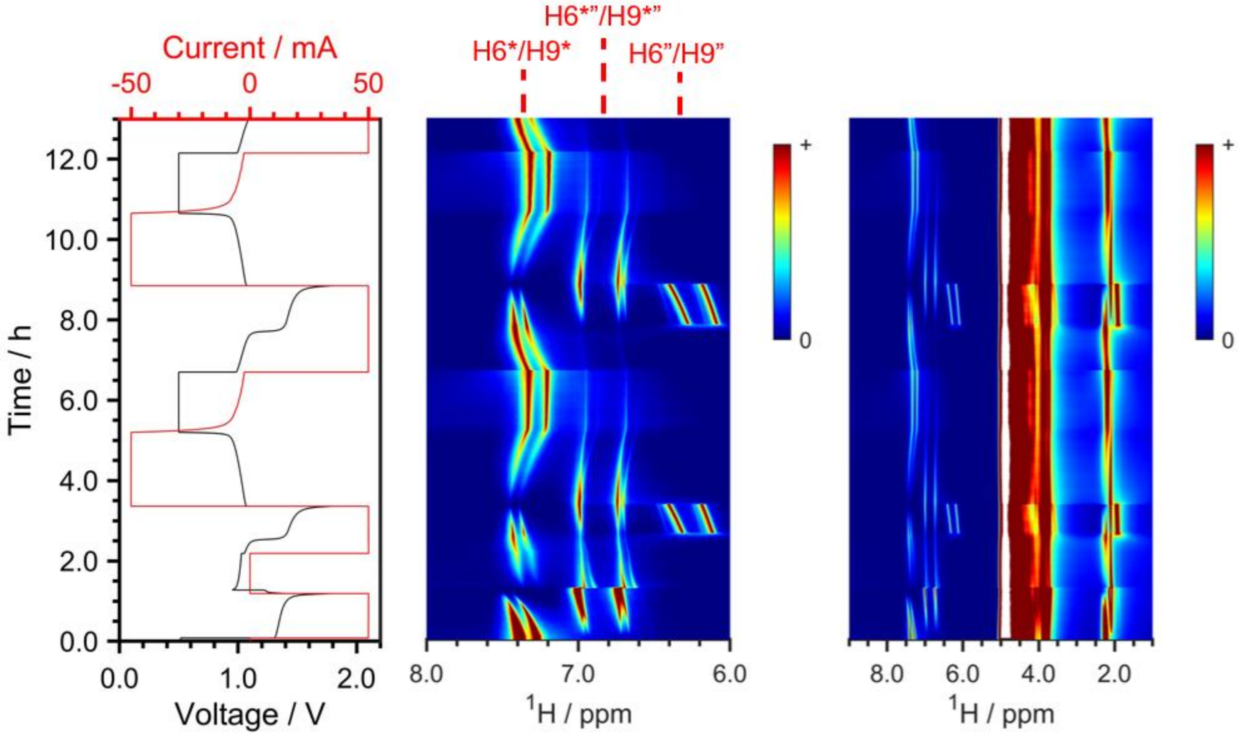

Fig. S 41: Redox mediating effect of FMN during 1hr rest plus 0.5 V voltage hold. (left) Voltage (black) and current (red) profile of a mixed 60 mM RQC^{3-} and 60 mM FMN^{3-} solution versus 0.2 M $K_4[Fe(CN)_6]$ and 0.05 M $K_3[Fe(CN)_6]$ in 1 M KOH/D_2O full cell as a function of time. (middle) ^{1}H NMR spectra (6 ppm to 8 ppm) of the anolyte (RQC^{3-}/RQC^{5-} and FMN^{3-}/FMN^{5-}). The color bar indicates the intensity of resonance in positive arbitrary units. The acquisition time per NMR spectrum is 40 s. (right) ^{1}H NMR spectra (1 ppm to 9 ppm) of the anolyte.

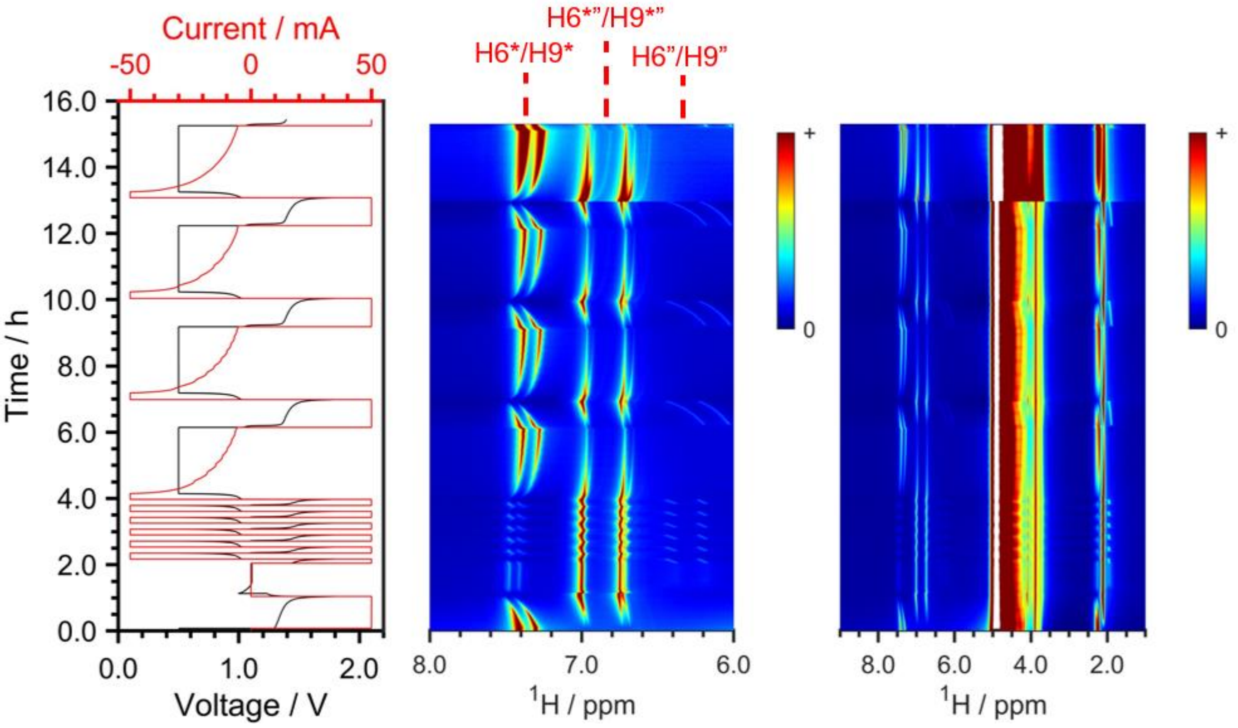

Fig. S 42: Redox mediating effect of FMN with a lower volume. (left) Voltage (black) and current (red) profile of a mixed 60 mM RQC^{3-} and 60 mM FMN^{3-} solution versus 0.2 M $K_4[Fe(CN)_6]$ and 0.05 M $K_3[Fe(CN)_6]$ in 1 M KOH/D_2O full cell as a function of time. (middle) ^{1}H NMR spectra (6 ppm to 8 ppm) of the anolyte (RQC^{3-}/RQC^{5-} and FMN^{3-}/FMN^{5-}). The color bar indicates the intensity of resonance in positive arbitrary units. The acquisition time per NMR spectrum is 40 s. (right) ^{1}H NMR spectra (1 ppm to 9 ppm) of the anolyte.

To demonstrate this redox shuttling by FMN^{3-}, an experiment with a lower fraction of FMN^{3-} with respect to RQC^{3-} was carried out (Fig. S 42). 18 mL of RQC^{3-} was charged by applying 10 mA cm^{-2} until 1.8 V was reached, after 5 min of rest, 2 mL of FMN^{3-} was then added to the solution and the system was allowed to equilibrate for 1 h. The battery was then cycled again between 0.5 V and 1.8 V by applying a current density of ±10 mA cm^{-2} (±50 mA) for 6 consecutive cycles; following this, a voltage hold at 0.5 V for 2 h was added to the cycling protocol.

During the charging of RQC^{3-}, the same behavior during the voltage profile and ^{1}H NMR was observed as previously described and 90% of the expected capacity was recovered. The addition of 2 mL of FMN^{3-} to the RQC^{5-} caused the voltage to decrease immediately after a resting period of 5 minutes. The solution was allowed to equilibrate for 1 h and charged again. During the second charge, only the plateau corresponding to RQC^{3-} could be observed along with the additional capacity from the 2 mL of FMN solution that was added (equal to 10 %). This indicates that the FMN^{3-} must have been reduced to FMN^{5-} during the equilibration period. On discharge, a higher capacity (16%) was observed than previously. The additional 6% likely stems from the interaction of RQC^{5-} and FMN^{3-} during discharge. The following charge and discharge cycles all also exhibit a discharge capacity of 16%.

As mentioned above, after 6 cycles a voltage-hold at 0.5 V for 2 h was added. This facilitated 77% of the capacity to be recovered: 5% due to the reduction of FMN^{3-} and 72% due to the reduction of RQC^{3-}. During the consecutive discharge, without a voltage-hold, 16% of the capacity was achieved. This experiment further emphasizes the clear correlation between the signal intensity of RQC^{3-} (H6* and H9*) and RQC^{5-}

(H6*” and H9*”). The signal intensity of FMN^{5-} (H6” and H9”) remained constant during the second charging plateau indicating the full reduction of FMN^{3-}. These results also highlight the need for a voltage-hold to recover capacity once the majority of the system has undergone hydrolysis.

To investigate if a longer voltage hold would allow for full recovery of the capacity, an experiment (Fig. S 43) was performed where 18 mL of RQC^{3-} was charged until 1.8 V was reached. To this solution, 2 mL of FMN^{3-} was added after 5 minutes, allowed to equilibrate for 1 h and then charged again to 1.8 V and discharged to 0.5 V. The voltage was held at 0.5 V for 5 h before recharging. The system behaved as previously explained (Fig. S 42) prior to the voltage-hold. During the voltage hold, however, near complete disappearance of the signals caused by RQC^{5-} (H6*” and H9*”) was observed. This indicates that the RQC^{5-} had been completely oxidized by FMN^{3-}. This was not observed during the 2 h hold (Fig. S 42). The subsequent charge plateau, after the voltage hold, demonstrated 97% of the expected capacity could be recovered. This shows that a longer voltage hold is needed to ensure complete oxidation of RQC^{5-}. It is also possible that during the 5 h voltage hold, some RQC^{5-} was oxidized by the electrode as we have shown that for a fully hydrolyzed sample, 13% of the total capacity is recoverable (Fig. S 36) via a voltage-hold.

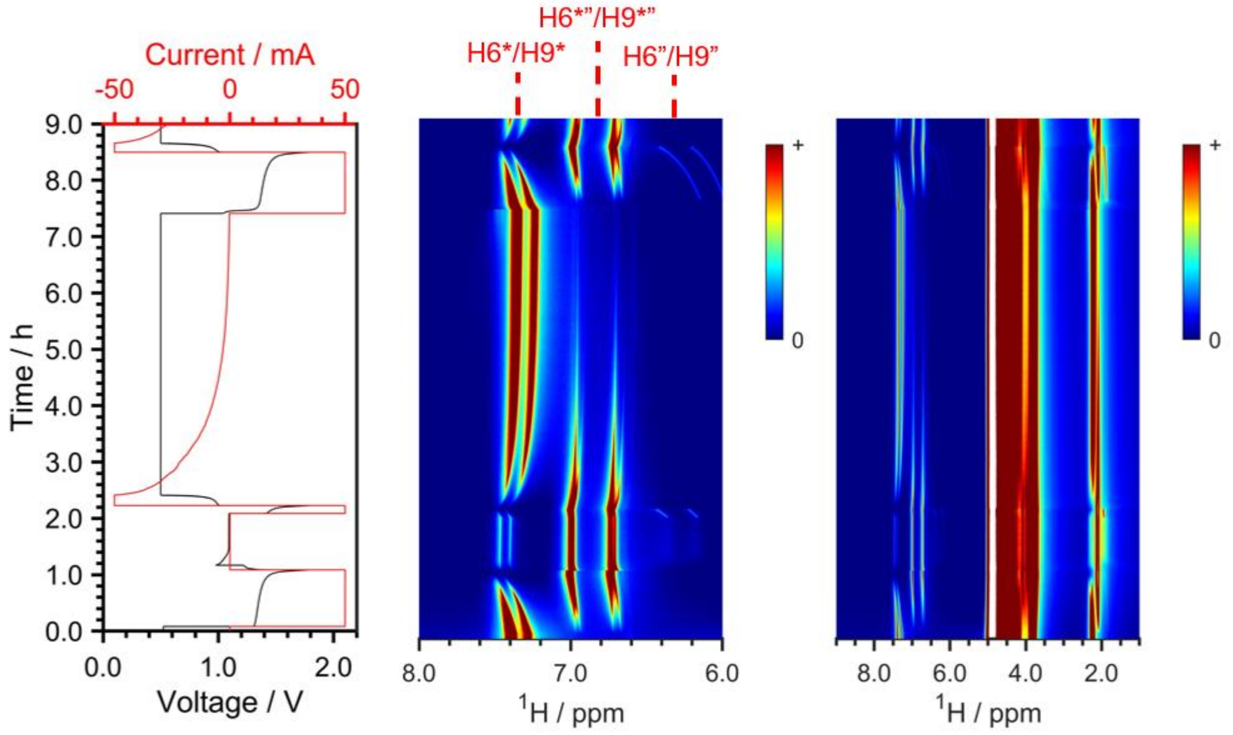

Fig. S 43: Redox mediating effect of FMN with a lower volume and 0.5 V voltage hold. (left) Voltage (black) and current (red) profile of a mixed 60 mM $RQC^{3-}$and 60 mM FMN^{3-} solution versus 0.2 M $K_4[Fe(CN)_6]$ and 0.05 M $K_3[Fe(CN)_6]$ in 1 M KOH/D_2O full cell as a function of time. (middle) ^1H NMR spectra (6 ppm to 8 ppm) of the anolyte (RQC^{3-}/RQC^{5-} and FMN^{3-}/FMN^{5-}). The color bar indicates the intensity of resonance in positive arbitrary units. The acquisition time per NMR spectrum is 40 s. (right) ^1H NMR spectra (1 ppm to 9 ppm) of the anolyte.

Supplementary Note 6: pH studies of flavin mononucleotide (pH 10 and 12)

6.1 Protonation states and solubility of flavin mononucleotide

The protonation states of flavin mononucleotide have previously been reported (Fig. S 44) [9]. In acidic solution with a pH range of 0.7 to 6.2, the major species is singly-deprotonated FMN (FMN^{-}) with the phosphate group having lost a proton. Between pH 6.2 and 10.2, the phosphate group loses another proton resulting in the predominant species being FMN^{2-} at near-neutral or weakly basic pHs. At a pH greater than 10.2 the solution largely consists of FMN^{3-}, where the nitrogen located at the third position of the isoalloxazine ring is deprotonated. FMN^{3-} is the first species under investigation in this work.

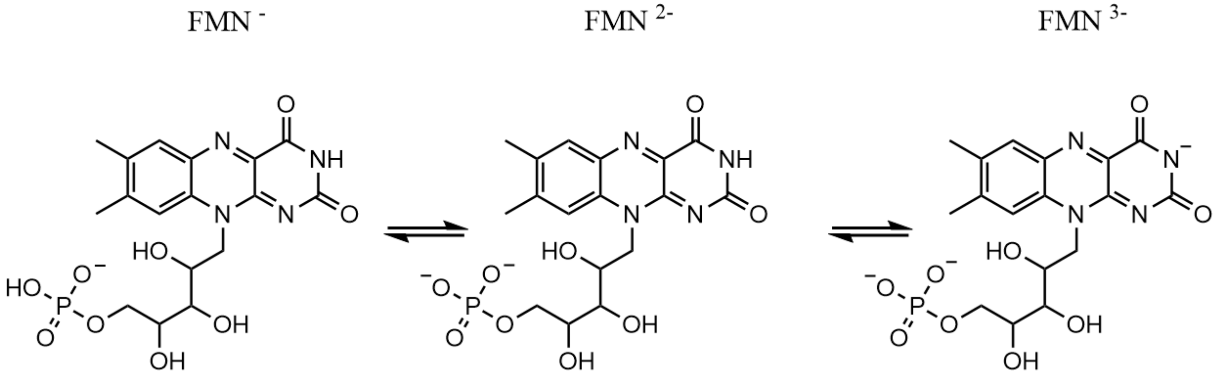

Fig. S 44: Protonation states of FMN in solution.

During the previous literature study[9], the solubility was analyzed as well as the electrochemical performance: the maximum solubilities in 1 M H_2SO_4 (pH 0.8), 1 M KCl (pH 5.5) and 1 M KOH aqueous solutions (pH 13.0) were ~10, 50, and 100 mM. Orita *et al.* [9] explained the higher polarity of FMN^{3-} in terms of its multiple negative charges, which may result in the greatest solvent-solute interactions. They further enhanced the solubility by adding nicotinamide (NA) as a solubilizing agent to the solution and achieved a maximum solubility of ~1.5 M in an aqueous solution of 1.0 M KOH and 3 M NA.

6.2 Lowering the pH to pH 10

As explained before (Fig. 3a), the hydrolysis only takes place at a pH above 12. As the most soluble species of FMN is FMN^{3-} which is present at a pH above 10.2, CVs at pH 10.3 (Fig. S 45) and pH 12.3 (Fig. S 46) were performed to assess the electrochemistry of FMN under these conditions.

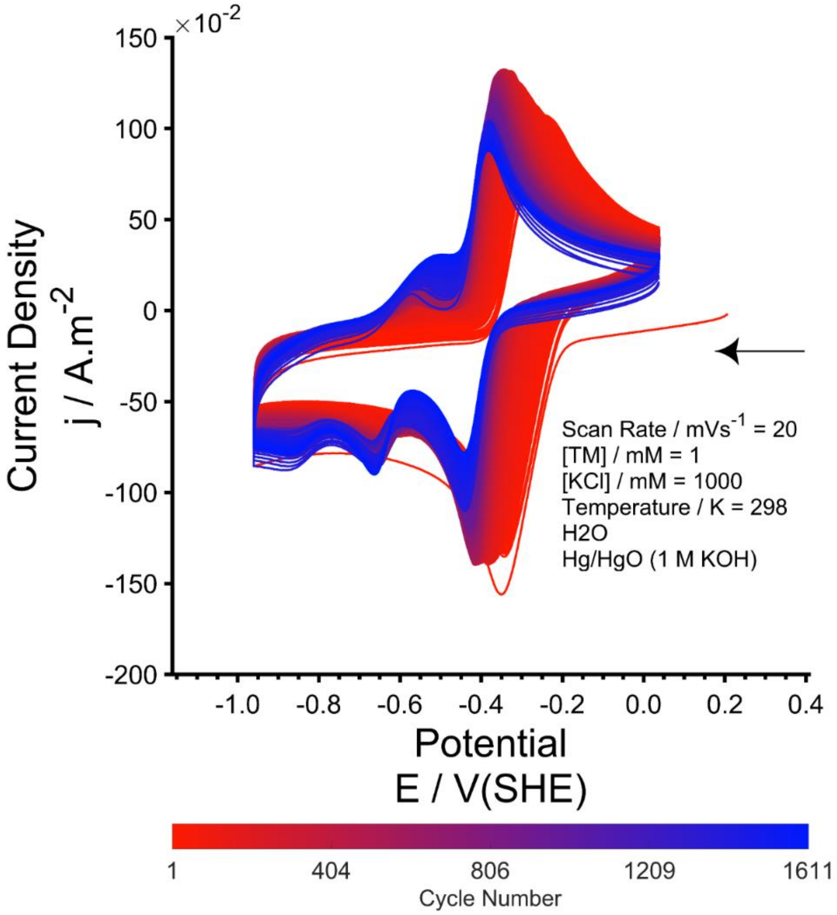

Fig. S 45: CV of FMN at pH 10.3. Long-time (24 h) CV of 1 mM FMN in 1 M KCl with KOH (pH 10.3) in degassed D_2O measured vs. the Ag/Ag^+ couple but plotted vs. SHE.

The CV at pH 10.3 (Fig. S 45) initially showed a redox couple (−0.34 V and −0.23 V versus SHE). This redox couple then shifts with time towards lower potentials and stabilizes (−0.44 V and −0.38 V versus SHE). The current intensity during both oxidation and reduction remains reasonably constant. Additionally, another redox couple, initially low in intensity, grows in (−0.66 V and −0.51 V versus SHE). Towards the end of the experiment (800 cycles), an additional reduction peak at −0.85 V versus SHE was also observed. The initial redox couple was assigned to the reduction and oxidation of FMN^{3-}. The shift towards lower potentials may be explained by a change in pH of the solution. We hypothesize that the solution increases in pH (due to water reduction, as discussed below) and the proportion of FMN^{3-} present in the solution increases.

The overall solution is less likely reduced. The ingrowing, weaker redox couple was assigned to the partial hydrolysis product of FMN^{3-} (Fig. S 11). As this redox couple grows in once the first redox couple has stabilized, it suggests that the pH of the system approaches pH 12 where hydrolysis takes place. The reduction peak (Fig. S 30) associated with hydrolysis was also observed to grow in intensity.

The initial CVs for pH 12.3 (Fig. S **Fig. S** 46) have the same features as the stabilized performance of the CV at pH 10.3 (Fig. S 45): a major redox couple (−0.44 V and −0.38 V versus SHE), a less intense redox couple (−0.66 V and −0.57 V versus SHE) and an in-growing reduction peak (−0.88 V versus SHE).

Overtime, the low-potential redox couple and the reduction peak significantly increase in intensity as the high-potential redox couple decreases in intensity. This indicates an accelerated hydrolysis in comparison to the CV at pH 10.3 (Fig. S 45), as the reduction peak occurs quicker (500 cycles). The high-potential redox couple was assigned to the redox reaction of FMN^{3-} and so these results once again indicate that FMN^{3-} breaks down due to hydrolysis. This analysis shows that lowering the pH to between 10 and 11 will aid in limiting hydrolysis as the irreversible reduction peak (−0.88 V versus SHE) developed much more slowly over time (Fig. S 45) in the lower pH experiment.

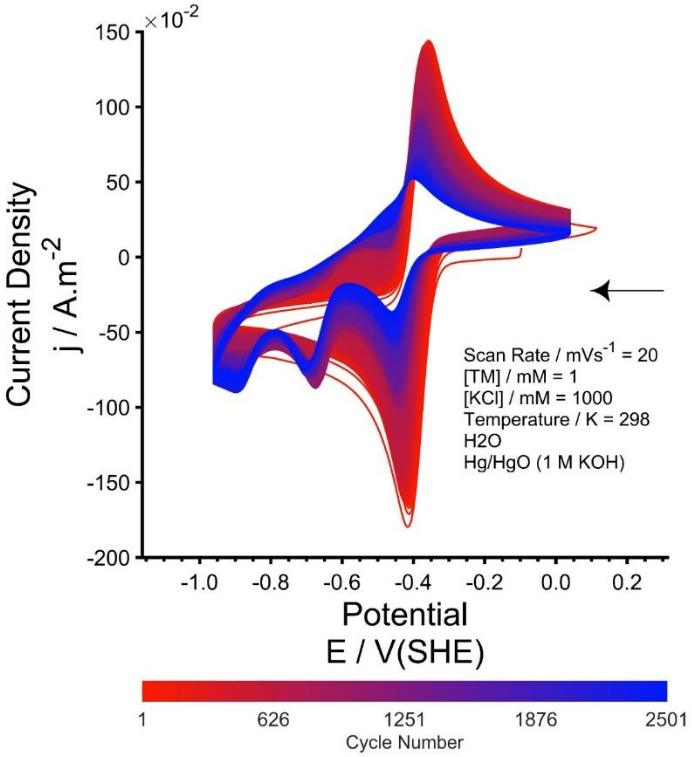

Fig. S 46: CV of FMN at pH 12.3. Long-time (48 h) CV of 1 mM FMN-Na in 1 M KCl with KOH (pH 12.3) in degassed D_2O.

6.3 Cycling a RFB at pH 10.3

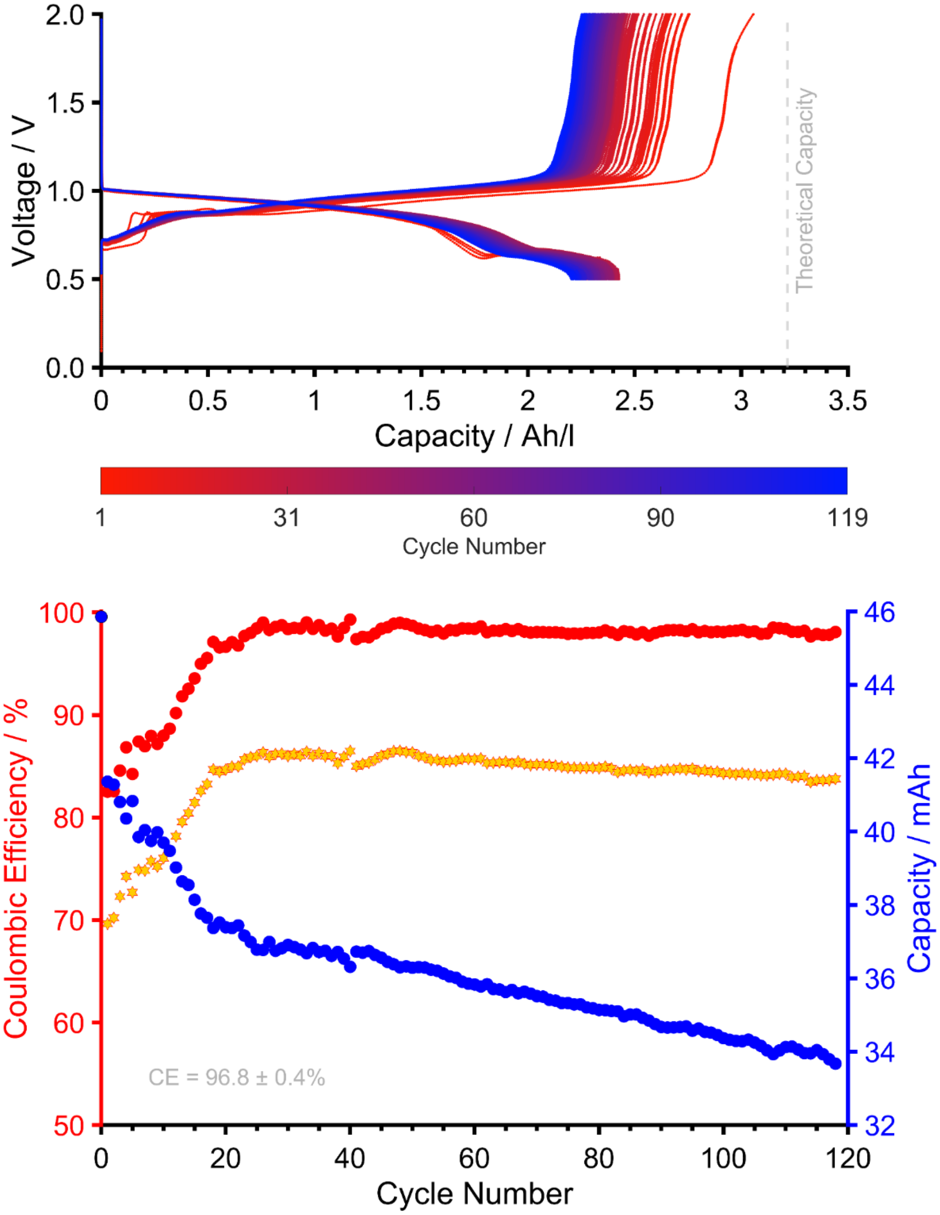

Fig. S 47: Cycling FMN at pH 10.3 Cycling a RFB between 0.5 V and 2.0 V for 119 cycles with 0.2 M $K_4[Fe(CN)_6]$ and 0.05 M $K_3[Fe(CN)_6]$ in 1 M KCl in D_2O as a positive electrolyte and 60 mM FMN^{3-} 1 M KCl in D_2O as a negative electrolyte. The electrolyte was brought to pH 10.3 by the addition of KOH. The top figure shows the charge-discharge profiles at a current density of ±10 mA cm^{-2} (±50 mA) over 119 cycles. The bottom figure shows the cycling discharge capacity and efficiencies of the RFB system.

To examine how the RFB performed at lower pH (Fig. S 47) both electrolyte solutions were adjusted to pH 10.3. A solution of 1 M KCl with the redox active compounds was prepared and KOH was added until the desired pH was reached. The battery was then cycled as previously described (Fig. 1c). Additionally, the membrane was then treated in the same procedure as before but was stored in 0.1 M KCl (cf. 0.1 M KOH). Overall, the battery demonstrated the expected cycling behavior. A charging plateau was observed at around 1.05 V and a discharge plateau at 1.02 V and importantly, the presence of the additional charging plateau at 1.41 V (due to the hydrolysis product) was greatly reduced. This demonstrates the effect that pH has in mitigating hydrolysis and precluding the need for deep discharging. However, the cycling performance also showed a sudden drop in voltage at the beginning of each charge cycle and at the end of each discharge cycle. Precipitate was observed in the anolyte tank during these processes. The change in solubility at this point generates an overpotential results in these undesirable voltage features.

The efficiencies observed in this RFB were unstable until the 40th cycle (Fig. S 47). The Coulombic and Voltaic efficiencies increased while the capacity decreased. After the 40th cycle the efficiencies and capacity fade rate stabilized, with the capacity decreasing slowly at a constant rate. Until the 40th cycle, it is likely that the system was equilibrating via ion exchange across the membrane and between the two solutions.

6.4 In-situ pH analysis of a RFB at pH 14 and pH 10.10

To investigate the cause of the shift towards lower potentials in the CV (Fig. S 45) and the cause for precipitation of FMN^{3-} in the battery performance discussed above, the change in the pH during battery cycling was tracked.

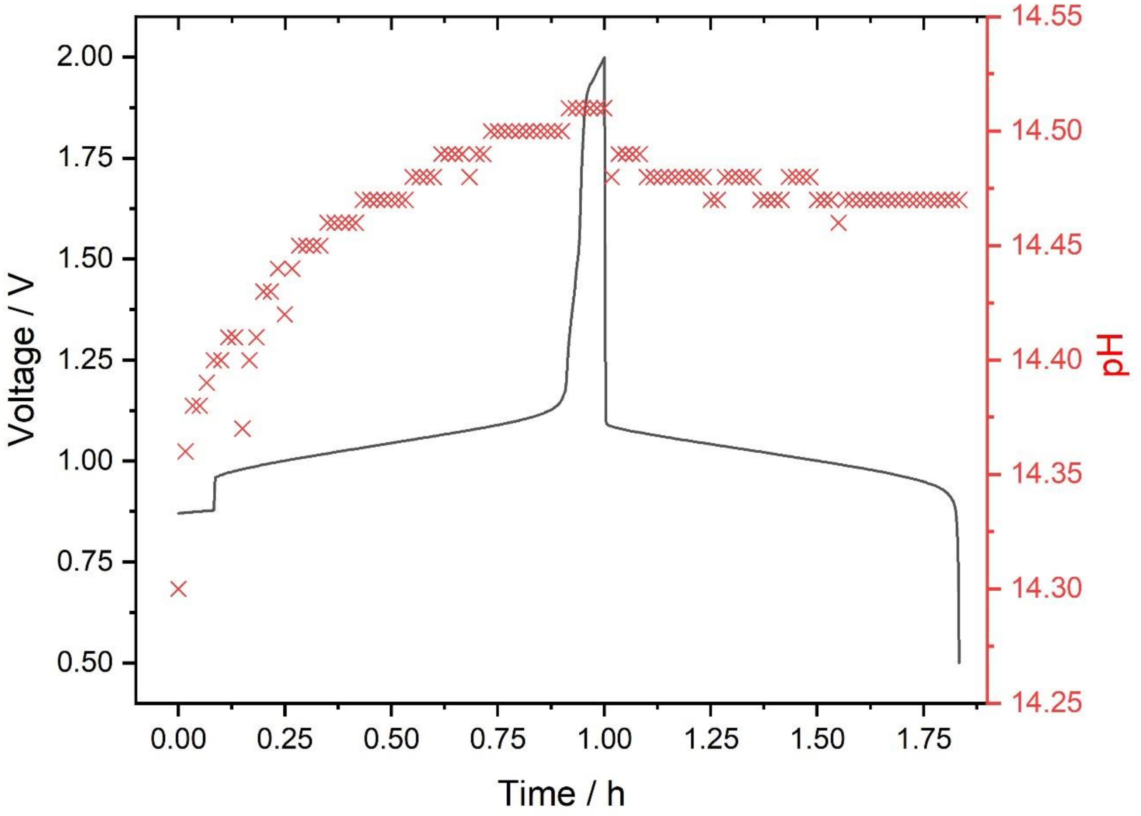

Fig. S 48: pH changes of FMN during cycling at pH 14. Cycling a RFB between 0.5 V and 2.0 V with 0.2 M $K_4[Fe(CN)_6]$ and 0.05 M $K_3[Fe(CN)_6]$ in 1 M KOH in D_2O as a positive electrolyte and 60 mM FMN^{3-} 1 M KOH in D_2O as a negative electrolyte. The voltage profile is shown in black and the corresponding in-situ pH measurements in red.

A RFB consisting of a freshly prepared solution of FMN^{3-} was cycled using the same procedure as previously described (Fig. 1). During the first cycle, the pH of the anolyte tank was recorded every minute (Fig. S 48). Upon application of the current, the voltage initially increased and plateaued at 1.0 V and then increased further to 1.7 V with a sloping plateau rising towards 2.0 V. During discharge, the voltage decreased, plateaued at 1.0 V, and then dropped down further to 0.5 V. Before cycling, the pH was 14.30 (Fig. S 48). Whilst charging the battery, the pH initially increased rapidly before slowing down as it reached 14.51. During discharge, the pH effectively plateaued at 14.47. The pH did not return to 14.30 after a full cycle. The increase in pH after the end of the first cycle may be explained by water reduction, i.e., hydrogen evolution during the plateau at 1.7 V.

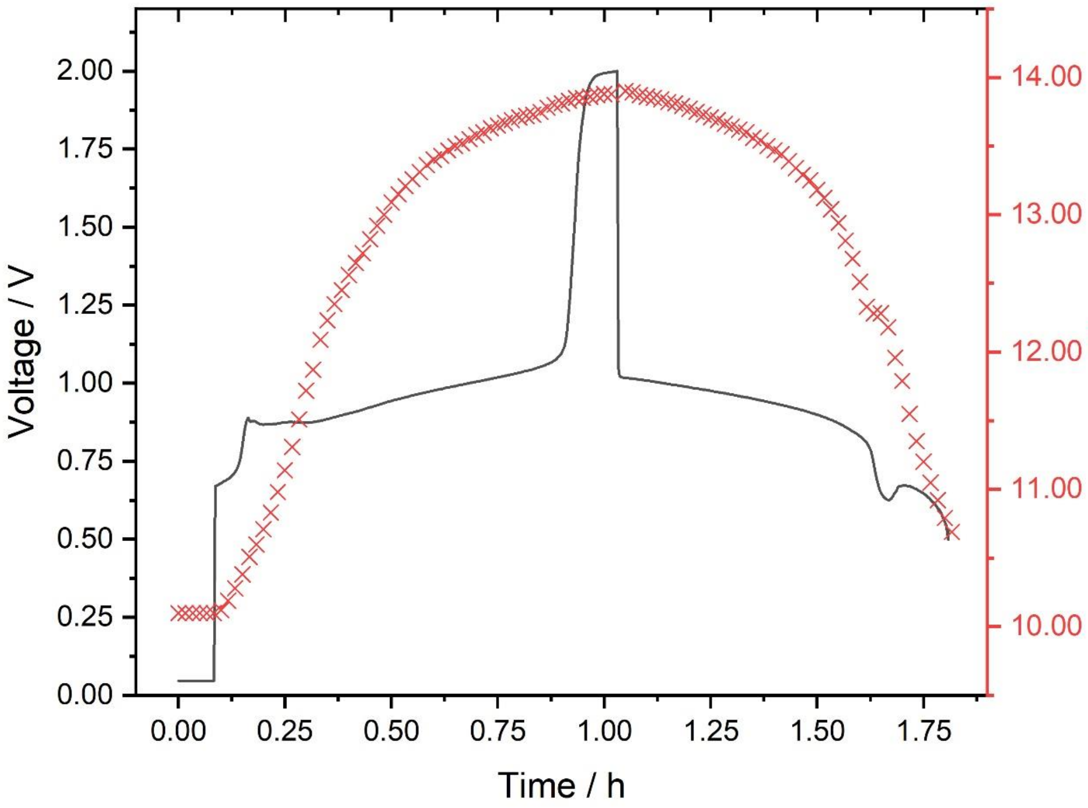

Fig. S 49: pH changes of FMN during cycling at pH 10.3. Cycling a RFB between 0.5 V and 2.0 V with 0.2 M $K_4[Fe(CN)_6]$ and 0.05 M $K_3[Fe(CN)_6]$ in 1 M KCl in D_2O as a positive electrolyte and 60 mM FMN^{3-} 1 M KCl in D_2O as a negative electrolyte. The electrolyte was brought to pH 10.3 by the addition of KOH. The voltage profile is shown in black and the corresponding in-situ pH measurements in red.

A further RFB consisting of a freshly prepared solution of FMN^{3-} at a pH of 10.10 was cycled, as above, and the pH was recorded every minute (Fig. S 49). The same features in the electrochemistry were observed as previously described (Fig. S 47) with a drop in the voltage at the start and end of each charge and discharge cycle, respectively. The changes in pH were more significant for this cycling performance than for the system described above. The pH started at 10.10 before cycling. Under the application of a current, the pH increased significantly and rapidly. As cycling continued, the rate of increase in the pH slowed down, with a pH of 13.50 at the end of cycling. During discharge, the pH initially decreased slowly but as the discharge continued, the rate of pH change increased significantly. At the end of the discharge cycle, the pH was 10.69. A water splitting plateau was also observed in this experiment, which as before may explain the higher pH observed after a complete charge and discharge cycle.

Upon closer inspection of the battery performance with regards to the changes in pH, a clear correlation between the pH and undesirable voltage features can be observed (Fig. S 50 and Fig. S 51). Where there is an overpotential due to precipitation, there is a plateau in the pH. These plateaus correlate well with these voltage features as the system turns turbid from precipitation (Fig. S 52). Formation of a suspension

indicates that the solubility of either the singly or doubly reduced species is lower than that of FMN^{3-}. However, as the charging/discharging progresses the pH also increases enabling dissolution of the precipitate. The re-dissolution of the precipitate indicates that a higher pH than 10 should be used to avoid these detrimental voltage features.

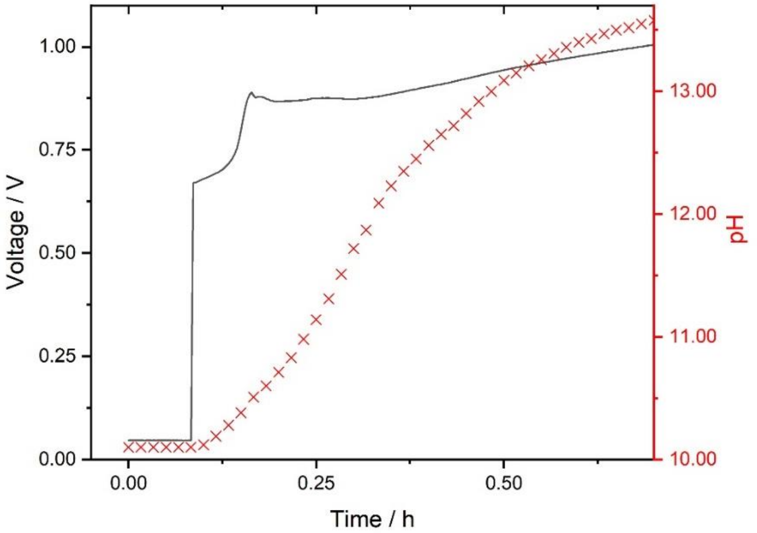

Fig. S 50: pH chnages of FMN during cycling at 10.3 (start of reduction). Cycling a RFB between 0.5 V and 2.0 V with 0.2 M $K_4[Fe(CN)_6]$ and 0.05 M $K_3[Fe(CN)_6]$ in 1 M KCl in D_2O as a positive electrolyte and 60 mM FMN^{3-} 1 M KCl in D_2O as a negative electrolyte. The electrolyte was brought to pH 10.3 by the addition of KOH (c.f. Fig. S 49). The voltage profile is shown in black and the corresponding in-situ pH measurements in red. Expanded view of the start of cycling and pH performance.

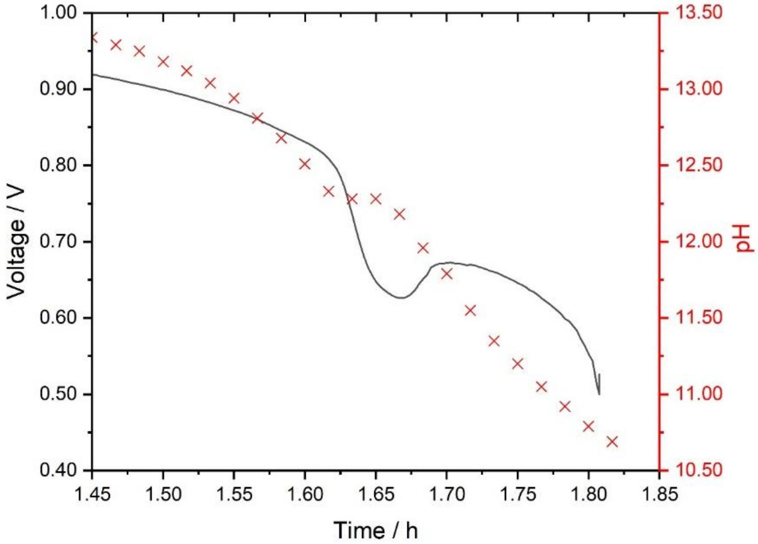

Fig. S 51: pH chnages of FMN during cycling at 10.3 (end of oxidation). Cycling a RFB between 0.5 V and 2.0 V with 0.2 M $K_4[Fe(CN)_6]$ and 0.05 M $K_3[Fe(CN)_6]$ in 1 M KCl in D_2O as a positive electrolyte and 60 mM FMN^{3-} 1 M KCl in D_2O as a negative electrolyte. The electrolyte was brought to pH 10.3 by the addition of KOH (c.f. Fig. S 49). The voltage profile is shown in black and the corresponding in-situ pH measurements in red. Expanded view of the end of the cycling and pH performance.

Whilst measuring the pH during battery operation, a comparison of the concentration of electrons added, (calculated from the current applied to the system), to the concentration of hydroxides generated (calculated from the changes in pH) was carried out (Fig. S 53 and Fig. S 54). At pH 14, the comparison between the concentration of hydroxides being generated and number of electrons does not show any obvious correlation (Fig. S 53). However, a strong correlation between the concentration of electrons and the concentration of hydroxide can be seen when cycling at a pH of 10 (Fig. S 54). The concentration of electrons increases during charging and decreases during discharging linearly as we apply a constant current to the system. The concentration of hydroxide also increases and decreases linearly as the concentration of electrons change. Of note, the change in the concentration of hydroxide is slightly delayed relative to the transfer of electrons. This may be as a result of precipitation of slightly acidic molecules, removing them from solution and therefore changing the pH. Alternatively, the electrons are added through the electrochemical cell and the pH is measured in the tanks. Due to this, the pH changes are likely to be slightly delayed as the solution takes time to travel to the tank from the electrochemical cell and mix. Moreover, it is also possible that the pH meter suffers from some degree of lag or lacks the sensitivity to respond to minimal changes in pH. Nevertheless, we have observed a correlation between the concentration of electrons and concentration of hydroxides whilst cycling this RFB at a pH of 10. The concentration of hydroxides generated by the system is roughly twice the number of electrons added to the system, which is likely to be as a result of the two-electron redox reaction that FMN^{3-} undergoes whilst cycling.

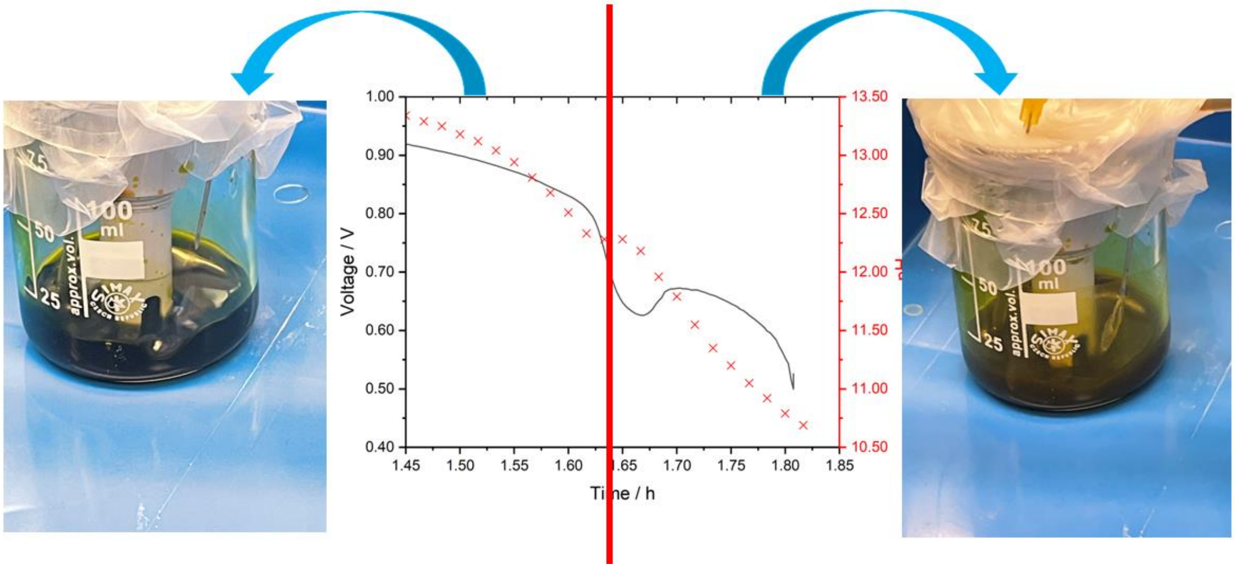

Fig. S 52: Solutions during cycling at 10.3 (end of oxidation). Cycling a RFB between 0.5 V and 2.0 V with 0.2 M $K_4[Fe(CN)_6]$ and 0.05 M $K_3[Fe(CN)_6]$ in 1 M KCl in D_2O as a positive electrolyte and 60 mM FMN^{3-} 1 M KCl in D_2O as a negative electrolyte. The electrolyte was brought to pH 10.3 by the addition of KOH (c.f. Fig. S 49). The voltage profile is shown in black and the corresponding in-situ pH measurements in red. Expanded view of the end of the cycling and pH performance with pictures of the solutions before and after the red line.

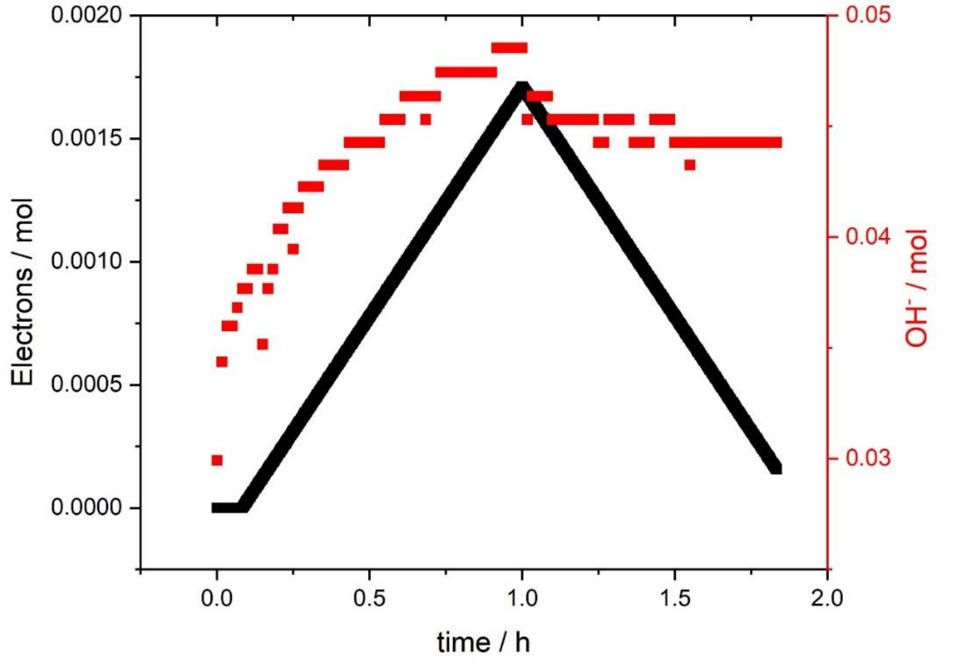

Fig. S 53: Electrons vs pH changes for cycling at pH 14. Cycling a RFB between 0.5 V and 2.0 V with 0.2 M $K_4[Fe(CN)_6]$ and 0.05 M $K_3[Fe(CN)_6]$ in 1 M KOH in D_2O as a positive electrolyte and 60 mM FMN^{3-} 1 M KOH in D_2O as a negative electrolyte (c.f. Fig. S 48). The concentration of electrons calculated from the electrochemistry is shown in black and the corresponding concentration of hydroxide calculated from the pH changed are shown in red.

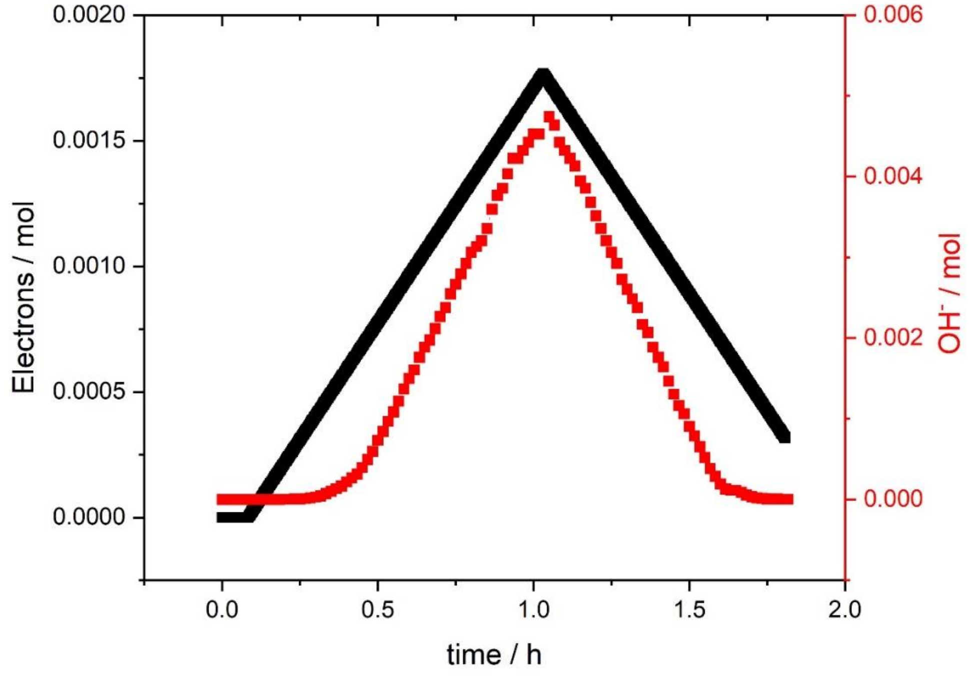

Fig. S 54: Electrons vs pH changes for cycling at pH 10.3. Cycling a RFB between 0.5 V and 2.0 V with 0.2 M $K_4[Fe(CN)_6]$ and 0.05 M $K_3[Fe(CN)_6]$ in 1 M KCl in D_2O as a positive electrolyte and 60 mM FMN^{3-} 1 M KCl in D_2O as a negative electrolyte. The electrolyte was brought to pH 10.3 by the addition of KOH (c.f. Fig. S 49). The concentration of electrons calculated from the

electrochemistry is shown in black and the corresponding concentration of hydroxide calculated from the pH changed are shown in red.

6.5 In-situ ^{1}H and ^{31}P analysis of a RFB at pH 10

The RFB at pH 10 was also analyzed by in-situ ^{1}H NMR (Fig. S 55) and ^{31}P NMR (Fig. S 56). The in-situ ^{1}H NMR (Fig. S 55) shows the same features as the freshly prepared sample at pH 14 (Fig. 2b, c, d), i.e., a disappearance of the signals from FMN^{3-} (H6 and H9, and H7α and H8α) under application of a charging current. During the water splitting plateau (1.71 V) we observe the signals for FMN^{5-} (H6” and H9”, and H7”α and H8”α). Changing the pH of the solution to 10 has, therefore, no effect on the ^{1}H NMR spectra and the overall electrochemistry as the disappearance of the relevant proton signals were still observed. As before, the disappearance can be attributed to the formation of a radical species, in this case $FMN^{4-\bullet}$.

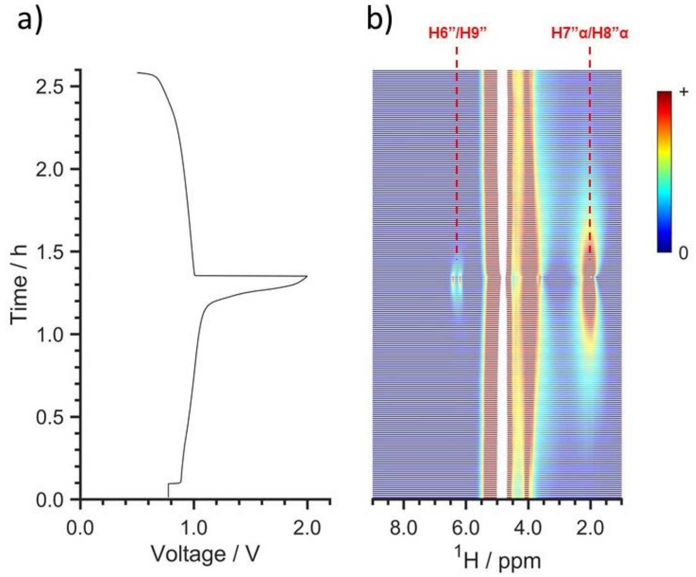

Fig. S 55: in-situ ^{1}H NMR analysis of FMN at pH 10. (left) Voltage of a 60 mM FMN^{3-} versus 0.2 M $K_4[Fe(CN)_6]$ and 0.05 M $K_3[Fe(CN)_6]$ in 1 M KCl/D_2O full cell as a function of time. During charge, a constant current density of 10 mA cm^{-2} (50 mA) was applied until 2.0 V was reached. Turing discharge, a constant current density of –10 mA cm^{-2} (−50 mA) was applied until 0.5 V was reached. The electrolyte was brought to pH 10.3 by the addition of KOH. (right) ^{1}H NMR spectra of the anolyte. The color bar indicates the intensity of resonance in positive arbitrary units. The acquisition time per NMR spectrum is 40 s.

The in-situ ^{31}P NMR (Fig. S 56), on-the-other-hand, demonstrates a slightly different trend to what was observed for the RFB at pH 14 (Fig. S 19 and Fig. S 20). The broadening of the major signal at 4.78 ppm was still observed, but in addition that a signal that changed significantly during cycling was also observed. At the start of the charge cycle, the signal in question shifted significantly towards higher chemical shifts. The rate of change of chemical shift decreased towards the end of the charge cycle as the signal tended towards 5.30 ppm. Conversely, the rate of change of chemical shift was less significant at the beginning of discharge but became more significant towards the end. This signal was most likely the same signal that was previously observed at 5.30 ppm (Fig. S 19 and Fig. S20). The chemical shift for this species is highly pH dependent. As the species buffers the solution, e.g., change in protonation state of a phosphate, there is a corresponding change in that local phosphorus environment. As the system approaches pH 14 at the end of the charge cycle (Fig. S 56), the compound causing this signal has the same chemical environment as that observed in the pH 14 RFB (Fig. S 19 and Fig. S 20), resulting in the same chemical shift. Therefore, this in-situ ^{31}P NMR method could also be used to follow the effect of buffers as demonstrated here.

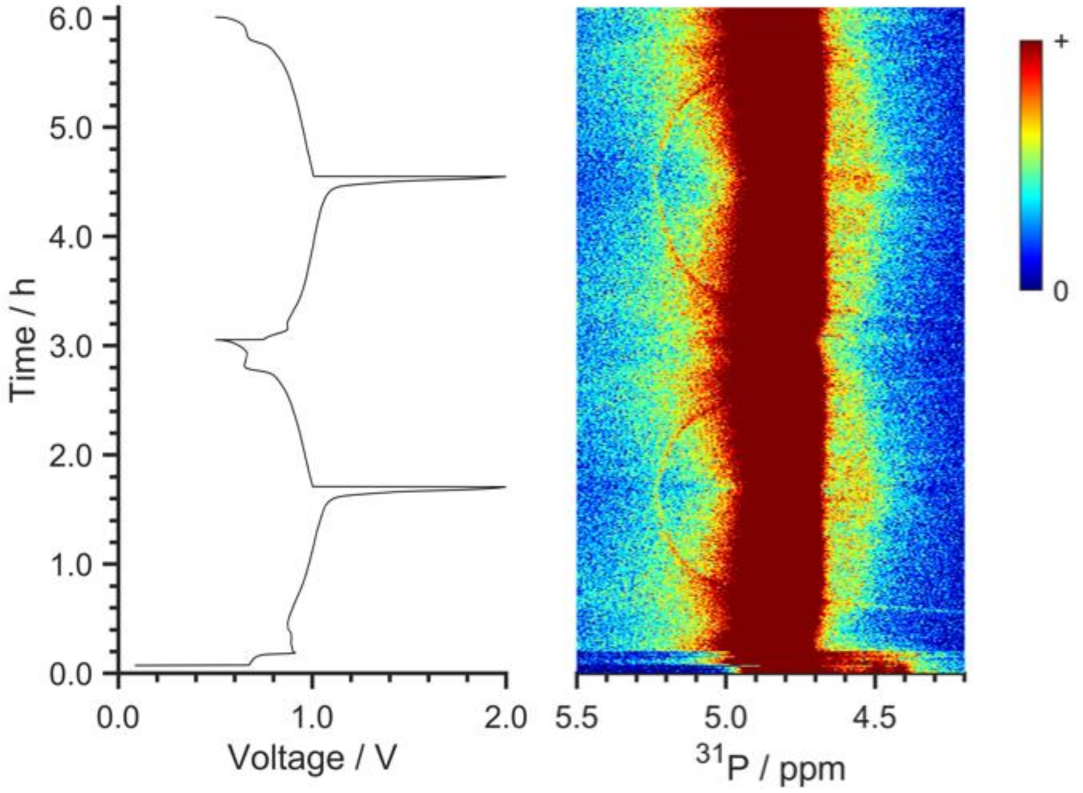

Fig. S 56: in-situ ^{31}P NMR analysis of FMN at pH 10. (left) Voltage of a 60 mM FMN^{3-} versus 0.2 M $K_4[Fe(CN)_6]$ and 0.05 M $K_3[Fe(CN)_6]$ in 1 M KCl/D_2O full cell as a function of time. During charge, a constant current density of 10 mA cm^{-2} (50 mA) was applied until 2.0 V was reached. During discharge, a constant current density of –10 mA cm^{-2} (−50 mA) was applied until 0.5 V was reached. The electrolyte was brought to pH 10.3 by the addition of KOH. (right) ^{31}P NMR spectra of the anolyte. The color bar indicates the intensity of resonance in positive arbitrary units. The acquisition time per NMR spectrum is 40 s.

6.6 Running an RFB with a buffered solution

To avoid the pH changes during cycling, the RFB was run in a buffered solution. The universal Britton-Robinson buffer, which buffers in the range from pH 2 to 12 was used.

First, a Britton-Robinson buffer comprising of 0.3 M Acetic Acid, 0.3 M Boric Acid, and 0.3 M Phosphoric Acid was used. To the solution, KOH was added until pH 12 was reached. After the addition of 60 mM FMN, the pH was 10.8. The RFB was cycled as previously described (Fig S. 12) and followed by ^{1}H NMR (Fig S. 57) and ^{31}P NMR (Fig S. 58). The voltage profile (Fig S. 57 and Fig S. 58) was different to the other battery experiments described in this work. The charge plateau was observed at a lower voltage (~900 mV) and a steady increase in the voltage was not observed. Additionally, the charging process was observed to take longer than the discharging process. The ^{1}H NMR (Fig S. 57) showed a similar behavior to before (Fig. 2b, c, d), with the additional resonances of the buffer. The NMR signals of the buffer were not observed to broaden as the paramagnetic species is only associated with the FMN. The ^{31}P NMR (Fig S. 59) also exhibited the same trends as previously described (Fig S. 16 and Fig S. 17) with an additional peak from the phosphoric acid. As expected, the phosphoric acid peak was significantly shifted (Fig S. 56). The voltage features in this experiment may be explained by both the buffer not being able to maintain the pH of the solution during cycling (Fig S. 59), and by the precipitation of FMN^{3-} from solution.

Another RFB with 1 M Acetic Acid, 1 M Boric Acid, and 1 M Phosphoric Acid was assembled to increase the buffering capability of the system. The battery was cycled between 0.5 V and 1.7 V as described above (Fig S. 59). The first charging plateau was again centered at a lower voltage (~900 mV) and the full theoretical capacity was not achieved (first charge cycle capacity of 0.61 Ah L−1). Under continuous cycling, the battery suffered significant capacity fade due to the precipitation of FMN^{3-}. After 40 cycles, the solute had completely precipitated.

The results presented within this Supplementary Note 5 show that running the FMN^{3-} RFB with a buffer does not have any advantages. Therefore, the pH of the unbuffered system needs to be adjusted to avoid hydrolysis (pH > 12) and avoid loss of FMN^{3-} via precipitation (pH < 10).

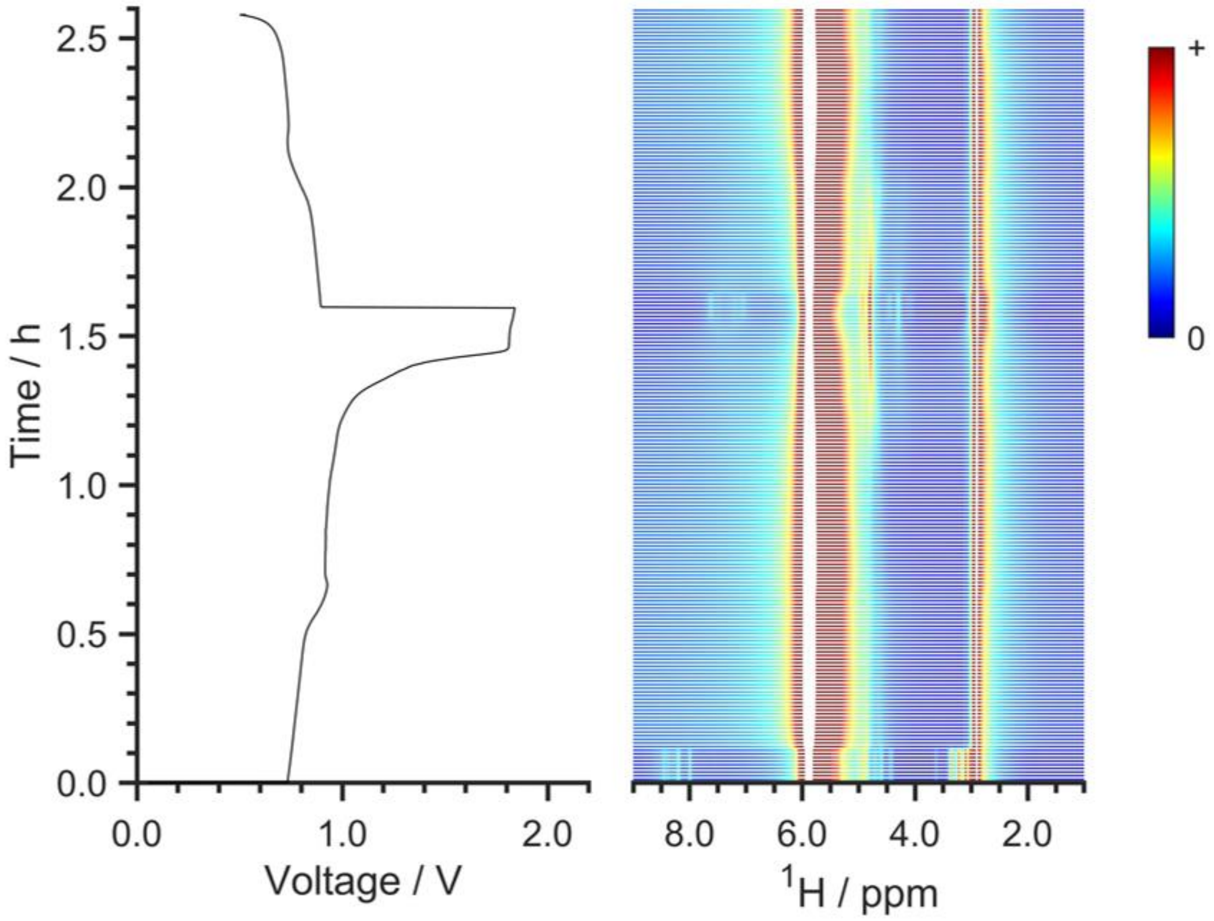

Fig. S 57: in-situ ^{1}H NMR analysis of FMN at pH 12 buffered. (left) Voltage of a 60 mM FMN^{3-} versus 0.2 M $K_4[Fe(CN)_6]$ and 0.05 M $K_3[Fe(CN)_6]$ in 0.3 M Acetic Acid/0.3 M Boric Acid/0.3 M Phosphoric Acid in D_2O full cell as a function of time. During charge, a constant current density of 10 mA cm^{-2} (50 mA) was applied until 2.0 V was reached. During discharge, a constant current density of –10 mA cm^{-2} (−50 mA) was applied until 0.5 V was reached. The buffer was brought to pH 12 by the addition of KOH. The pH of the electrolyte ended up at around 11 after the addition of FMN. (right) 1H NMR spectra of the anolyte. The color bar indicates the intensity of resonance in positive arbitrary units. The acquisition time per NMR spectrum is 40 s.

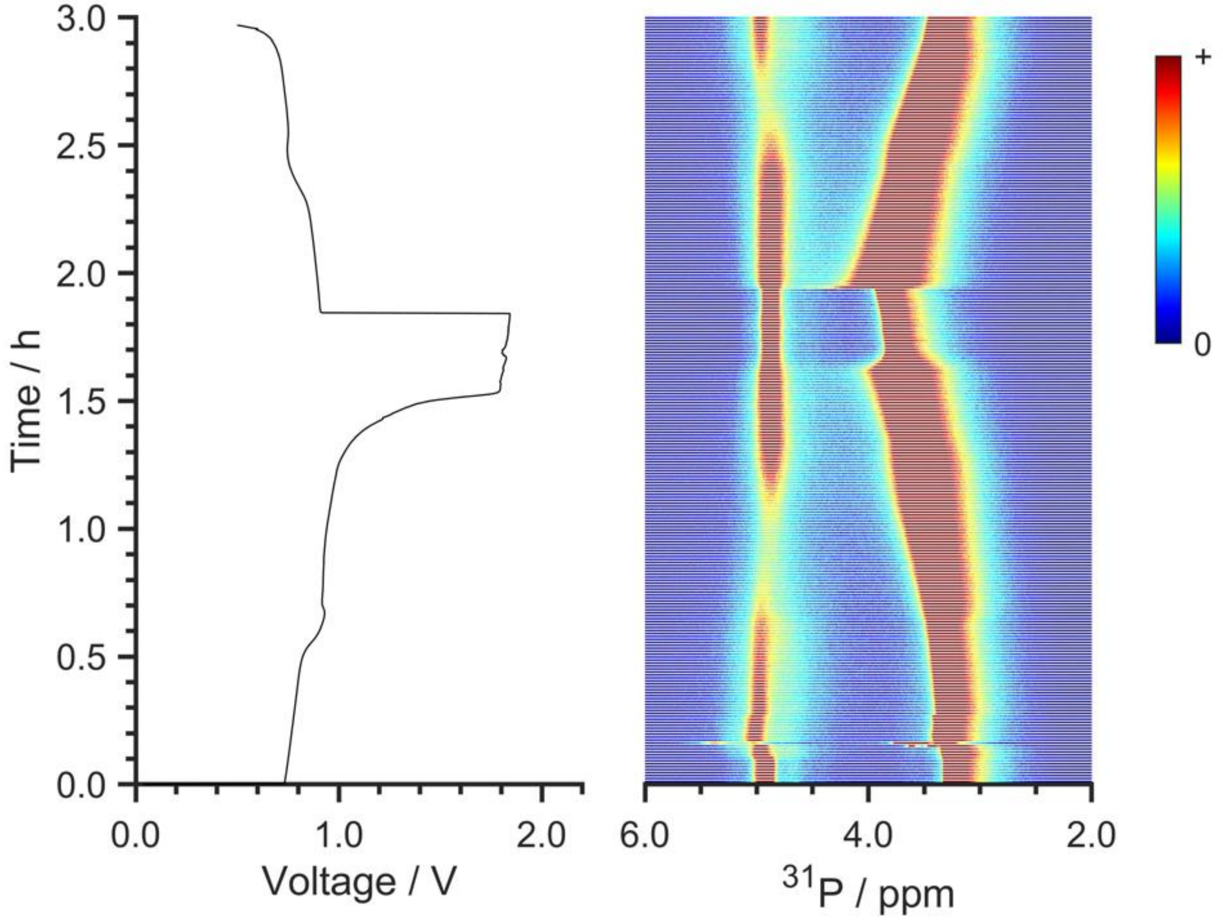

Fig. S 58: in-situ ^{31}P NMR analysis of FMN at pH 12 buffered. (left) Voltage of a 60 mM FMN^{3-} versus 0.2 M $K_4[Fe(CN)_6]$ and 0.05 M $K_3[Fe(CN)_6]$ in 0.3 M Acetic Acid/0.3 M Boric Acid/0.3 M Phosphoric Acid in D2O full cell as a function of time. During charge, a constant current density of 10 mA cm^{-2} (50 mA) was applied until 2.0 V was reached. During discharge, a constant current density of –10 mA cm^{-2} (−50 mA) was applied until 0.5 V was reached. The buffer was brought to pH 12 by the addition of KOH. The pH of the electrolyte ended up at around 11 after the addition of FMN. (right) ^{31}P NMR spectra of the anolyte. The color bar indicates the intensity of resonance in positive arbitrary units. The acquisition time per NMR spectrum is 40 s.

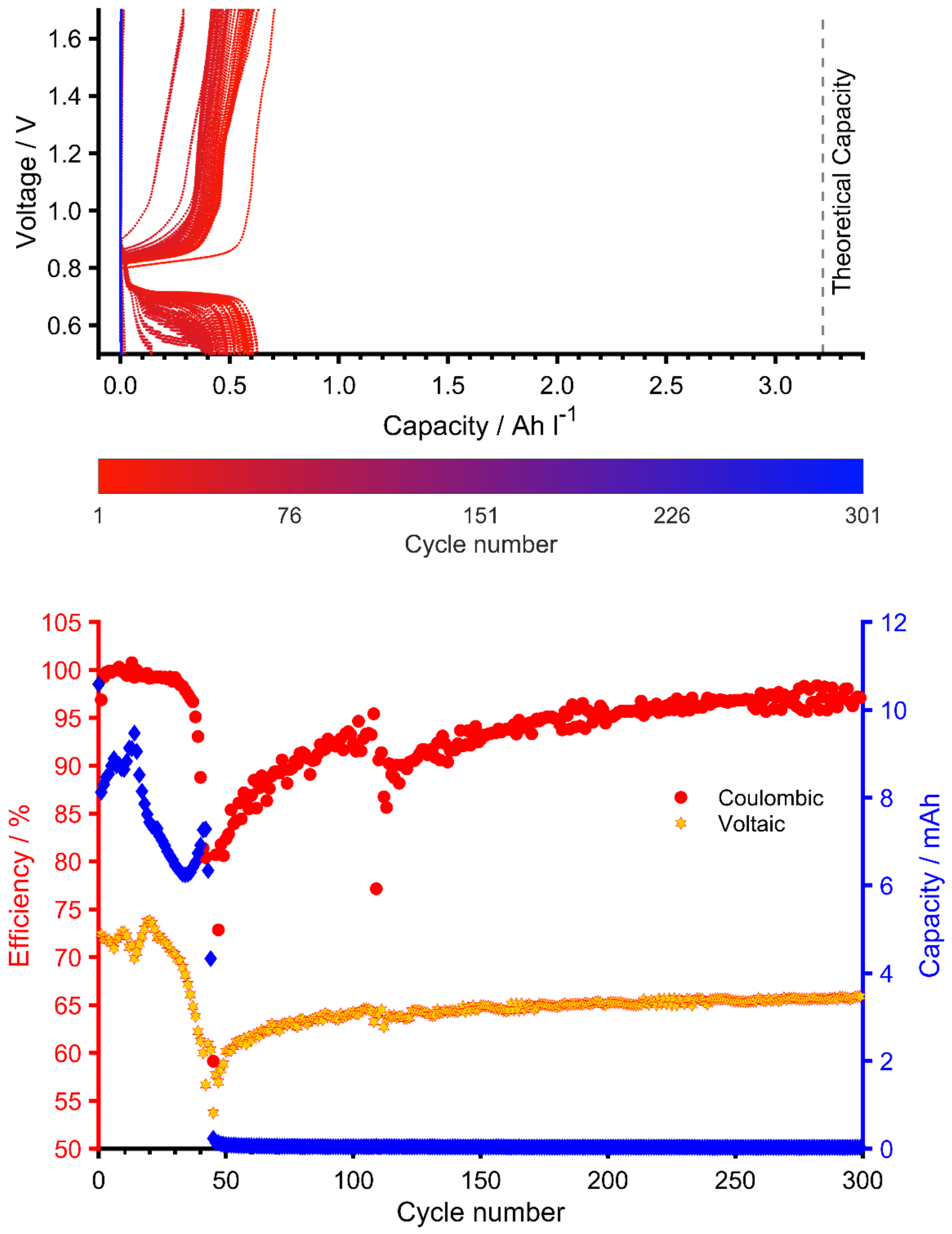

Fig. S 59: Cycling FMN at pH 12 buffered. Cycling a RFB between 0.5 V and 1.7 V for 300 cycles with 0.2 M $K_4[Fe(CN)_6]$ and 0.05 M $K_3[Fe(CN)_6]$ in 1 M Acetic Acid/1 M Boric Acid/1 M Phosphoric Acid in D_2O as a positive electrolyte and 60 mM FMN^{3-} 1 M KCl in D_2O as a negative electrolyte. The buffer was brought to pH 12 by the addition of KOH. The top Figure shows the charge-discharge profiles at a current density of ±10 mA cm^{-2} (±50 mA) over 300 cycles. The bottom figure shows the cycling discharge capacity and efficiencies of the RFB system.

Supplementary Note 7: Flavin mononucleotide RFB at pH 11 and study of oxygen side reactions

7.1 Cycling performance at pH 11.4 unbuffered

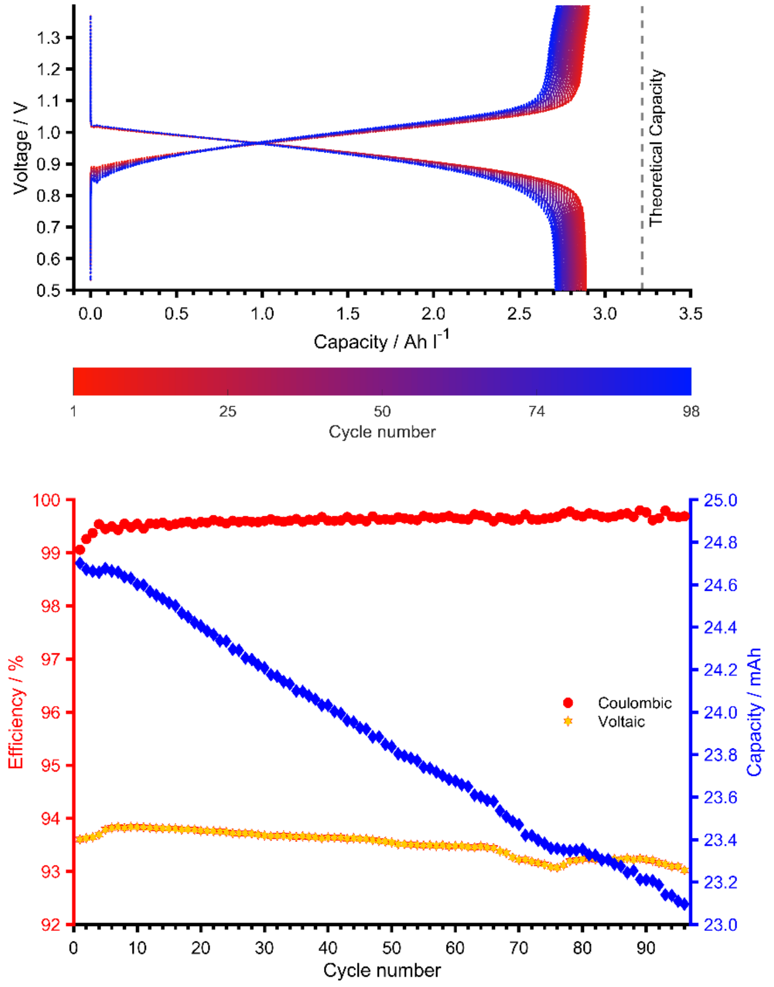

Fig. S 60: Galvanostatic cycling of FMN at pH 11.4. Cycling a RFB between 0.5 V and 1.4 V for 98 cycles with 0.2 M $K_4[Fe(CN)_6]$ and 0.05 M $K_3[Fe(CN)_6]$ in 1 M KCl in D_2O as a positive electrolyte and 60 mM FMN^{3-} 1 M KCl in D_2O as a negative electrolyte. The electrolyte was brought to pH 11.4 by the addition of KOH. Two stacked membranes were used. The top Figure shows the charge-discharge profiles at a current density of ±10 mA cm^{-2} (±50 mA) over 98 cycles. The bottom figure shows the cycling discharge capacity and efficiencies of the RFB system. This graph was plotted without the first cycle due to trace amounts of oxygen during the first cycle. The graph with the first cycle can be seen in Fig S. 61.

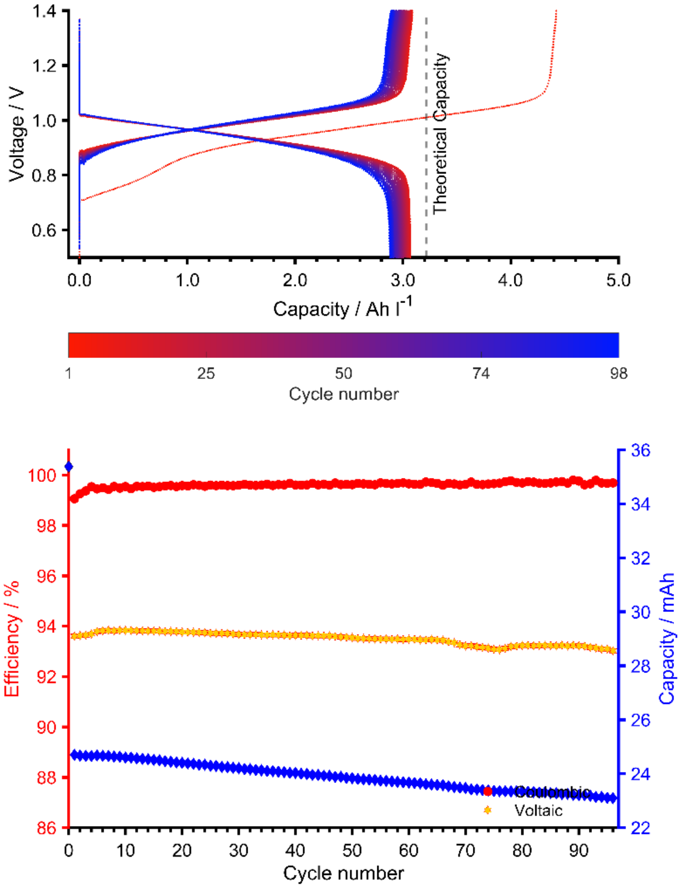

Fig. S 61: Galvanostatic cycling of FMN at pH 11.4 (including first cycle). Cycling a RFB between 0.5 V and 1.4 V for 98 cycles with 0.2 M $K_4[Fe(CN)_6]$ and 0.05 M $K_3[Fe(CN)_6]$ in 1 M KCl in D_2O as a positive electrolyte and 60 mM FMN^{3-} 1 M KCl in D2O as a negative electrolyte. The electrolyte was brought to pH 11.4 by the addition of KOH. Two stacked membranes were used. The top Figure shows the charge-discharge profiles at a current density of ±10 mA cm^{-2} (±50 mA) over 98 cycles. The bottom figure shows the cycling discharge capacity and efficiencies of the RFB system. This graph includes the first cycle.

The cycling performance of FMN^{3-} has been described in the main text (Fig. 4). The full performance and efficiencies are seen in Fig S. 60 and Fig S. 61.

This graph was plotted without the first cycle due to trace amounts of oxygen in the system. During charge the oxygen oxidizes the singly or doubly reduced FMN (i.e., FMN^{4-}, FMN^{5-}), leading to an additional capacity on the first charge cycle. We hypothesize that the oxygen is completely reduced as the battery appears to be stabilized after the first charge cycle.

7.2 Study of oxygen side reactions (pH 14)

To support the theory that the presence of oxygen leads to additional capacity on the first the first charge, a battery at pH 14 was assembled and cycled in the described manner, but with oxygen-free nitrogen used to flush the tanks (Fig S. 62). The first cycle no longer exhibits the overshoot, providing evidence that the first electrochemical cycle is influenced by trace amounts of oxygen.

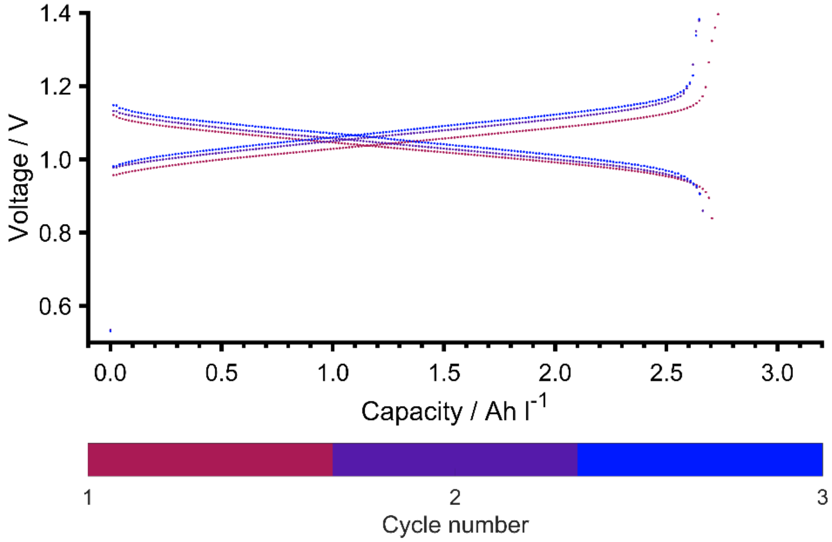

Fig. S 62: Cycling FMN with oxygen free N_2. Cycling a RFB between 0.5 V and 1.4 V for 3 cycles with 0.2 M $K_4[Fe(CN)_6]$ and 0.05 M $K_3[Fe(CN)_6]$ in 1 M KOH in D_2O as a positive electrolyte and 60 mM FMN^{3-} 1 M KOH in D_2O as a negative electrolyte. The battery was cycled with oxygen-free nitrogen flushing the tanks.

7.3 Efficiencies at pH 11.4 unbuffered

Upon comparison of the Coulombic efficiencies, the pH 11 cell shows an overall better current efficiency when compared to the pH 14 cell. After the first 15 cycles, the Coulombic efficiency of the pH 14 cell shows a downward trend over the course of the 90 cycles, drifting away from 100% efficiency. The average Coulombic and voltaic efficiencies for this cell are 98.5% and 85.2%, respectively.

In comparison, the pH 11 cell shows a more stable Coulombic efficiency of close 100% during 98 cycles, with average Coulombic and voltaic efficiencies for the pH 11 cell of 99.8% and 93.5%, respectively. A capacity loss of less than 0.05% per cycle was observed.

7.4 Source of the minor capacity loss at pH 11.4 unbuffered

From Fig S. 60, a capacity loss is evident. This capacity fade may be the result of several factors. The first explanation may be crossover of FMN^{3-}, which is supported by the presence of a ^{31}P NMR resonance in the positive electrolyte as the catholyte does not possess any phosphorus containing species (Fig S. 63). Additionally, the volume of the electrolyte solutions varied over the course of the experiment. It must be noted that a small fraction of the solution may have remained in the tubing or the electrochemical cell. Secondly, FMN molecules may have become trapped in the membrane. This is suggested by the significant discoloration (Fig S. 64) of the membrane after cycling. For future RFB runs, a thicker membrane may prevent these problems and lead to better cycling performance.

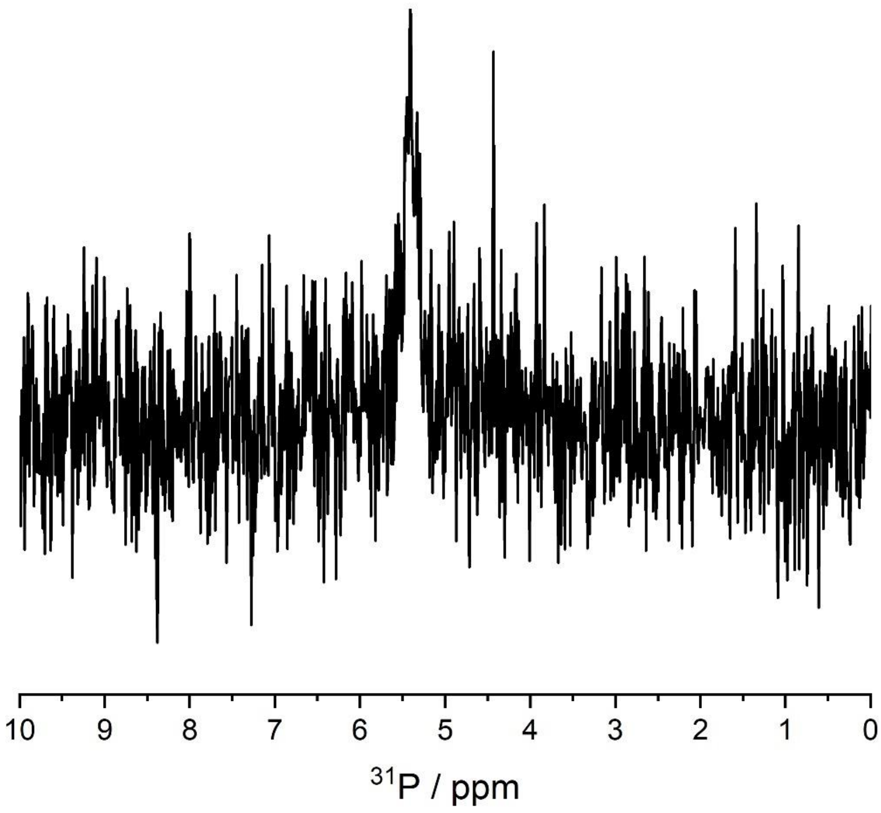

Fig. S 63: ^{31}P NMR of catholyte after cycling. ^{31}P NMR of the positive electrolyte (0.2 M $K_4[Fe(CN)_6]$ and 0.05 M $K_3[Fe(CN)_6]$ in 1 M KCl in D_2O) after cycling the RFB (Fig S. 60) for 98 cycles.

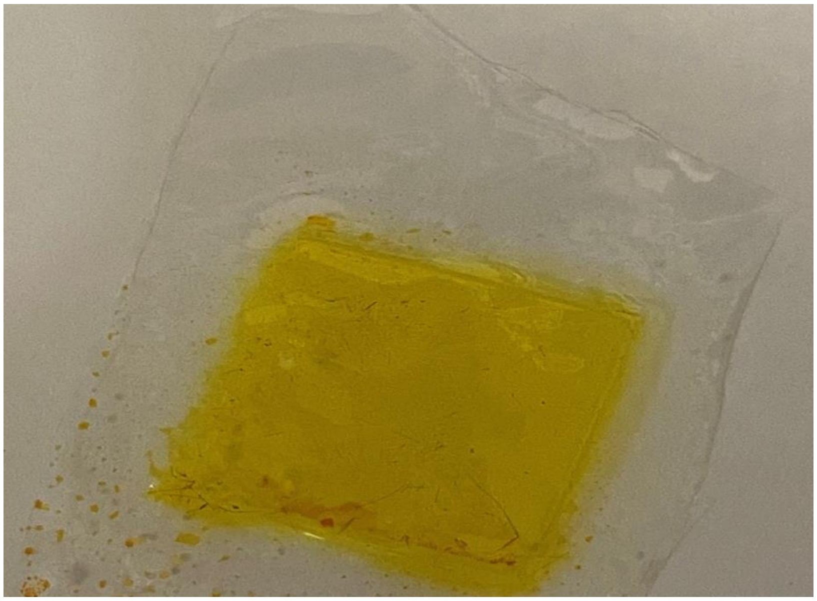

Fig. S 64: Membrane of RFB (Fig. S 60) after cycling 98 cycles.

7.5 Maximum solubility of flavin mononucleotide

The maximum solubility was determined by adding FMN and KOH to the solution until the solution became too viscous to determine whether everything had dissolved. The KOH was added so that the pH stayed at 11.4. The final concentration was approximately ~1.6 M FMN. To prove that everything had dissolved, a sample under an optical microscope was photographed to demonstrate that it was a homogenous solution (Fig S. 65). The cycling performance at higher concentrations does not change. A battery run at pH 11 with 240 mM FMN solution can be seen in Fig S. 66.

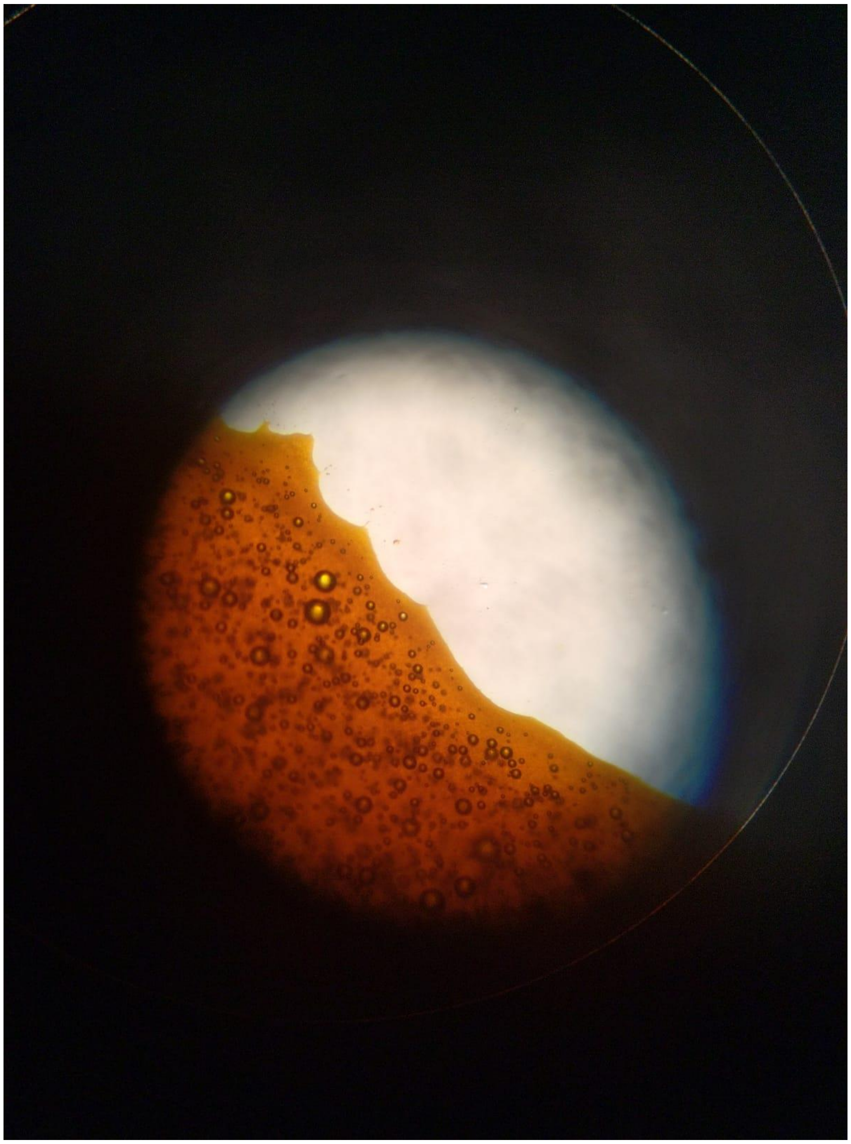

Fig. S 65: Picture of a highly concentrated FMN solution. Solution under a microscope of 1.8 M FMN^{3-} in 1 M KCl/D_2O. KOH was added to reach a pH of 11.4.

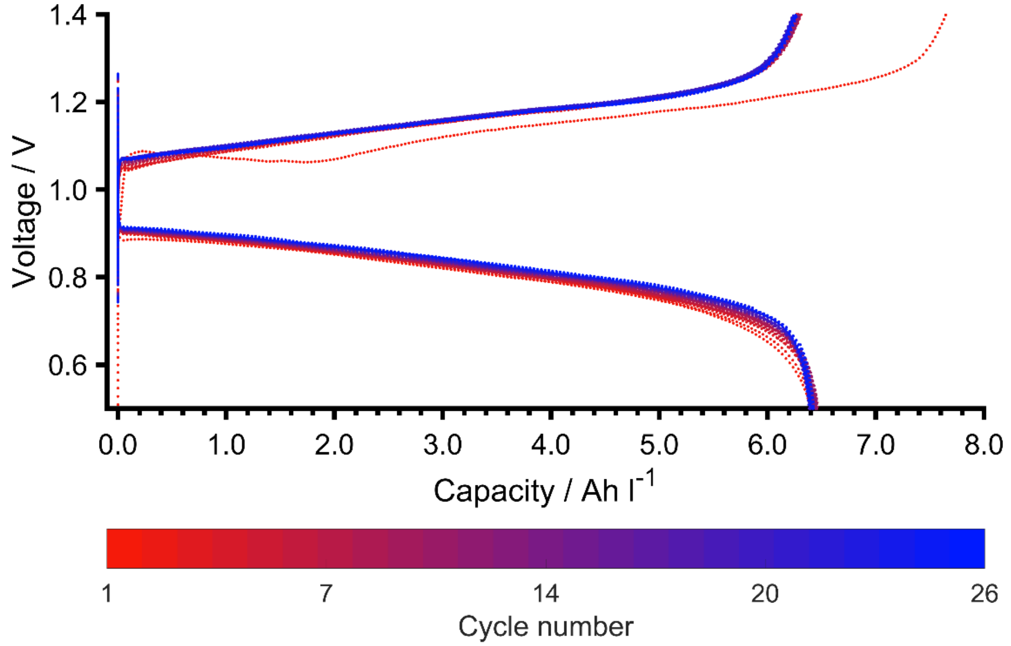

Fig. S 66: Cycling of a highly concentrated FMN solution. Cycling a RFB between 0.5 V and 1.4 V for 26 cycles with 0.2 M $K_4[Fe(CN)6]$ and 0.05 M $K_3[Fe(CN)_6]$ in 1 M KCl in D_2O (80mL) as a positive electrolyte and 240 mM FMN^{3-} 1 M KCl in D_2O (17.5 mL) as a negative electrolyte. The electrolyte was raised to pH 11.4 by the addition of KOH. The figure shows the charge-discharge profiles at a current density of 50 mA cm^{-2} (±250 mA) over 25 cycles.

Table S 1: Coordinates for FMN^{3-}.

ELEMENT	X	Y	Z
C	-2.997339	3.363439	-0.322523
C	-2.232919	2.228794	-0.540766
C	-2.745910	0.944409	-0.308055
C	-4.067759	0.825695	0.185544
C	-4.829702	1.988568	0.402186
C	-4.331216	3.248871	0.153769
C	-3.929553	-1.462377	0.279308
C	-2.568359	-1.452032	-0.233842
C	-2.456363	-3.768561	-0.113521
C	-4.512259	-2.821856	0.577249
H	-1.208634	2.349164	-0.854222
H	-5.836875	1.854095	0.777629
C	-5.171161	4.474729	0.393384
H	-4.711085	5.134536	1.133867
H	-5.290734	5.060844	-0.521697
H	-6.163648	4.204579	0.753167
C	-2.402805	4.721111	-0.579924
H	-2.982027	5.267785	-1.328859
H	-2.407287	5.330776	0.327530
H	-1.375427	4.645209	-0.933945
N	-4.632057	-0.389751	0.466459
N	-2.028743	-0.219719	-0.542882
N	-1.863909	-2.529812	-0.421903
N	-3.734983	-3.894657	0.363774
O	-5.678567	-2.901837	1.005283
O	-1.757920	-4.782263	-0.292026
C	-0.689906	-0.197165	-1.152187
H	-0.612626	-1.071146	-1.788870
H	-0.602142	0.692425	-1.769975
C	0.428315	-0.236630	-0.115023
H	0.324962	-1.159141	0.467455
C	1.806316	-0.235260	-0.800440
H	1.941991	0.745151	-1.273113
C	2.951702	-0.482430	0.189058
H	2.825142	-1.489579	0.607101
C	4.329499	-0.375945	-0.459462
H	4.394307	0.570935	-1.010787
H	4.482742	-1.203019	-1.161521
O	5.281815	-0.407515	0.587007
P	6.829248	0.347428	0.284940
O	7.545481	0.074892	1.624817

**O**	6.479525	1.830162	0.009792
**O**	7.392609	-0.412488	-0.940094
**O**	0.279804	0.905206	0.728181
**H**	1.092778	0.972793	1.256497
**O**	1.803682	-1.278297	-1.782904
**H**	2.472647	-1.084291	-2.448363
**O**	2.875523	0.484998	1.246808
**H**	3.775931	0.522016	1.612416

Table S 2: Coordinates for protonated $FMN^{4-•}$.

ELEMENT	X	Y	Z
C	-2.972297	3.382267	-0.295955
C	-2.217072	2.227960	-0.484358
C	-2.744346	0.948943	-0.285543
C	-4.084524	0.859335	0.152883
C	-4.843416	2.015888	0.345459
C	-4.315452	3.278604	0.120644
C	-3.885280	-1.525438	0.244817
C	-2.555558	-1.474589	-0.216845
C	-2.429247	-3.791637	-0.093034
C	-4.483581	-2.820644	0.539248
H	-1.178194	2.332574	-0.752458
H	-5.869292	1.908056	0.678979
C	-5.158002	4.508069	0.333037
H	-4.724507	5.160548	1.096000
H	-5.234033	5.101038	-0.582504
H	-6.167484	4.245490	0.649089
C	-2.344690	4.732037	-0.525942
H	-2.876825	5.291136	-1.300755
H	-2.373763	5.344393	0.379679
H	-1.303977	4.636858	-0.834967
N	-4.616090	-0.389954	0.392360
N	-2.008749	-0.227377	-0.513579
N	-1.833393	-2.559416	-0.385380
N	-3.721410	-3.913479	0.357728
O	-5.677693	-2.853581	0.944449
O	-1.731215	-4.818338	-0.254502
C	-0.675285	-0.194999	-1.120213
H	-0.587305	-1.068924	-1.756337
H	-0.592962	0.692739	-1.743932
C	0.455386	-0.223460	-0.094802
H	0.354393	-1.139487	0.498557
C	1.829590	-0.232641	-0.788306
H	1.965287	0.742507	-1.271990
C	2.981812	-0.474327	0.195154
H	2.853799	-1.476967	0.623638
C	4.356110	-0.380686	-0.462987
H	4.422892	0.561691	-1.021675
H	4.500870	-1.213879	-1.159594
O	5.315137	-0.410125	0.577828
P	6.859817	0.345481	0.266498
O	7.583403	0.075510	1.603004

**O**	6.507688	1.827652	-0.008901
**O**	7.417553	-0.415553	-0.960443
**O**	0.321066	0.927489	0.740516
**H**	1.138149	0.993687	1.262135
**O**	1.820663	-1.285675	-1.761049
**H**	2.489795	-1.101021	-2.428913
**O**	2.919054	0.502954	1.244950
**H**	3.821896	0.535817	1.604834
**H**	-5.568649	-0.507644	0.718495

Table S 3: Coordinates for deprotonated $FMN^{4-\bullet}$.

ELEMENT	X	Y	Z
C	-3.153985	3.347879	-0.280799
C	-2.344202	2.219938	-0.443594
C	-2.823743	0.922561	-0.258015
C	-4.177844	0.735828	0.152414
C	-4.974958	1.891955	0.308179
C	-4.500448	3.178249	0.096742
C	-3.913686	-1.567292	0.245275
C	-2.569950	-1.487676	-0.193322
C	-2.313059	-3.790703	-0.130592
C	-4.417840	-2.917882	0.523598
H	-1.302715	2.370262	-0.683046
H	-6.004415	1.735500	0.612074
C	-5.406670	4.369695	0.280182
H	-5.030768	5.046231	1.053689
H	-5.487189	4.959274	-0.638028
H	-6.411782	4.058936	0.567597
C	-2.577954	4.724494	-0.494046
H	-3.107253	5.266753	-1.284047
H	-2.652981	5.339219	0.408634
H	-1.525287	4.672584	-0.774628
N	-4.721805	-0.485213	0.401915
N	-2.038021	-0.221523	-0.464049
N	-1.779693	-2.535126	-0.378890
N	-3.588994	-3.974999	0.318264
O	-5.597471	-3.097046	0.941140
O	-1.568408	-4.794324	-0.329057
C	-0.705468	-0.140965	-1.058436
H	-0.579999	-1.006453	-1.699818
H	-0.645336	0.756198	-1.673401
C	0.428178	-0.138334	-0.034999
H	0.343351	-1.050819	0.566217
C	1.804805	-0.133482	-0.724692
H	1.919191	0.826434	-1.243734
C	2.962922	-0.307579	0.266964
H	2.838089	-1.276921	0.767367
C	4.335258	-0.263370	-0.401219
H	4.398269	0.620938	-1.048966
H	4.488100	-1.158574	-1.014030
O	5.292481	-0.182159	0.637954
P	6.907733	0.320937	0.207419
O	7.579999	0.339151	1.597107

**O**	6.713677	1.706657	-0.454327
**O**	7.407363	-0.783196	-0.755638
**O**	0.278605	1.017578	0.793741
**H**	1.102983	1.115688	1.297784
**O**	1.827251	-1.221887	-1.658364
**H**	2.500782	-1.048117	-2.324672
**O**	2.906629	0.742766	1.244833
**H**	3.811390	0.796635	1.598100

Table S 4: Coordinates for protonated FMN^{5-}.

ELEMENT	X	Y	Z
C	3.043052	3.401072	0.412579
C	2.264723	2.242693	0.515673
C	2.747191	0.975496	0.191925
C	4.063206	0.883698	-0.308548
C	4.844419	2.033390	-0.393009
C	4.362568	3.295433	-0.040139
C	3.868539	-1.514395	-0.348112
C	2.565383	-1.454435	0.167579
C	2.460881	-3.784055	0.331144
C	4.384683	-2.788682	-0.474420
H	1.238160	2.350552	0.828788
H	5.858279	1.936345	-0.767989
C	5.247611	4.509661	-0.157998
H	4.826164	5.247906	-0.846749
H	5.367307	5.013588	0.805665
H	6.240458	4.242366	-0.521300
C	2.451987	4.739232	0.779740
H	3.009016	5.216570	1.591775
H	2.472292	5.435019	-0.064559
H	1.415325	4.637692	1.102668
N	4.548054	-0.344142	-0.769274
N	1.966331	-0.203799	0.343651
N	1.877843	-2.540531	0.490318
N	3.742020	-3.903023	-0.156532
O	5.658133	-2.903221	-0.965110
O	1.807392	-4.814069	0.638911
C	0.645888	-0.162403	0.968835
H	0.540502	-1.054615	1.575071
H	0.577547	0.703675	1.625517
C	-0.496842	-0.131295	-0.041707
H	-0.396553	-1.007496	-0.695825
C	-1.866687	-0.204158	0.657458
H	-1.996109	0.713565	1.244638
C	-3.033090	-0.335886	-0.330942
H	-2.897570	-1.269724	-0.892359
C	-4.396983	-0.357683	0.355290
H	-4.467398	0.488854	1.050657
H	-4.523306	-1.287669	0.920372
O	-5.370640	-0.240006	-0.664884
P	-6.973855	0.265148	-0.190924
O	-7.673934	0.314161	-1.565934

**O**	-6.755818	1.636174	0.493599
**O**	-7.461424	-0.854050	0.761058
**O**	-0.381003	1.071246	-0.805583
**H**	-1.214041	1.182998	-1.292474
**O**	-1.852069	-1.356548	1.510956
**H**	-2.523693	-1.250098	2.193041
**O**	-3.007990	0.774399	-1.241249
**H**	-3.917438	0.832593	-1.581462
**H**	5.865258	-3.849186	-0.975466
**H**	5.553569	-0.430467	-0.799479

Table S 5: Coordinates for deprotonated FMN^{5-}.

ELEMENT	X	Y	Z
C	-3.334293	3.337750	-0.058349
C	-2.464821	2.218147	-0.093136
C	-2.914491	0.908887	-0.123817
C	-4.328921	0.620081	-0.027338
C	-5.175262	1.770059	-0.030931
C	-4.708249	3.089947	-0.035152
C	-3.908562	-1.679993	0.089889
C	-2.538153	-1.508616	-0.087387
C	-2.096437	-3.780211	0.060894
C	-4.336945	-3.036092	0.336770
H	-1.397826	2.397901	-0.088537
H	-6.246324	1.585342	-0.004847
C	-5.693409	4.236454	-0.014883
H	-5.559490	4.870980	0.867989
H	-5.573824	4.891457	-0.884660
H	-6.723518	3.875060	-0.009945
C	-2.771930	4.738027	-0.029511
H	-3.116096	5.350975	-0.872338
H	-3.050493	5.288819	0.878299
H	-1.680275	4.719502	-0.070784
N	-4.838036	-0.616644	0.057243
N	-2.028329	-0.197764	-0.260152
N	-1.631367	-2.500619	-0.109701
N	-3.406849	-4.046248	0.308695
O	-5.562895	-3.330830	0.569171
O	-1.253855	-4.748286	-0.008841
C	-0.725629	-0.038846	-0.885877
H	-0.574932	-0.860756	-1.581975
H	-0.718716	0.897322	-1.450481
C	0.463155	-0.039354	0.077950
H	0.400238	-0.953698	0.676653
C	1.811800	-0.030948	-0.667616
H	1.916610	0.940503	-1.167325
C	3.007874	-0.244124	0.271482
H	2.888994	-1.225591	0.749464
C	4.353432	-0.200563	-0.449200
H	4.406294	0.702799	-1.070713
H	4.466692	-1.078178	-1.095029
O	5.354398	-0.168450	0.551939
P	6.949162	0.357749	0.078307
O	7.675665	0.328729	1.440472

**O**	6.724577	1.767210	-0.521310
**O**	7.415403	-0.705100	-0.946232
**O**	0.369199	1.114232	0.924776
**H**	1.211271	1.184914	1.402192
**O**	1.797127	-1.095392	-1.631263
**H**	2.454595	-0.908675	-2.309910
**O**	3.009031	0.777593	1.281384
**H**	3.927277	0.802734	1.601239

Table S 6: Coordinates for RQC^{3-}.

ELEMENT	X	Y	Z
C	-3.321605	3.001777	-0.251730
C	-2.480695	1.926487	-0.498883
C	-2.905007	0.605271	-0.304946
C	-4.209144	0.377016	0.179363
C	-5.049163	1.473788	0.424803
C	-4.640516	2.776748	0.213359
C	-3.911981	-1.905760	0.217626
C	-2.537584	-1.791014	-0.309901
C	-4.420138	-3.315625	0.512435
H	-1.466359	2.124321	-0.807208
H	-6.045795	1.260610	0.792823
C	-5.569968	3.930358	0.483905
H	-5.162116	4.602091	1.244243
H	-5.731057	4.531892	-0.414881
H	-6.540858	3.578049	0.831944
C	-2.822642	4.405725	-0.468882
H	-3.435210	4.933291	-1.205102
H	-2.868757	4.989266	0.454645
H	-1.791600	4.410687	-0.821287
N	-4.686760	-0.894773	0.430708
N	-2.102607	-0.506304	-0.574424
O	-4.184713	-3.741576	1.666518
C	-0.770129	-0.380782	-1.183539
H	-0.642861	-1.218531	-1.860890
H	-0.730439	0.537549	-1.763150
C	0.350835	-0.403097	-0.149477
H	0.280388	-1.340820	0.414969
C	1.729996	-0.343896	-0.830661
H	1.834581	0.647511	-1.287907
C	2.879996	-0.568967	0.158897
H	2.780429	-1.583403	0.566728
C	4.256244	-0.419797	-0.483258
H	4.301232	0.537080	-1.019365
H	4.431029	-1.231719	-1.197813
O	5.204568	-0.446249	0.566817
P	6.751607	0.310330	0.269507
O	7.461604	0.045244	1.614259
O	6.400397	1.791060	-0.014114
O	7.323446	-0.454224	-0.948774
O	0.163400	0.714997	0.716861
H	0.973610	0.800872	1.246712

**O**	1.765272	-1.371895	-1.828438
**H**	2.427730	-1.144688	-2.489886
**O**	2.774579	0.385525	1.226024
**H**	3.670550	0.437616	1.600256
**O**	-1.824718	-2.776114	-0.521454
**O**	-5.021838	-3.887826	-0.424711

Table S 7: Coordinates for protonated $RQC^{4-\bullet}$.

ELEMENT	X	Y	Z
C	-3.469466	2.997197	-0.225871
C	-2.578132	1.942678	-0.435902
C	-2.957241	0.611070	-0.280456
C	-4.281361	0.303673	0.145442
C	-5.166519	1.389068	0.346660
C	-4.794872	2.710630	0.165915
C	-3.832037	-1.980852	0.180541
C	-2.493337	-1.773728	-0.291286
C	-4.305093	-3.336009	0.465123
H	-1.556082	2.178906	-0.690326
H	-6.174822	1.143979	0.661307
C	-5.785449	3.823636	0.396172
H	-5.447575	4.504486	1.182908
H	-5.923575	4.429346	-0.504165
H	-6.759165	3.429763	0.688731
C	-3.008260	4.420139	-0.406496
H	-3.593639	4.939421	-1.171649
H	-3.117013	4.999728	0.515469
H	-1.960112	4.460621	-0.704406
N	-4.717447	-0.958238	0.371677
N	-2.090796	-0.469646	-0.534849
O	-5.430194	-3.612424	0.877803
C	-0.764495	-0.281306	-1.129264
H	-0.588713	-1.102520	-1.815765
H	-0.761443	0.645346	-1.699378
C	0.360775	-0.258301	-0.098435
H	0.299325	-1.179544	0.493520
C	1.742538	-0.212300	-0.776860
H	1.836858	0.751619	-1.291832
C	2.891931	-0.360878	0.228317
H	2.781919	-1.332875	0.727088
C	4.272067	-0.289111	-0.419109
H	4.332989	0.603165	-1.056404
H	4.446377	-1.175135	-1.039582
O	5.209556	-0.207312	0.637306
P	6.840446	0.264870	0.234049
O	7.486560	0.283290	1.635986
O	6.682615	1.647789	-0.442784
O	7.339126	-0.855622	-0.710413
O	0.166549	0.883498	0.737614
H	0.983587	1.005758	1.249084

**O**	1.800596	-1.298001	-1.711546
**H**	2.464303	-1.099924	-2.380954
**O**	2.797260	0.688031	1.204815
**H**	3.690917	0.753909	1.582593
**O**	-1.665740	-2.708943	-0.507901
**O**	-3.425448	-4.350065	0.252033
**H**	-2.576456	-3.929487	-0.085021

Table S 8: Coordinates for deprotonated RQC$^{4–•}$.

ELEMENT	X	Y	Z
C	-3.400335	3.012585	-0.164744
C	-2.518737	1.930904	-0.313155
C	-2.939481	0.604998	-0.216868
C	-4.304852	0.325259	0.093664
C	-5.173849	1.433084	0.232608
C	-4.754886	2.753297	0.106036
C	-3.904560	-1.974464	0.154977
C	-2.545884	-1.827858	-0.220039
C	-4.473915	-3.359209	0.413665
H	-1.471670	2.141365	-0.474808
H	-6.213763	1.213574	0.452305
C	-5.739772	3.885508	0.266530
H	-5.461387	4.549875	1.090620
H	-5.788686	4.508553	-0.631998
H	-6.743919	3.509025	0.466679
C	-2.886305	4.425638	-0.282753
H	-3.383827	4.975822	-1.088663
H	-3.055026	5.000929	0.633847
H	-1.814726	4.438484	-0.487918
N	-4.781342	-0.934742	0.260762
N	-2.089818	-0.492767	-0.426137
O	-3.943691	-4.058044	1.323552
C	-0.768090	-0.332667	-1.019857
H	-0.608032	-1.161437	-1.702761
H	-0.742411	0.594770	-1.592830
C	0.375906	-0.332029	-0.007074
H	0.311228	-1.258438	0.576169
C	1.747188	-0.294572	-0.706730
H	1.835560	0.665430	-1.230776
C	2.920434	-0.437725	0.272219
H	2.815912	-1.400330	0.790154
C	4.282313	-0.386555	-0.416626
H	4.323142	0.487257	-1.080140
H	4.438021	-1.289723	-1.016518
O	5.254608	-0.274906	0.605662
P	6.854028	0.247649	0.139620
O	7.551176	0.290996	1.516413
O	6.626476	1.622525	-0.534001
O	7.353000	-0.859845	-0.820150
O	0.215859	0.803923	0.847850
H	1.048984	0.918886	1.333387

**O**	1.788024	-1.386778	-1.636167
**H**	2.446489	-1.196496	-2.312887
**O**	2.864205	0.627869	1.233476
**H**	3.771944	0.695698	1.576496
**O**	-1.726773	-2.762610	-0.430865
**O**	-5.471781	-3.712203	-0.279289

Table S 9: Coordinates for protonated RQC^{5-}.

ELEMENT	X	Y	Z
C	-3.187297	3.050328	-0.082710
C	-2.376955	1.925862	-0.283495
C	-2.891796	0.633407	-0.324115
C	-4.276979	0.454387	-0.132228
C	-5.078464	1.572232	0.097441
C	-4.562568	2.870645	0.109073
C	-3.921443	-1.927910	0.052120
C	-2.552286	-1.785623	-0.227096
C	-4.499939	-3.127400	0.527222
H	-1.310625	2.066081	-0.375172
H	-6.142917	1.419459	0.244760
C	-5.475290	4.047955	0.341927
H	-5.189209	4.606960	1.237977
H	-5.438754	4.755029	-0.492165
H	-6.510759	3.728316	0.464382
C	-2.572235	4.427690	-0.061329
H	-2.992135	5.070773	-0.841155
H	-2.749684	4.936191	0.891412
H	-1.493724	4.379460	-0.216354
N	-4.807074	-0.828495	-0.239026
N	-2.089666	-0.512349	-0.579204
O	-5.709074	-3.266393	0.792872
C	-0.743072	-0.404657	-1.133634
H	-0.597349	-1.233896	-1.820611
H	-0.683150	0.519562	-1.709193
C	0.393362	-0.444416	-0.108841
H	0.336539	-1.400832	0.421262
C	1.765231	-0.344852	-0.801293
H	1.852986	0.660726	-1.231349
C	2.934024	-0.583979	0.163555
H	2.845603	-1.606831	0.552815
C	4.299584	-0.417244	-0.497955
H	4.332298	0.548286	-1.019299
H	4.468108	-1.216911	-1.227609
O	5.265151	-0.455434	0.536623
P	6.806478	0.304541	0.224381
O	7.533254	0.034071	1.559191
O	6.450449	1.786224	-0.048145
O	7.364845	-0.453303	-1.004389
O	0.217341	0.639091	0.807823
H	1.038786	0.711738	1.320966

**O**	1.808003	-1.342590	-1.831655
**H**	2.470668	-1.090173	-2.483564
**O**	2.846875	0.348128	1.252404
**H**	3.749762	0.392018	1.610981
**O**	-1.717525	-2.743828	-0.177994
**O**	-3.666008	-4.202767	0.690741
**H**	-2.763103	-3.895919	0.390858
**H**	-5.713099	-0.956375	0.191335

Table S 10: Coordinates for protonated RQC^{5-}.

ELEMENT	X	Y	Z
C	-3.320149	3.060020	0.020616
C	-2.464943	1.920777	-0.005545
C	-2.936079	0.623472	-0.096027
C	-4.359154	0.348496	-0.066072
C	-5.192252	1.523259	-0.098062
C	-4.698094	2.831492	-0.042842
C	-3.977534	-2.000947	0.085461
C	-2.593152	-1.857793	-0.128014
C	-4.623176	-3.308889	0.370892
H	-1.394852	2.080167	0.043261
H	-6.266858	1.360740	-0.146595
C	-5.663340	3.996463	-0.059682
H	-5.565918	4.619941	0.836016
H	-5.487692	4.659711	-0.914175
H	-6.698800	3.653705	-0.113248
C	-2.737766	4.449435	0.109264
H	-3.033582	5.094174	-0.729937
H	-3.037392	4.988107	1.019335
H	-1.645158	4.411485	0.109755
N	-4.863146	-0.871589	-0.018380
N	-2.095221	-0.504693	-0.276136
O	-3.925663	-4.317819	0.750219
C	-0.790956	-0.346755	-0.894385
H	-0.653856	-1.133293	-1.633597
H	-0.765045	0.618358	-1.408577
C	0.404353	-0.423543	0.060288
H	0.378686	-1.408069	0.539847
C	1.744852	-0.267555	-0.683229
H	1.801864	0.761227	-1.061121
C	2.962210	-0.542307	0.211813
H	2.901054	-1.585270	0.549739
C	4.295258	-0.327099	-0.501259
H	4.294992	0.659500	-0.983255
H	4.444376	-1.092778	-1.270431
O	5.304179	-0.392558	0.490130
P	6.875455	0.250403	0.089831
O	7.618630	0.042589	1.427190
O	6.602319	1.725244	-0.293757
O	7.360692	-0.634790	-1.083965
O	0.275348	0.609015	1.047335
H	1.119688	0.651438	1.523658

**O**	1.764484	-1.205233	-1.771563
**H**	2.399696	-0.903271	-2.429570
**O**	2.924324	0.328372	1.354127
**H**	3.843806	0.357000	1.669655
**O**	-1.694021	-2.750737	-0.242934
**O**	-5.900134	-3.402343	0.249981
